# Total Synthesis and Biological Evaluation of Modified Ilamycin Derivatives

**DOI:** 10.3390/md20100632

**Published:** 2022-10-03

**Authors:** Jennifer Greve, Axel Mogk, Uli Kazmaier

**Affiliations:** 1 Organic Chemistry, Saarland University, Campus Building C4.2, 66123 Saarbruecken, Germany; 2 Center for Molecular Biology of Heidelberg University (ZMBH), DKFZ-ZMBH Alliance, Im Neuenheimer Feld 282, 69120 Heidelberg, Germany; 3 German Cancer Research Center (DKFZ), Im Neuenheimer Feld 280, 69120 Heidelberg, Germany

**Keywords:** ilamycins, tuberculosis, cyclopeptides, total synthesis, natural products, ClpC1, ATPase

## Abstract

Ilamycins/rufomycins are marine cycloheptapeptides containing unusual amino acids. Produced by *Streptomyces* sp., these compounds show potent activity against a range of mycobacteria, including multidrug-resistant strains of *Mycobacterium tuberculosis*. The cyclic peptides target the AAA+ protein ClpC1 that, together with the peptidases ClpP1/ClpP2, forms an essential ATP-driven protease. Derivatives of the ilamycins with a simplified tryptophane unit are synthesized in a straightforward manner. The ilamycin derivative **26** with a cyclic hemiaminal structure is active in the nM-range against several mycobacterial strains and shows no significant cytotoxicity. In contrast, derivative **27**, with a glutamic acid at this position, is significantly less active, with MICs in the mid µM-range. Detailed investigations of the mode of action of **26** indicate that **26** deregulates ClpC1 activity and strongly enhances ClpC1-WT ATPase activity. The consequences of **26** on ClpC1 proteolytic activities were substrate-specific, suggesting dual effects of **26** on ClpC1-WT function. The positive effect relates to ClpC1-WT ATPase activation, and the negative to competition with substrates for binding to the ClpC1 NTD.

## 1. Introduction

Marine organisms are a rich source of natural products, producing a plethora of fascinating new chemical structures [1,2]. Many of these natural products show interesting biological activities, e.g., against cancer cell lines or bacteria, making them good candidates for drug development against infectious diseases such as tuberculosis, among others [3,4,5]. Tuberculosis is a serious health issue: in 2019, approximately 10 million people fell ill with the disease, and 1.5 million died [6]. A major problem is the continuous development of resistant strains of *Mycobacterium tuberculosis*, the bacteria responsible for this disease. In 2018, 500,000 people demonstrated resistance to the most effective first-line drug rifampicin, and 80% of these suffered from multidrug-resistant tuberculosis (MDR-TB). This clearly illustrates the high demand for new drugs that would work via new modes of action against the largely drug-resistant tuberculosis (XDR-TB) strains, which are resistant to almost all known drugs [7]. In this context, natural products are excellent candidates for developing new anti-TB drugs, since more than 60% of drugs under current development are natural products or derived from natural products [8,9,10].

**Figure 1:**
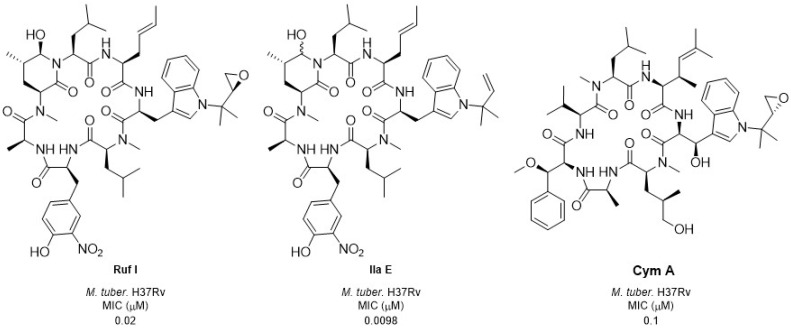
Selected ilamycins/rufomycins and cyclomarins.

In 1962, Japanese researchers isolated interesting cyclic peptides from marine *Streptomyces* sp. that showed interesting activities against *Mycobacteria.* Takita et al. isolated two antibiotics, named ilamycin A and B, from the culture filtrate of *Streptomyces insulates* (A-165-Z1), a new strain later renamed as *Streptomyces islandicus*, that inhibited the growth of *Mycobacterium* 607 and *Mycobacterium phlei* [11,12]. In the same year, Shibata et al. isolated two new antibiotics, rufomycin A and B, from the new strain *Streptomyces atratus* (46408) [13,14], found to be especially active against *Mycobacterium tuberculosis* and *Mycobacterium smegmatis* but almost inactive against most other Gram-positive and Gram-negative bacteria, fungi, and yeasts. Further structural investigations indicated that these two classes of antibiotics possess very similar chemical structures [15,16,17,18,19,20,21]. Very recently, a wide range of new ilamycins/rufomycins were described, differing mainly in their combination of different amino acid oxidation levels and the *N*-prenyl substituent of the tryptophan subunit (alkene, epoxide, diol) [22]. In addition, a total of 18 new Ilamycin F (Ila F) derivatives were obtained by semi-synthesis from Ila F through modification of the nitrotyrosine as well as δ-hydroxyleucine unit [23]. Ilamycin E (Ila E) has been found to be one of the most active examples so far (MIC: ~10 nM) (Figure 1), being more active than the rifampicin used as a positive control [22,23,24,25]. Structure–activity relationship (SAR) studies of the isolated derivatives indicated that cyclized compounds such as Ila E are more active than open-chain leucine derivatives. Oxidation of the prenyl side chain did not obviously affect activity, but the nitro group on the tyrosine seems to play an important role [22,23,24].

In 1999, the groups of Fenical and Clardy reported the isolation of three cyclomarins (Cym) A−C from extracts of a *Streptomyces* sp. (CNB-982) [26]. These compounds are structurally related to the ilamycins/rufomycins. Very similar amino acid building blocks are incorporated, but, interestingly, in a different sequence. As in the ilamycins, an *N*-prenylated tryptophan (CymC) is found as a unique building block that can also be epoxidized (CymA), as in Ruf I, but in the case of the cyclomarins these tryptophan units are β-hydroxylated (Figure 1). At the *N*-terminus of the tryptophan, a γ,δ-unsaturated amino acid is also incorporated and one of the leucines is also oxidized at the δ position, but at another position, as in the ilamycins. Most obvious is the replacement of the unique nitrotyrosine by another aromatic amino acid, *syn*-β-methoxyphenylalanine. Some more, slightly modified, derivatives have been isolated in recent years [27,28]. The cyclomarins were also found to be active against *Mycobacterium tuberculosis* (*M. tub.*) in the higher nM range [29,30,31].

Their excellent biological activities drove the development of syntheses of these interesting cyclic peptides. So far, only one synthesis has been reported for the ilamycins E1 and F, by Guo and Ye et al. in 2018, closing the macrolactame ring between the tryptophan unit and the unsaturated amino acid [32]. The first synthesis of CymC was reported in 2004 by Yao and colleagues [33]. They failed in their attempts to close the peptide ring at the same position [34] but were successful between the unsaturated amino acid and the *N*-methylated leucine [33]. 

Unfortunately, CymA as a natural product lacks satisfactory pharmacokinetic properties, making it challenging for optimization into an (orally) bioavailable drug. Therefore, we decided to develop an independent synthetic route towards the cyclomarins [35,36] and derivatives for SAR studies [37], with the goal of simplifying the rather complex structure without losing too much biological activity [38,39]. Because we were interested to modify the cyclomarins mainly at the tryptophan unit and the unsaturated amino acid, we decided to incorporate these two building blocks at the end of the linear peptide synthesis and to undertake the ring closure at the same position as Yao et al. [33]. Interestingly, neither the epoxide nor the prenyl substituent are required for good activity [39]. Interestingly, the β-OH-functionality can also be removed [38], which allowed a significant simplification of the synthesis, because the β-hydroxytryptophan was found to be extremely sensitive to acid as well as base, which caused severe problems during natural product synthesis. Without the β-OH-functionality, this building block is the same as in the ilamycins. 

## 2. Results and Discussion

Based on these results, we started to develop a synthesis of simplified ilamycin derivatives and to investigate their biological properties. We decided to use the same ring closing position as in the previous cyclomarin synthesis [35,36]. The *N*-prenylated tryptophan was replaced by an *N*-methylated analogue, since this structural change had no significant influence on the activity in the case of the cyclomarins and since *N*-methyl tryptophan is commercially available. We decided to use *N*-Alloc-protected amino acids as building blocks [40,41] in the linear peptide synthesis, because this protecting group can be removed via Pd-catalysis [42] and is therefore compatible with the other protecting groups used during the synthesis.

### 2.1. Preparation of the Amino Acid Building Blocks

**Scheme 1:**
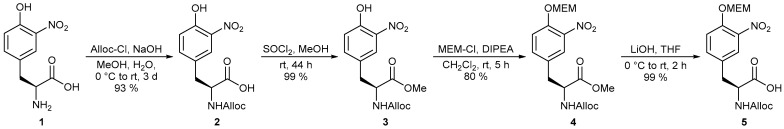
Synthesis of tyrosine building block **5**.

The OH-functionality of the commercially available nitrotyrosine (**1**) had to be protected to avoid side reactions during subsequent peptide coupling steps. In a previous synthesis we used an allyl protecting group [43,44], which can easily be removed with ruthenium catalysts [45], but because we were not sure if this protecting group would create problems during Alloc deprotection we decided to use a methoxymethyl (MEM) protecting group in this case (Scheme 1). After *N*-Alloc protection of **1** [46] the carboxylic acid **2** was converted into methyl ester **3** to allow regioselective MEM-protection of the phenolic OH-functionality [47]. Subsequent saponification of **4** provided the desired amino acid building block **5** in an overall very high yield.

**Scheme 2:**
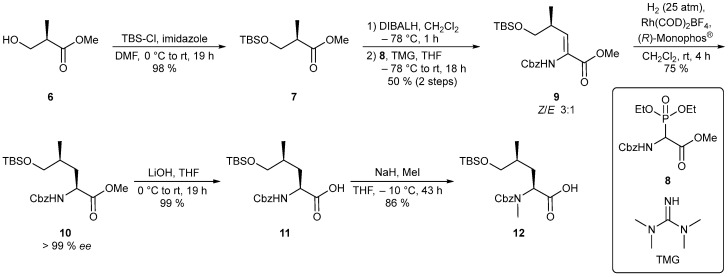
Synthesis of protected hydroxyleucine building block **12**.

The desired *N*-methylated δ-hydroxyleucine was prepared from commercially available (*R*)-Roche ester **6** (Scheme 2). After TBS-protection the ester functionality of **7** was reduced with DIBALH and the aldehyde formed was directly reacted with Schmidt’s phosphonoglycinate **8**, giving rise to the desired dehydroamino acid ester **9** as a 3:1 *Z*/*E*-mixture [48]. A separation of the two isomers was not required in this case, because in the next step (the asymmetric catalytic hydrogenation with (*R*)-Monophos^®^ [49,50]) the *Z*-isomer reacted much faster, almost exclusively generating the desired amino acid ester **10** as a single stereoisomer. Saponification of the methyl ester and subsequent *N*-methylation of **11** under standard conditions [51] delivered the required building block **12**.

**Scheme 3:**
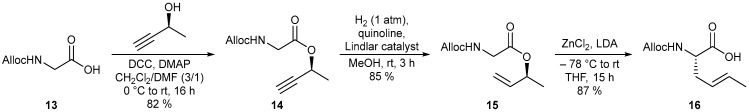
Synthesis of unsaturated amino acid building block **16**.

The unique unsaturated 2-amino-4 hexenoic acid was also synthesized in Alloc-protected form from glycine derivative **13** and commercially available (*S*)-but-3-yn-2-ol using a modified Steglich protocol (Scheme 3) [52]. Lindlar hydrogenation of **14** gave rise to allyl ester **15**, which was subjected to a chelated ester enolate Claisen rearrangement [53,54]. Based on a chair like transition state a perfect chirality transfer from the allyl alcohol to the α-stereogenic center of the amino acid **16** was observed [55,56]. This approach allows the introduction of all kinds of γ,δ-unsaturated side chains.

### 2.2. Synthesis of the Linear Heptapeptide

**Scheme 4:**
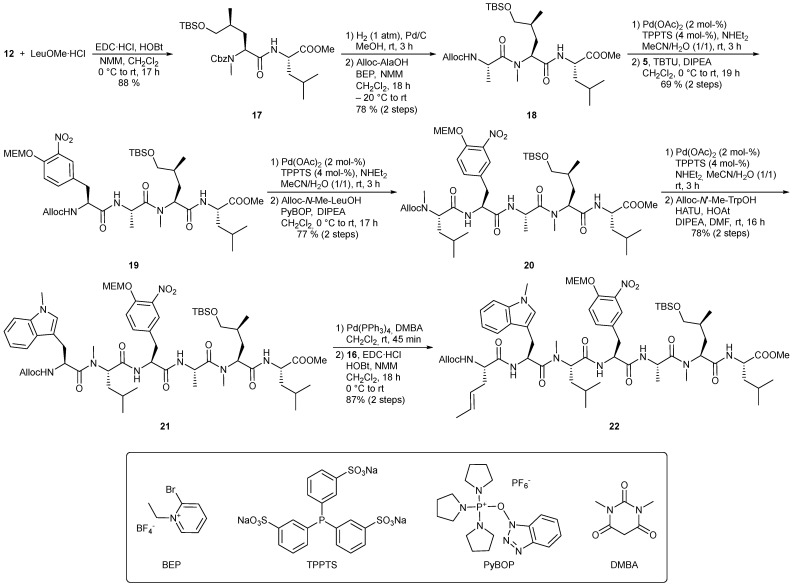
Synthesis of linear heptapeptide **22**.

With the suitable protected building blocks in hand, we started the synthesis of the linear peptide chain with the coupling of hydroxyleucine derivative **12** with Leu-OMe to **17** (Scheme 4). Subsequent cleavage of the Cbz-protecting group and coupling with Alloc-Ala-OH provided tripeptide **18** in acceptable yield. While EDC/HOBt was used as a coupling reagent in the first step, BEP was found to be the reagent of choice in the second step. Palladium-catalyzed removal of the Alloc protecting group using the water-soluble phosphine ligand TPPTS [57,58] provided the free tripeptide which was reacted with the protected nitrotyrosine **5** to tetrapeptide **19**. The nitrotyrosine was found to be the most critical building block of the whole series. Both couplings, at the *C*- as well as on the *N*-terminus, required an optimization of the reaction conditions to obtain good yields. Activation of **5** with TBTU/DIPEA was the method of choice to obtain **19**, and the best results in the subsequent coupling with *N*-MeLeu to **20** were obtained with PyBOP/DIPEA [59]. The continuing prolongation of the peptide chain provided no further problems and the linear heptapeptide **22** was subjected to ring closing reaction. Interestingly, cleavage of the Alloc-protecting group from **21** under the usual conditions gave only a moderate yield, but it could be improved significantly by using dimethylbarbiturate (DMBA) as the cleaving reagent [60,61,62].

### 2.3. Synthesis of Cyclic Ilamycin Derivatives

**Scheme 5:**
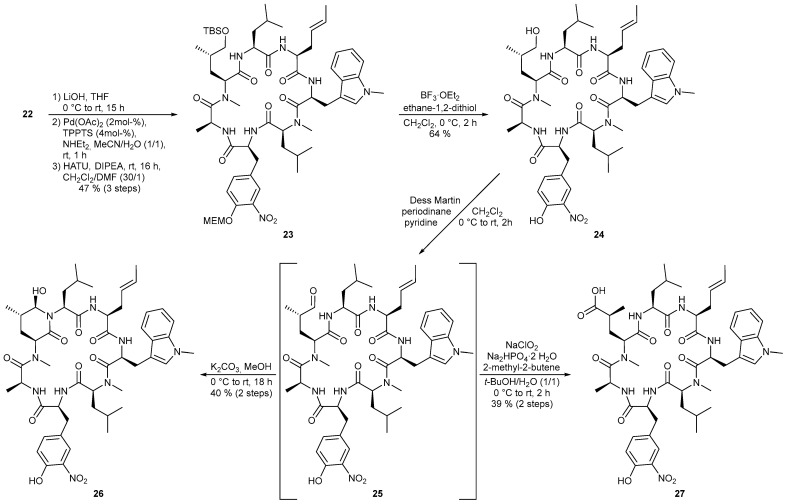
Synthesis of cyclic ilamycin derivatives.

After saponification of the *C*-terminal ester moiety of **22** and removal of the *N*-terminal Alloc protecting group, the cyclization was performed using HATU/DIPEA, providing the desired cyclopeptide **23** in an acceptable yield of 47% over the last three steps (Scheme 5). The final TBS and MEM-deprotection was performed with ethanedithiol and BF_3_ etherate [63], before the hydroxyleucine subunit of **24** was oxidized to the desired ilamycin derivatives. Dess–Martin periodinane oxidation [64] in the presence of an excess pyridine provided aldehyde **25** as an intermediate, which could be converted either into hemiaminal **26** or further oxidized under Pinnick conditions [65] to carboxylic acid **27**.

## 3. Biological Activity and Mode of Action

### 3.1. Biological Evaluation

The new ilamycin derivatives were tested against several mycobacterial strains for anti-mycobacterial activity. The growth inhibition was determined by a resazurin reduction microtiter assay (REMA) [66], and the minimal inhibitory concentrations (MICs) are summarized in Table 1. Compound **26** with the hemiaminal subunit was found to be significantly more active than derivative **27** with the carboxylic acid side chain. While the latter derivative showed MICs in the mid-micromolar range for all tested mycobacterial strains, the highest activity for **26** was observed against *M. tuberculosis* H37Ra strain (MIC: 50 nM). Although **26** was less active compared to the most active ilamycin E, it was twice as active as cyclomarin A. Only moderate cytotoxicity against cancer cell lines (e.g., HepG2) was observed.

### 3.2. Mode of Action

**Figure 2:**
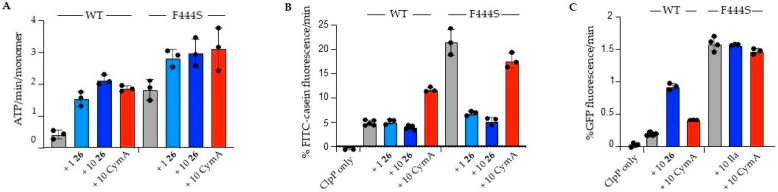
Ilamycin derivative **26** deregulates ClpC1 activities. (**A**) ATPase activities of ClpC1-WT and ClpC1-F444S were determined in the absence (grey column) and presence of indicated components (1, 10 μM **26**; 10 μM cyclomarin A (CymA)). (**B**) FITC–casein degradation by ClpC1-WT and ClpC1-F444S was determined in presence of ClpP and without (grey) or with indicated components (as in (**A**)). Degradation rates were determined from the initial linear increase in FITC fluorescence. (**C**) GFP-SsrA degradation by ClpC1-WT and ClpC1-F444S was determined in presence of ClpP and without (grey) or with indicated components (10 μM **26** or CymA). GFP-SsrA degradation rates were determined from the initial linear decrease in GFP-SsrA fluorescence.

To elucidate the molecular basis of the antibacterial activity of the potent ilamycin derivative **26**, we determined its impact on ATPase and proteolytic activities of its cellular target ClpC1. ClpC1 is an AAA+ protein that forms, together with the peptidase complex ClpP1/ClpP2, an essential ATP-dependent protease in *M. tuberculosis* (Mtb). ClpC1 is regulated by the formation of alternative assembly states, which differ in activity. ClpC1 wild-type (WT) mostly forms an inactive decameric resting state and therefore exhibits low ATPase and proteolytic activity [67]. Resting state formation is mediated by interactions of the ClpC1 coiled-coil M-domains (MD) and is abrogated in the ClpC1 MD mutant F444S, which constitutively forms hexamers and exhibits high activity [67]. We analyzed the impact of **26** on both ClpC1-WT and ClpC1-F444S and used cyclomarin A (CymA), which activates ATPase and proteolytic activities of ClpC1-WT, as a reference. **26** enhanced the ATPase activity of ClpC1-WT up to 5.2-fold, similar to CymA (Figure 2A). The elevated basal ATPase activity of ClpC1-F444S was also increased by both cyclic peptides. 

Next, we monitored the consequences of **26** on the degradation of the disordered model substrate FITC-casein. We used *S. aureus* ClpP (SaClpP) as a cooperating peptidase, which functions with ClpC1 similarly to Mtb ClpP1/P2, but without requiring a dipeptide activator [67]. **26** did not enhance FITC-casein degradation by ClpC1/SaClpP in contrast to CymA, but caused a minor, 1.24-fold reduction at 10 µM concentration (Figure 2B, Supporting informations, Figure S1A). Notably, **26** strongly inhibited the fast FITC-casein degradation by ClpC1-F444S/SaClpP, whereas CymA hardly changed degradation kinetics. These findings indicate fundamental differences in the consequences of **26** and CymA on ClpC1 proteolytic activity, although both compounds enhance ClpC1 ATPase activity to comparable degrees. ATPase stimulation of ClpC1-WT or ClpC1-F444S by **26** was not affected in the presence of ClpP, excluding an altered impact of **26** on ClpC1/SaClpP complexes (Figure S1B). Cyclic peptides bind to a hydrophobic groove of the N-terminal domain (NTD) of ClpC1, a site that has also been identified as a casein binding site in ClpB, a close homolog of ClpC [68]. This raised the possibility that **26** inhibits FITC-casein degradation by ClpC1-F444S by competing with the substrate for binding to the NTD groove. We monitored the binding of FITC-casein to ClpC1-F444S by anisotropy measurements in the absence and presence of **26**. Binding of FITC-casein was 2-fold reduced upon addition of **26**, while CymA had no effect (Figure S1C). This suggests that reduced binding of FITC-casein to ClpC1-F444S contributes to the inhibitory effect of **26** on substrate degradation. Additionally, **26** might modulate NTD dynamics and hamper the delivery of NTD-bound FITC-casein to the processing ClpC1 pore site. 

We next determined the consequences of **26** on the degradation of the alternative substrate GFP-SsrA, which harbors the SsrA degron for Hsp100 recognition [69]. GFP-SsrA does not require the NTD for recognition as it directly binds to the pore site of Hsp100 proteins [70]. **26** did not inhibit the fast proteolysis of GFP-SsrA by ClpC1-F444S/SaClpP, demonstrating that its impact on ClpC1-F444S proteolytic activity is substrate-specific. Similarly, **26** strongly (5.3-fold) enhanced degradation of GFP-SsrA by ClpC1-WT/SaClpP and was even more efficient than CymA, which stimulated degradation 2.4-fold (Figure 2C and Figure S1D). Together these findings demonstrate that **26** deregulates ClpC1 proteolytic activities in a substrate-specific manner.

**Figure 3:**
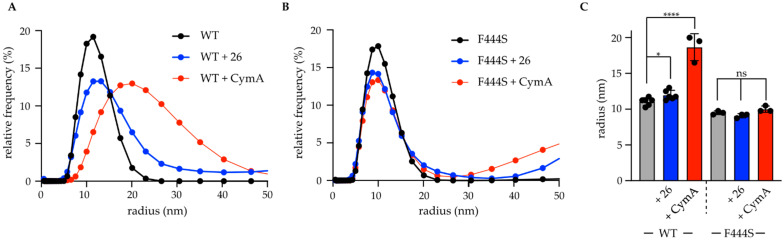
Ilamycin derivative **26** specifically changes the ClpC1-DWB assembly state. (**A**–**C**) Hydrodynamic radii of ClpC1-DWB and ClpC1-DWB-F444S were determined in absence (grey column) or presence of 10 μM **26** or 10 μM cyclomarin A (CymA). (**A**/**B**). The relative frequencies of particle sizes were determined for 0–50 nm. Larger particle sizes were not considered as those were also observed for peptide only controls. (**C**) Radii of peak fractions of indicated ClpC1 assemblies were determined. An unpaired *t*-test (two-tailed) was used to assess the statistical significance of size differences (*n* ≥ 3, *, *p* < 0.05; ****, *p* < 0.0001, ns: not significant).

Finally, we determined how **26** modifies the ClpC1 assembly state by dynamic light scattering (DLS) (Figure 3). Here, we used ATPase-deficient E288A/E626A mutants (termed DWB), which harbor mutations in the Walker B motifs of both ATPase domains, to stabilize assembly states in presence of 2 mM ATP. The hydrodynamic radii of ClpC1-DWB and ClpC1-DWB-F444S were 11.1 ± 0.5 nm and 9.5 ± 0.3 nm, respectively, indicating the presence of a decameric resting state (WT) and hexamers (F444S). Addition of CymA to ClpC1-DWB induced the formation of larger assemblies (18.7 ± 1.9 nm radius), which likely represent the tetrahedral assembly of CymA-bound ClpC1-DWB hexamers as described before [67,71]. Presence of **26** caused a smaller, yet significant increase in the ClpC1-DWB radius to 12.2 ± 0.8 nm (Figure 3A,C). Since **26** increases ClpC1-WT ATPase but also proteolytic activity (GFP-SsrA), we suggest that this assembly state consists of interacting hexamers. No significant changes in radii were observed for ClpC1-DWB-F444S in the presence of the cyclic peptides (Figure 3B,C). This suggests that the change of ClpC1-DWB particle size triggered by **26** involves interactions of the coiled-coil MDs.

### 3.3. Discussion

How does ilamycin exert its antibacterial activity? Here, we show that ilamycin derivative **26** deregulates ClpC1 activity and strongly enhances ClpC1-WT ATPase activity. The consequences on proteolytic activities are diverse and depend on substrate identity. Degradation of FITC-casein, which binds to the NTD like ClpC1 targeting cyclic peptides, remained largely unaffected by **26**. Notably, a strong inhibition of FITC-casein proteolysis by **26** is observed for ClpC1-F444S, which is constitutively hexameric and thus has a high basal ATPase activity. We therefore speculate that **26** has dual effects, negative and positive, on ClpC1-WT function. The positive effect relates to ClpC1-WT ATPase activation, and the negative to substrate binding to the NTD. In case of ClpC1-WT, both effects cancel each other out, while ClpC1-F444S, which is already activated, experiences only the negative impact. This model is consistent with our finding that **26** strongly increases degradation of GFP-SsrA, which does not require NTD for recognition, by ClpC1-WT. Accordingly, GFP-SsrA degradation by activated ClpC1-F444S is not affected by **26**.

Our findings support a model that ClpC1-targeting cyclic peptides in general function by overriding ClpC1 activity control. This explains ClpC1 ATPase activation by lassomycin and ecumicin [72,73]. The consequences of cyclic peptides on substrate degradation in vivo will be diverse and depend on the mechanism of substrate recognition. While we anticipate enhanced degradation of substrates that do not require the ClpC1 NTD for processing, the consequences on NTD-dependent substrates (e.g., FITC-casein) seem diverse, as stimulatory (CymA) but also inhibitory (lassomycin, ecumicin) effects have been described [67,72,73]. Overall, all these peptides bind with similar affinities and in a similar manner to the ClpC1 NTD, though there are differences in their interaction details [74,75,76]. Why peptides can exhibit opposing consequences on substrate degradation is currently unclear. We speculate that peptides might differ in their interaction dynamics or the number of peptide-bound NTDs in a ClpC1 hexamer. We also noticed that **26** induces a specific change in the ClpC1 assembly state that is smaller compared to the tetrahedral hexamers described for CymA-bound ClpC1 [67,71]. We speculate that this state represents a dimer of ClpC1 hexamers, similar to assemblies described for other AAA+ proteins [77,78]. The induction of distinct ClpC1 assembly states triggered by the diverse peptides might also help to explain their specific effects on total ClpC1 activities. 

In summary, we show that **26** can exert opposing effects on ClpC1 proteolytic activities. We suggest that both effects massively change the ClpC1 substrate spectrum and amplify **26** antibacterial potency.

## 4. Materials and Methods

General Synthetic Methods: Air or moisture sensitive reactions were performed in oven-dried reaction flasks (80 °C) under nitrogen or argon atmosphere. Reactions at room temperature were performed at 25 °C. Dry DMF and DCM were purchased from Acros Organics and stored under nitrogen. THF was dried over sodium/benzophenone and diisopropylamine over CaH_2_. Ethyl acetate, pentane and petroleum ether (40–60 °C; PE) were distilled before use. Analytical TLC was carried out with pre-coated silica gel plates from Marcherey Nagel (Polygram^®^ SIL G/UV_254_; Dueren, NRW, Germany). Detection was done using UV light at 254 nm, a KMnO_4_ solution or a ninhydrin solution. LC-MS measurements were carried out either with the following LC unit from Shimadzu (controller: SCL-10Avp, pump: LC-10ATvp, diode array detector SPD-M10Avp; Kyōto, Japan) or with the LC unit LC-2030C 3D Plus, which is also from Shimadzu. The Shimadzu mass spectrometer (LCMS-2020) (Kyōto, Japan) was used for detection in each case. In addition to a Luna^®^ C18 column (50 × 4.6 mm, particle size 3 mm) from Phenomenex (Torrance, CA, USA), an Onxy^®^ Monolithic C18 130 Å column (50 × 4.6 mm) from the same company was used as stationary phase. The crude products were purified either by flash chromatography on silica gel (Macherey-Nagel 60, 0.063–0.2 mm or 0.04–0.063 mm; Dueren, NRW, Germany)) or by using a Büchi Reveleris^®^ Prep Chromatography System (Flawil, Switzerland) with Büchi FlashPure Select C18 (30 mm spherical) columns. Preparative HPLC was also performed on a Büchi Reveleris^®^ Prep Chromatography System using a Phenomenex Luna^®^ C18(2) 100 Å column (250 × 21.1 mm, 5 mm). Melting points were determined in open glass capillaries with a MEL-TEMP II apparatus from Laboratory Devices. The gas chromatographic analysis was done on a gas chromatograph GC 2010 from Shimadzu with a Chirasil-Dex-CB capillary column (25 m length, 0.25 mm inner diameter) from Agilent (Santa Clara, CA, USA). During the measurement, nitrogen served as carrier gas. Optical rotation values were measured using either a Perkin-Elmer polarimeter (model 341; Waltham, MA, USA) or a P-2000 polarimeter from Jasco (Hachiōji, Japan) at the sodium D line (589 nm). All [α]_D_^20^ values are given in 10^−1^ deg cm^2^ g^−1^. ^1^H and ^13^C spectra were recorded with Bruker Avance II 400 [400 MHz (^1^H), 100 MHz (^13^C)], Bruker Avance I 500 or Bruker Avance Neo 500 [500 MHz (^1^H) and 125 MHz (^13^C)] spectrometers in CDCl_3_, MeOD-d_4_ or DMSO-d_6_. CHCl_3_, MeOD-d_3_ or DMSO-d_5_ were used as an internal standard. The chemical shifts are given in ppm (δ) compared to TMS. Peaks were assigned using ^1^H,^1^H COSY, ^1^H,^13^C HSQC, ^1^H,^13^C HMBC and TOCSY spectra. Mass spectra were recorded using a Finnigan MAT 95 sector field spectrometer (HRMS, CI) or a Daltonics maXis 4G hr-ToF spectrometer (HRMS, ESI) from Bruker (Billerica, MA, USA). The Alloc-protected amino acids (Alloc-AlaOH [79], Alloc-*N*-Me-LeuOH [80,81] and Alloc-*N’*-Me-TrpOH [39]) were synthesized according to protocols known from the literature.

General procedure for the Alloc deprotection with Pd(OAc)_2_: To a solution of Alloc-protected peptide (1.0 eq.) in MeCN/H_2_O (1:1, 20 mL/mmol), were added successively diethylamine (5.0 eq.), tris(3-sulfonatophenyl)phosphine hydrate sodium salt (TPPTS; 4 mol-%) and Pd(OAc)_2_ (2 mol-%, 0.02 M in MeCN) at room temperature. After complete conversion (TLC), the solvent was completely removed under reduced pressure.

Strains, plasmids and proteins: Mtb ClpC1-WT and ClpC1-F444S were overproduced from pET24a expression vector in *E. coli* BL21 cells at 30 °C. The proteins were purified using Ni-IDA (Macherey-Nagel) using 50 mM Na-phosphate pH 8.0, 300 mM NaCl, 5 mM β-mercaptoethanol as lysis and washing buffer and the same buffer supplemented with 250 mM imidazole as elution buffer. Elution fractions were pooled and further purified by size exclusion chromatography (Superdex S200, GE Healthcare) in buffer A (50 mM HEPES pH 7.5, 150 mM KCl, 20 mM MgCl_2_, 2 mM DTT, 5% (*v*/*v*) glycerol). Sa ClpP was purified from *E. coli* MC4100 ΔclpB cells after overproduction from pDS56 expression vector. Sa ClpP was purified using the same protocol as Mtb ClpC1. Protein concentrations refer to monomers and were determined with the Bio-Rad Bradford assay using BSA as standard.

ATPase Assay: The ATPase activity of ClpC1 was determined using coupled reactions of pyruvate kinase (PK) and lactate dehydrogenase (LDH) in the presence of phosphoenolpyruvate (PEP) and NADH in buffer A. The reactions included 1 M ClpC1, 1.5 µM Sa ClpP and 1–10 µM **26** or cyclomarin A as indicated. Reactions were started with the injection of 2 mM ATP and changes in NADH absorbance was followed at 340 nm in a BMG Biotech CLARIOstar plate reader. ATPase rates were calculated from the linear decrease in A_340_ in at least three independent experiments and standard deviations were calculated. All reactions included 1% (*v*/*v*) DMSO to equal buffer conditions in presence of **26** or cyclomarin A. The presence of DMSO did not affect ClpC1 activities.

Degradation Assays: All degradation assays were performed in buffer A in presence of an ATP regenerating system (20 ng/μL pyruvate kinase, 3 mM PEP, 2 mM ATP). All reactions included 1% (*v*/*v*) DMSO to equal buffer conditions in presence of **26** or cyclomarin A. 0.1 μM FITC-casein was incubated with 1 µM ClpC1, 1.5 µM Sa ClpP and 1–10 µM **26** or cyclomarin A as indicated. The increase in FITC-casein fluorescence upon its degradation was monitored in BMG Biotech CLARIOstar plate reader by using 483 and 520/530 nm as excitation and emission wavelengths, respectively. For data processing the initial fluorescence intensities were set to 100. FITC-casein degradation rates were determined from the initial slopes of the fluorescence signal increase in at least three independent experiments and standard deviations were calculated. Degradation of 0.5 μM GFP-SsrA was performed in presence of 3 μM ClpC1, 4.5 μM Sa ClpP and 20 μM **26** or cyclomarin A as indicated in a Perkin Elmer LS55 Spectrofluorometer. Degradation was initiated by addition of an ATP regenerating system. GFP fluorescence was monitored by using 400 and 510 nm as excitation and emission wavelengths, respectively. Initial GFP fluorescence was set to 100. Degradation rates were determined from the initial slopes of fluorescence signal decrease in at least three independent experiments and standard deviations were calculated. 

Anisotropy measurements: Binding of ClpC1 to FITC-casein (100 nM) was monitored by fluorescence anisotropy measurements using a BMG Biotech CLARIOstar plate reader. ClpC1 was incubated in buffer A for 5 min at 30 °C in presence of 2 mM ATPγS and 10–20 μM **26** or cyclomarin A as indicated. All reactions included 1% (*v*/*v*) DMSO to equal buffer conditions in presence of **26** or cyclomarin A. Polarization of FITC-casein was determined in black 384 well plates (excitation: 482 nm; emission: 530 nm, Target mP: 35). A sample containing FITC-casein only served as reference. K_d_ values were determined using nonlinear regression curve fitting (Prism 9.3.1, Graphpad Software, San Diego, CA, USA).

Dynamic light scattering measurements: 3 μM ClpC1-WT/F444S were incubated in buffer A with 2 mM ATP and 10 μM **26** or cyclomarin A as indicated. DLS measurements were performed in a Prometheus Pana system (Nanotemper) and particle sizes were determined at 25 °C. Statistical analysis was conducted using Prism 9.3.1.

MIC determination: To determine the inhibitory activity of the derivatives, minimum inhibitory concentration (MIC) was carried out. *M. tuberculosis* (Mtb) strain H37Ra (ATCC 25177), *M. marinum* (Mm) strain M (ATCC BAA-535) and *M. smegmatis* (Msmeg) strain mc^2^155 were cultured in 7H9 complete medium (BD Difco; Becton Dickinson) supplemented with oleic acid-albumin dextrose-catalase (OADC, 10%; BD), 0.4% glycerol, and 0.05% Tween80 at 37 °C as recently described [66]. At mid-log phase (OD600 between 0.4 and 0.8), cultures were harvested and centrifuged (3700× *g*, 10 min). For Mtb and Mm, bacterial cells were then thoroughly resuspended in 7H9 medium (10% OADC) in the absence of glycerol and Tween80 by use of a syringe and a 26-gauge syringe needle. Compounds were tested in 2-fold serial dilutions starting from 64 µM as the highest concentration. MIC values were validated by a Resazurin microtiter assay (REMA) by adding 45 μL of 0,01% Resazurin (Cayman; Ann Arbor, MI, USA) solution to each well, followed by 20 h of incubation without agitation. Fluorescence was measured (excitation at 570 nm and emission at 590 nm) using a microplate reader (Tecan Infinite M200Pro; Tecan, Männedorf, Switzerland)

Cytotoxicity evaluation: HepG2 cells (hepatocellular carcinoma cell line) were cultured under conditions recommended by the depositor. Briefly, cells were seeded at 6 × 103 cells per well of 96-well plates in 180 μL complete medium (DMEM, Sigma-Aldrich, St. Louis, MO, USA). Each compound was tested in a serial dilution as well as the internal solvent control. After 5 d incubation, 20 μL of 5 mg/mL MTT (thiazolyl blue tetrazolium bromide) in PBS was added per well and cells were further incubated for 2 h at 37 °C. The medium was then discarded and cells were washed with 100 μl PBS before adding 100 μL 2-propanol/10 N HCl (250:1) in order to dissolve formazan granules. The absorbance at 570 nm was measured using a microplate reader (Tecan Infinite M200Pro; Tecan, Männedorf, Switzerland), and cell viability was expressed as percentage relative to the respective methanol control. IC50 values were determined by sigmoidal curve fitting.

### 4.1. Synthesis of the Amino Acid Building Blocks

#### 4.1.1. Synthesis of (*S*)-2-{[(allyloxy)carbonyl]amino}-3-(4-hydroxy-3-nitrophenyl)-propa noic Acid (**2**)

Alloc-Cl (ρ = 1.134 g/mL, 0.26 mL, 2.4 mmol) was slowly added to a solution of 3-nitrotyrosine (500 mg, 2.21 mmol) (**1**) in 1.2 mL (4.8 mmol) 4 M aq. NaOH solution at 0 °C. After the reaction mixture had been stirred for 10 min at this temperature, the ice bath was removed and 1.2 mL (4.8 mmol) 4 M aq. NaOH solution and 0.2 mL water were added. Alloc-Cl (94 μL, 0.88 mmol) was added again to the reaction mixture after 1 h at room temperature and 2.4 mL MeOH after 2 h. After three days the solution was diluted with 10 mL sat. aq. NaHCO_3_ solution. The aqueous phase was washed with diethyl ether, cooled to 0 °C and acidified to pH 3 with conc. HCl solution. After the aqueous phase had been extracted three times with EtOAc, the combined organic phases were dried over Na_2_SO_4_ and concentrated under reduced pressure. For further purification, the crude product was lyophilized to yield 639 mg (2.06 mmol, 93%) of Alloc-protected tyrosine (**2**) as a yellow solid. R_f_ = 0.41 (DCM/MeOH 9:1); [α]_D_^20^ = +84.5 (c = 1.0, CHCl_3_); ^1^H-NMR (CDCl_3_, 400 MHz) δ 3.08 (1 H, dd, J = 14.2, 6.5 Hz, H-3), 3.25 (1 H, dd, J = 14.1, 5.3 Hz, H-3’), 4.52–4.60 (2 H, m, H-11), 4.67 (1 H, dt, J = 6.7, 6.4 Hz, H-2), 5.20–5.30 (1 H, m, NH), 5.23 (1 H, d, J = 10.4 Hz, H-13), 5.29 (1 H, d, J = 18.5 Hz, H-13’), 5.89 (1 H, ddt, J = 17.0, 10.6, 5.5 Hz, H-12), 7.11 (1 H, d, J = 8.6 Hz, H-8), 7.43 (1 H, dd, J = 8.7, 1.7 Hz, H-9), 7.93 (1 H, bs, H-5), 10.49 (1 H, s, OH); ^13^C-NMR (CDCl_3_, 100 MHz) δ 36.8 (t, C-3), 54.3 (d, C-2), 66.2 (t, C-11), 118.3 (t, C-13), 120.3 (d, C-8), 125.4 (d, C-5), 128.1 (s, C-4), 132.2 (d, C-12), 133.4 (s, C-6), 138.7 (d, C-9), 154.3 (s, C-7, C-10), 174.8 (s, C-1); HRMS (CI) *m*/*z* calculated for C_13_H_15_N_2_O_7_ [M + H]^+^ 311.0874, found 311.0881.

#### 4.1.2. Synthesis of Methyl (*S*)-2-{[(allyloxy)carbonyl]amino}-3-(4-hydroxy-3-nitrophenyl)-propanoate (**3**)

To a solution of tyrosine derivative **2** (200 mg, 650 μmol) in 1.3 mL MeOH was slowly added thionyl chloride (57 μL, 0.77 mmol) at room temperature. Subsequently, stirring was continued overnight at this temperature. After 44 h (TLC), the solvent was removed under reduced pressure and the crude product was purified by flash chromatography (silica gel, DCM/MeOH 99:1). The desired methyl ester (**3**) (209 mg, 640 μmol, quant., mp: 70 °C) could be isolated as a yellow solid. R_f_ = 0.37 (DCM/MeOH 99:1); [α]_D_^20^ = +66.5 (c = 1.0, CHCl_3_); ^1^H-NMR (CDCl_3_, 400 MHz) δ 3.04 (1 H, dd, J = 14.1, 6.0 Hz, H-3), 3.18 (1 H, dd, J = 14.1, 5.4 Hz, H-3), 3.76 (3 H, s, H-14), 4.50–4.60 (2 H, m, H-11), 4.63 (1 H, dt, J = 7.0, 6.6 Hz, H-2), 5.22 (1 H, dd, J = 10.4, 1.2 Hz, H-13), 5.24–5.35 (1 H, m, NH), 5.29 (1 H, d, J = 16.0 Hz, H-13), 5.89 (1 H, ddt, J = 17.1, 10.6, 5.4 Hz, H-12), 7.09 (1 H, d, J = 8.6 Hz, H-8), 7.37 (1 H, dd, J = 8.6, 2.2 Hz, H-9), 7.87 (1 H, d, J = 2.0 Hz, H-5), 10.49 (1 H, s, OH); ^13^C-NMR (CDCl_3_, 100 MHz) δ 37.2 (t, C-3), 52.6 (q, C-14), 54.6 (d, C-2), 66.0 (t, C-11), 118.1 (t, C-13), 120.2 (d, C-8), 125.2 (d, C-5), 128.4 (s, C-4), 132.4 (d, C-12), 133.3 (s, C-6), 138.6 (d, C-9), 154.2 (s, C-7), 155.4 (s, C-10), 171.4 (s, C-1); HRMS (CI) *m*/*z* calculated for C_14_H_17_N_2_O_7_ [M + H]^+^ 325.1030, found 325.1041.

#### 4.1.3. Synthesis of Methyl (*S*)-2-{[(allyloxy)carbonyl]amino}-3-{4-[(2-methoxyethoxy)met hoxy]-3-nitrophenyl}propanoate (**4**)

To a solution of tyrosine (**3**) (300 mg, 930 μmol) in 2.4 mL DCM was added successively DIPEA (339 µL, 1.94 mmol) and MEM-Cl (ρ = 1.09 g/mL, 115 µL, 1.02 mmol) at room temperature. After complete conversion (5 h, TLC), the reaction mixture was diluted with DCM. The organic phase was washed with water and brine, dried over Na_2_SO_4_ and concentrated under reduced pressure. Final purification by flash chromatography (silica gel, PE/EtOAc 1:1) afforded the MEM-protected tyrosine (**4**) (306 mg, 740 μmol, 80%) as a yellow oil. R_f_ = 0.25 (PE/EtOAc 1:1); [α]_D_^20^ = +35.3 (c = 1.0, CHCl_3_); ^1^H-NMR (CDCl_3_, 400 MHz) δ 3.06 (1 H, dd, J = 14.0, 6.0 Hz, H-3), 3.17 (1 H, dd, J = 14.4, 5.2 Hz, H-3’), 3.36 (3 H, s, H-13), 3.53–3.59 (2 H, m, H-12), 3.76 (3 H, s, H-18), 3.84–3.90 (2 H, m, H-11), 4.57 (2 H, d, J = 5.5 Hz, H-15), 4.63 (1 H, dt, J = 7.3, 6.6 Hz, H-2), 5.22 (1 H, dd, J = 10.4, 1.2 Hz, H-17), 5.24–5.31 (1 H, m, NH), 5.29 (1 H, d, J = 17.0 Hz, H-17’), 5.36 (2 H, s, H-10), 5.90 (1 H, ddt, J = 17.0, 10.6, 5.5 Hz, H-16), 7.25–7.33 (2 H, m, H-8, H-9), 7.57 (1 H, bs, H-5); ^13^C-NMR (CDCl_3_, 100 MHz) δ 37.0 (t, C-3), 52.6 (q, C-18), 54.6 (d, C-2), 59.0 (q, C-13), 66.0 (t, C-15), 68.4 (t, C-11), 71.4 (t, C-12), 94.3 (t, C-10), 117.5 (t, C-17), 118.1 (d, C-8), 125.9 (d, C-5), 129.7 (s, C-4), 132.4 (d, C-16), 134.8 (d, C-9), 140.4 (s, C-6), 149.5 (s, C-7), 155.4 (s, C-14), 171.4 (s, C-1); HRMS (CI) *m*/*z* calculated for C_18_H_25_N_2_O_9_ [M+H]^+^ 413.1555, found 413.1571.

#### 4.1.4. Synthesis of (*S*)-2-{[(allyloxy)carbonyl]amino}-3-{4-[(2-methoxyethoxy)methoxy]-3-nitro-phenyl}propanoic Acid (**5**)

To a solution of methyl ester (**4**) (4.82 g, 11.7 mmol) in 146 mL THF was added dropwise a 1 M aq. LiOH solution (14.0 mL, 14.0 mmol) at 0 °C. During the reaction, the reaction mixture was slowly warmed to room temperature (2 h). The solvent was removed in vacuo. The residue was dissolved in water and the aqueous phase was acidified with 1 M aq. KHSO_4_ solution to pH 3–4. Subsequently, the aqueous phase was extracted three times with DCM. After the combined organic phases had been dried over Na_2_SO_4_, the solvent was removed under reduced pressure to obtain **5** (4.66 g, 11.7 mmol, quant.) as a yellow resin. R_f_ = 0.28 (DCM/MeOH 9:1); [α]_D_^20^ = +59.6 (c = 1.0, CHCl_3_); Major rotamer: ^1^H-NMR (CDCl_3_, 400 MHz) δ 3.09 (1 H, dd, J = 14.2, 6.0 Hz, H-3), 3.23 (1 H, dd, J = 14.3, 5.1 Hz, H-3’), 3.36 (3 H, s, H-13), 3.55–3.61 (2 H, m, H-12), 3.84–3.91 (2 H, m, H-11), 4.57 (2 H, d, J = 4.9 Hz, H-15), 4.66 (1 H, dt, J = 7.0, 6.4 Hz, H-2), 5.23 (1 H, dd, J = 10.4, 1.1 Hz, H-17), 5.25–5.39 (1 H, m, NH), 5.30 (1 H, d, J = 16.9 Hz, H-17’), 5.36 (2 H, s, H-10), 5.89 (1 H, ddt, J = 17.2, 10.6, 5.5 Hz, H-16), 6.15 (1 H, bs, COOH), 7.29 (1 H, d, J = 8.7 Hz, H-8), 7.34 (1 H, dd, J = 8.7, 2.1 Hz, H-9), 7.63 (1 H, bs, H-5); Minor rotamer (selected signals): ^1^H-NMR (CDCl_3_, 400 MHz) δ 2.99–3.14 (1 H, m, H-3), 3.15–3.27 (1 H, m, H-3’), 4.48–4.60 (2 H, m, H-15), 7.64–7.70 (1 H, m, H-5); ^13^C-NMR (CDCl_3_, 100 MHz) δ 36.6 (t, C-3), 54.3 (d, C-2), 58.9 (q, C-13), 66.1 (t, C-15), 68.3 (t, C-11), 71.4 (t, C-12), 94.3 (t, C-10), 117.6 (t, C-17), 118.2 (d, C-8), 126.0 (d, C-5), 129.7 (s, C-4), 132.3 (d, C-16), 134.9 (d, C-9), 140.4 (s, C-6), 149.5 (s, C-7), 155.7 (s, C-14), 174.1 (s, C-1); HRMS (CI) *m*/*z* calculated for C_17_H_23_N_2_O_9_ [M + H]^+^ 399.1398, found 399.1396.

#### 4.1.5. Synthesis of Methyl (*R*)-3-[(*tert*-butyldimethylsilyl)oxy]-2-methylpropanoate (**7**) [82]

To a solution of (*R*)-Roche ester (**6**) (5.00 g, 42.3 mmol) in anhydrous DMF was added imidazole (3.31 g, 48.7 mmol) at room temperature. Subsequent addition of TBS-Cl (7.02 g, 46.6 mmol) was carried out at 0 °C. The reaction mixture was slowly warmed to room temperature overnight. After 19 h (TLC), the solution was dissolved in diethyl ether. The organic phase was washed successively with water and brine and dried over Na_2_SO_4_. After the solvent was removed under reduced pressure, the crude product was purified by flash chromatography (silica gel, PE/Et_2_O 95:5). The TBS-protected Roche ester (**7**) (9.63 g, 41.4 mmol, 98%) was obtained as a colorless liquid. R_f_ = 0.29 (PE/Et_2_O 95:5); [α]_D_^20^ = −21.5 (c = 1.0, CHCl_3_); ^1^H-NMR (CDCl_3_, 400 MHz) δ 0.03 (3 H, s, H-4), 0.04 (3 H, s, H-4’), 0.87 (9 H, s, H-6), 1.14 (3 H, d, J = 7.0 Hz, H-7), 2.65 (1 H, ddq, J = 6.8, 6.8, 6.8 Hz, H-2), 3.65 (1 H, dd, J = 9.7, 6.0 Hz, H-3), 3.67 (3 H, s, H-8), 3.77 (1 H, dd, J = 9.7, 6.9 Hz, H-3’); ^13^C-NMR (CDCl_3_, 100 MHz) δ -5.5 (q, C-4, C-4’), 13.5 (q, C-7), 18.2 (s, C-5), 25.8 (q, C-6), 42.5 (d, C-2), 51.5 (q, C-8), 65.2 (t, C-3), 175.5 (s, C-1).

#### 4.1.6. Synthesis of Methyl (*S*,*Z*)-2-{[(benzyloxy)carbonyl]amino}-5-[(*tert*-butyldimethylsilyl)oxy]-4-methylpent-2-enoate (**9**)

To a solution of TBS-protected Roche ester (**7**) (9.80 g, 42.2 mmol) in 422 mL anhydrous DCM, a DIBALH solution (ρ = 0.701 g/mL, 46.4 mL, 46.4 mmol, 1 M in hexane) was slowly added at –78 °C within 60 min. Stirring was continued at this temperature for 40 min. After complete conversion (1 h, TLC), 21 mL of MeOH and 120 mL of a 10% Na/K tartrate solution were added to the reaction mixture at –78 °C. The cooling bath was removed and the reaction mixture was allowed to warm to room temperature. After the DCM layer became clear, the phases were separated and the aqueous phase was extracted three times with DCM. The combined organic phases were washed with 1 M aq. KHSO_4_ solution as well as with sat. aq. NaHCO_3_ solution and dried over Na_2_SO_4_. The solvent was removed in vacuo and the crude aldehyde was dissolved in 320 mL dry THF. The Cbz-protected phosphonate (**8**) (16.7 g, 46.4 mmol) was dissolved in 100 mL anhydrous THF and cooled to –78 °C. Tetramethylguanidine (ρ = 0.918 g/mL, 5.6 mL, 44 mmol) was added. After the solution was stirred for 20 min at this temperature, the aldehyde solution was slowly added. Subsequently the reaction mixture was warmed to room temperature overnight. The solution was diluted with diethyl ether and water. The aqueous phase was extracted three times with diethyl ether and the combined organic phases were dried over Na_2_SO_4_. The solvent was removed in vacuo. After purification by flash chromatography (silica gel, PE/EtOAc 8:2), 8.57 g (21.0 mmol, 50 %, 75 % *Z* according to ^1^H-NMR) of dehydroamino acid (**9**) was obtained as a colorless liquid. R_f_ = 0.28 (PE/EtOAc 8:2); [α]_D_^20^ = +63.0 (c = 1.0, CHCl_3_); Mixture of configuration isomers: (*Z*)-**9**: ^1^H-NMR (CDCl_3_, 400 MHz) δ 0.03 (3 H, s, H-6), 0.05 (3 H, s, H-6’), 0.88 (9 H, s, H-8), 1.01 (3 H, d, J = 6.7 Hz, H-9), 2.74–2.88 (1 H, m, H-4), 3.42 (1 H, dd, J = 9.2, 9.2 Hz, H-5), 3.64 (1 H, dd, J = 9.5, 4.7 Hz, H-5’), 3.69–3.81 (3 H, m, H-10), 5.12 (1 H, d, J = 12.4, H-12), 5.17 (1 H, d, J = 12.4 Hz, H-12), 6.20 (1 H, d, J = 9.4 Hz, H-3), 7.01 (1 H, bs, NH), 7.28–7.40 (5 H, m, H-14, H-15, H-16); (*E*)-**9** (selected signals): ^1^H-NMR (CDCl_3_, 400 MHz) δ 0.07 (3 H, s, H-6), 0.10 (3 H, s, H-6’), 0.84 (3 H, d, J = 7.0 Hz, H-9), 0.90 (9 H, s, H-8), 1.88–2.00 (1 H, m, H-4), 3.55 (1 H, dd, J = 9.8, 8.0 Hz, H-5); (*Z*)-**9**: ^13^C-NMR (CDCl_3_, 100 Hz) δ -5.6 (q, C-6), -5.5 (q, C-6’), 15.8 (q, C-9), 18.3 (s, C-7), 25.8 (q, C-8), 35.2 (d, C-4), 52.2 (q, C-10), 67.1 (t, C-12), 68.5 (t, C-5), 128.0 (d, C-14), 128.1 (d, C-16), 128.4 (d, C-15), 136.1 (s, C-13), 137.0 (s, C-2; d, C-3), 154.2 (s, C-11), 165.0 (s, C-1); (*E*)-**9** (selected signals): ^13^C-NMR (CDCl_3_, 100 Hz) δ -3.6 (q, C-6, C-6‘), 13.1 (q, C-9), 25.6 (q, C-8), 37.0 (d, C-4), 52.5 (q, C-10), 68.3 (t, C-12), 68.8 (t, C-5); HRMS (CI) *m*/*z* calculated for C_21_H_34_NO_5_Si [M + H]^+^ 408.2201, found 408.2204.

#### 4.1.7. Synthesis of Methyl (2*S*,4*S*)-2-{[(benzyloxy)carbonyl]amino}-5-[(*tert*-butyldimethylsilyl)oxy]-4-methylpentanoate (**10**)

Rh(COD)_2_BF_4_ (171 mg, 420 μmol) and (*R*)-Monophos^®^ (302 mg, 840 μmol) were dissolved under argon-atmosphere in 76 mL dry DCM. Dehydroamino acid (**9**) (8.58 g, 21.0 mmol) dissolved in 19 mL dry DCM was added. The reaction mixture was stirred in an autoclave under 25 bar H_2_-atmosphere for 4 h at room temperature. The solvent was removed under reduced pressure and the residue was purified by flash chromatography (silica gel, PE/EtOAc 8:2) to afford **10** (6.55 g, 16.0 mmol, 76%, >99% *ee*) as a colorless oil. R_f_ = 0.36 (PE/EtOAc 8:2); [α]_D_^20^ = –12.5 (c = 1.0, CHCl_3_); To determine the enantiomeric excess of amino acid **10**, the Cbz protecting group was replaced by an acetate group to allow gas chromatographic measurement. For this purpose, hydroxyleucine (10) (30 mg, 73 μmol) was dissolved in 0.7 mL dry THF and 10 wt-% Pd-C (3 mg, 10% on activated charcoal) was added to the reaction mixture. Stirring was carried out at 1 bar H_2_ atmosphere for 26 h at room temperature. The mixture was filtered through Celite and the solvent was removed in vacuo. Subsequently, the residue was dissolved in 1.5 mL pyridine followed by the addition of acetic anhydride (ρ = 1.08 g/mL, 10.4 μL, 110 μmol) at room temperature. After 21 h (TLC), the reaction mixture was diluted with EtOAc and the organic phase was washed twice with 1 M aq. HCl solution, dried over Na_2_SO_4_ and concentrated under reduced pressure. The acetate-protected hydroxyleucine (14 mg, 44 μmol, 60%) was obtained as a yellow resin, R_f_ = 0.31 (PE/EtOAc 8:2). GC-FID: Column: Agilent CP-Chirasil-Dex CB; Carrier gas: N_2_; T_0_ [1 min] = 110 °C, 2.0 °C/min to 180 °C, injector: 250 °C, detector: 275 °C; *N*-acetyl-(2*R*,4*S*): t_r_ = 32.42 min, *N*-acetyl-(2*S*,4*S*): t_r_ = 32.76 min; ^1^H-NMR (CDCl_3_, 500 MHz) δ 0.03 (3 H, s, H-6), 0.03 (3 H, s, H-6’), 0.88 (9 H, s, H-8), 0.94 (3 H, d, J = 6.6 Hz, H-9), 1.53–1.62 (1 H, m, H-3), 1.70–1.80 (2 H, m, H-3’, H-4), 3.36 (1 H, dd, J = 9.8, 6.3 Hz, H-5), 3.48 (1 H, dd, J = 9.8, 5.0 Hz, H-5’), 3.73 (3 H, s, H-10), 4.34–4.41 (1 H, m, H-2), 5.10 (2 H, s, H-12), 5.32 (1 H, d, J = 8.2 Hz, NH), 7.29–7.38 (5 H, m, H-14, H-15, H-16); ^13^C-NMR (CDCl_3_, 125 MHz) δ -5.5 (q, C-6), -5.5 (q, C-6’), 16.4 (q, C-9), 18.3 (s, C-7), 25.9 (q, C-8), 32.7 (d, C-4), 36.1 (t, C-3), 52.3 (q, C-10), 52.4 (d, C-2), 66.9 (t, C-12), 67.9 (t, C-5), 128.1 (d, C-14), 128.1 (d, C-16), 128.5 (d, C-15), 136.3 (s, C-13), 156.1 (s, C-11), 173.6 (s, C-1); HRMS (CI) *m*/*z* calculated for C_21_H_36_NO_5_Si [M + H]^+^ 410.2357, found 410.2374.

#### 4.1.8. Synthesis of (2*S*,4*S*)-2-{[(benzyloxy)carbonyl]amino}-5-[(*tert*-butyldimethyl-silyl)oxy]-4-methylpentanoic Acid (**11**)

To a solution of methyl ester **10** (2.35 g, 5.74 mmol) in 57 mL THF was added a 1 M aq. LiOH solution (6.9 mL, 6.9 mmol) at 0 °C. With stirring, the reaction mixture was slowly warmed to room temperature (19 h). The solvent was removed under reduced pressure. The residue dissolved in water and acidified with 1 M aq. KHSO_4_ solution to pH 3. The aqueous phase was extracted three times with DCM. After the combined organic phases were dried over Na_2_SO_4_, the solvent was removed in vacuo again. Without further purification, 2.27 g (5.74 mmol, quant.) of **11** was obtained as a colorless oil. R_f_ = 0.12 (PE/EtOAc 8:2); [α]_D_^20^ = +8.8 (c = 1.0, CHCl_3_); Major rotamer: ^1^H-NMR (CDCl_3_, 400 MHz) δ 0.01–0.12 (6 H, m, H-6, H-6‘), 0.89 (9 H, s, H-8), 0.94 (3 H, d, J = 6.4 Hz, H-9), 1.59–1.73 (1 H, m, H-3), 1.73–1.92 (2 H, m, H-3’, H-4), 3.43 (1 H, dd, J = 9.5, 7.2 Hz, H-5), 3.53–3.59 (1 H, m, H-5’), 4.39–4.49 (1 H, m, H-2), 5.11 (2 H, s, H-11), 5.49 (1 H, d, J = 7.7 Hz, NH), 6.93 (1 H, bs, COOH), 7.28–7.42 (5 H, m, H-13, H-14, H-15); Minor rotamer (selected signals): ^1^H-NMR (CDCl_3_, 400 MHz) δ 4.35 (1 H, bs, H-2), 5.64 (1 H, bs, NH); ^13^C-NMR (CDCl_3_, 100 MHz) δ -5.5 (q, C-6), -5.5 (q, C-6’), 16.8 (q, C-9), 18.3 (s, C-7), 25.9 (q, C-8), 32.6 (d, C-4), 36.9 (t, C-3), 52.4 (d, C-2), 67.0 (t, C-11), 68.5 (t, C-5), 128.0 (d, C-13), 128.1 (d, C-15), 128.5 (d, C-14), 136.2 (s, C-12), 156.2 (s, C-10), 176.2 (s, C-1); HRMS (CI) *m*/*z* calculated for C_20_H_34_NO_5_Si [M + H]^+^ 396.2201, found 396.2210.

#### 4.1.9. Synthesis of (2*S*,4*S*)-2-{[(benzyloxy)carbonyl](methyl)amino}-5-[(*tert*-butyldimethylsilyl)oxy]-4-methylpentanoic Acid (**12**)

Cbz-protected amino acid **11** (5.58 g, 14.1 mmol) was dissolved in 141 mL anhydrous THF. After cooling down to –10 °C, NaH (2.26 g, 56.5 mmol, 60% in mineral oil) was added to the reaction mixture in portions, followed by methyl iodide (ρ = 2.28 g/mL, 7.1 mL, 113 mmol). After 43 h (LCMS) at this temperature, the reaction mixture was diluted with EtOAc and poured onto water. The organic phase was extracted three times with water. The combined aqueous phases were acidified to pH 3 using 1 M aq. KHSO_4_ solution and extracted three times with DCM. Subsequently, the combined organic phases were washed with 5% Na_2_SO_3_ solution and brine and dried over Na_2_SO_4_. The solvent was removed under reduced pressure to obtain the desired *N*-methylated amino acid **12** (4.97 g, 12.1 mmol, 86%) as a yellow oil. [α]_D_^20^ = –4.2 (c = 1.0, CHCl_3_); Major rotamer: ^1^H-NMR (CDCl_3_, 400 MHz) δ 0.04 (6 H, s, H-6), 0.88 (9 H, s, H-8), 0.92 (3 H, d, J = 6.2 Hz, H-9), 1.50–1.60 (1 H, m, H-4), 1.58–1.68 (1 H, m, H-3) 2.01–2.13 (1 H, m, H-3’), 2.89 (3 H, s, H-10), 3.36 (1 H, dd, J = 9.7, 7.3 Hz, H-5), 3.50 (1 H, dd, J = 9.8, 4.9 Hz, H-5’), 5.01 (1 H, dd, J = 11.9, 4.0 Hz, H-2), 5.08–5.23 (2 H, m, H-12), 7.27–7.40 (5 H, m, H-14, H-15, H-16); Minor rotamer (selected signals): ^1^H-NMR (CDCl_3_, 400 MHz) δ 0.02 (6 H, s, H-6), 0.82 (3 H, d, J = 5.8 Hz, H-9), 0.88 (9 H, s, H-8), 2.90 (3 H, s, H-10), 3.32 (1 H, d, J = 9.7, 6.6 Hz, H-5), 3.47 (1 H, dd, J = 11.4, 4.4 Hz, H-5’), 4.85 (1 H, dd, J = 11.5, 3.4 Hz, H-2); Major rotamer: ^13^C-NMR (CDCl_3_, 100 MHz) δ -5.5 (q, C-6), 15.6 (q, C-9), 18.3 (s, C-7), 25.9 (q, C-8), 30.1 (q, C-10), 31.9 (t, C-3), 32.6 (d, C-4), 56.1 (d, C-2), 67.6 (t, C-12), 68.1 (t, C-5), 127.7 (d, C-14), 128.0 (d, C-16), 128.5 (d, C-15), 136.5 (s, C-13), 157.3 (s, C-11), 177.0 (s, C-1); Minor rotamer (selected signals): ^13^C-NMR (CDCl_3_, 100 MHz) δ -5.5 (q, C-6), 15.4 (q, C-9), 18.2 (s, C-7), 30.3 (q, C-10), 32.3 (t, C-3), 32.4 (d, C-4), 55.9 (d, C-2), 67.7 (t, C-12), 68.1 (t, C-5), 127.9 (d, C-14), 128.1 (d, C-16), 128.5 (d, C-15), 136.5 (s, C-13), 156.4 (s, C-11), 177.1 (s, C-1); HRMS (CI) *m*/*z* calculated for C_21_H_36_NO_5_Si [M + H]^+^ 410.2357, found 410.2367.

#### 4.1.10. Synthesis of (S)-But-3-yn-2-yl [(allyloxy)carbonyl]glycinate (**14**)

To a solution of propargyl alcohol (1.61 g, 23.0 mmol) in 23 mL anhydrous DCM was added Alloc glycine (**13**) (4.03 g, 25.3 mmol) and DMAP (141 mg, 1.15 mmol) at room temperature. The reaction mixture was cooled to 0 °C, followed by the addition of DCC (5.23 g, 25.3 mmol) and 8 mL dry DMF. Overnight it was warmed to room temperature (16 h). The reaction mixture was filtered through Celite and the solvent was subsequently removed under reduced pressure. The residue was diluted with EtOAc and the organic phase was washed three times with water and once with brine. After drying over Na_2_SO_4_, the crude product was concentrated under reduced pressure. Flash chromatographic purification (silica gel, PE/EtOAc 8:2) afforded the desired propargyl ester **14** (3.99 g, 18.9 mmol, 82%) as a colorless oil. R_f_ = 0.15 (PE/EtOAc 8:2); [α]_D_^20^ = –95.0 (c = 1.0, CHCl_3_); ^1^H-NMR (CDCl_3_, 400 MHz) δ 1.53 (3 H, d, J = 6.7 Hz, H-7), 2.48 (1 H, d, J = 2.1 Hz, H-10), 3.97 (1 H, dd, J = 18.1, 5.5 Hz, H-2), 4.03 (1 H, dd, J = 18.3, 5.7 Hz, H-2’), 4.59 (2 H, d, J = 5.6 Hz, H-4), 5.19–5.27 (1 H, m, NH), 5.22 (1 H, dd, J = 10.4, 1.2 Hz, H-6), 5.31 (1 H, dd, J = 17.2, 1.5 Hz, H-6’), 5.49 (1 H, dq, J = 6.7, 6.7 Hz, H-8), 5.91 (1 H, ddt, J = 17.2, 10.4, 5.5 Hz, H-5); ^13^C-NMR (CDCl_3_, 100 MHz) δ 21.1 (q, C-7), 42.7 (t, C-2), 61.3 (d, C-8), 66.0 (t, C-4), 73.5 (d, C-10), 81.4 (s, C-9), 117.9 (t, C-6), 132.5 (d, C-5), 156.1 (s, C-3), 169.0 (s, C-1); HRMS (CI) *m*/*z* calculated for C_10_H_14_NO_4_ [M + H]^+^ 212.0917, found 212.0924.

#### 4.1.11. Synthesis of (*S*)-But-3-en-2-yl [(allyloxy)carbonyl]glycinate (**15**)

To a solution of propargyl ester **14** (3.94 g, 18.6 mmol) in 48 mL MeOH were successively added quinoline (ρ = 1.09 g/mL, 0.66 mL, 5.6 mmol) and Lindlar catalyst (272 mg) at room temperature. Subsequently, stirring was performed under 1 atm H_2_ atmosphere for 3 h. The reaction mixture was filtered through Celite, the solvent was removed under reduced pressure and the crude product was purified by flash chromatography (silica gel, PE/EtOAc 9:1 → 85:15). Allyl ester **15** (3.37 g, 15.8 mmol, 85%) was isolated as a colorless oil. R_f_ = 0.22 (PE/EtOAc 8:2); [α]_D_^20^ = –25.3 (c = 1.0, CHCl_3_); ^1^H-NMR (CDCl_3_, 400 MHz) δ 1.34 (3 H, d, J = 6.5 Hz, H-7), 3.93–4.00 (2 H, m, H-2), 4.59 (2 H, d, J = 5.5 Hz, H-4), 5.17 (1 H, d, J = 10.5 Hz, H-10), 5.19–5.25 (1 H, m, NH), 5.22 (1 H, dd, J = 10.5, 1.2 Hz, H-6), 5.26 (1 H, d, J = 17.2 Hz, H-10’), 5.31 (1 H, dd, J = 17.2, 1.2 Hz, H-6’), 5.41 (1 H, dq, J = 6.4, 6.4 Hz, H-8), 5.83 (1 H, ddd, J = 17.3, 10.7, 6.3 Hz, H-9), 5.92 (1 H, ddt, J = 17.2, 10.4, 5.3 Hz, H-5); ^13^C-NMR (CDCl_3_, 100 MHz) δ 19.9 (q, C-7), 42.9 (t, C-2), 65.9 (t, C-4), 72.5 (d, C-8), 116.5 (t, C-10), 117.8 (t, C-6), 132.6 (d, C-5), 137.0 (d, C-9), 156.1 (s, C-3), 169.3 (s, C-1); HRMS (CI) *m*/*z* calculated for C_10_H_16_NO_4_ [M + H]^+^ 214.1074, found 214.1083.

#### 4.1.12. Synthesis of (*S*,*E*)-2-{[(allyloxy)carbonyl]amino}hex-4-enoic Acid (**16**)

Preparation of LDA solution: Under N_2_ atmosphere, *n*-BuLi (ρ = 0.680 g/mL, 22.6 mL, 36.2 mmol, 1.6 M in hexane) was added to a solution of diisopropylamine (ρ = 0.717 g/mL, 5.28 mL, 37.4 mmol) in 40 mL dry THF at –15 °C. Afterwards, the reaction mixture was stirred at this temperature for 15 min. Preparation of substrate solution: ZnCl_2_ (1.97 g, 14.5 mmol) was first dried in high vacuum at approx. 250 °C and then dissolved in 40 mL anhydrous THF. At room temperature the allyl ester **15** (2.57 g, 12.1 mmol) was added, which was also dissolved in 40 mL dry THF. Reaction procedure: Both reaction solutions were cooled to –78 °C. Subsequently, the LDA solution was slowly added to the substrate solution via cannula. Under stirring, the reaction was allowed to warm up to room temperature overnight. After complete conversion (15 h, TLC), the reaction mixture was hydrolyzed by 120 mL of 1 M aq. KHSO_4_ solution and then diluted with EtOAc. The aqueous phase was extracted three times with EtOAc and the combined organic phases were washed with brine and dried over Na_2_SO_4_. The solvent was removed under reduced pressure and the crude product was purified by flash chromatography (silica gel, DCM/MeOH 97:3 → 95:5) to afford crotyl-glycine **16** (2.23 g, 10.5 mmol, 87%) as a yellow resin. R_f_ = 0.36 (DCM/MeOH 95:5); [α]_D_^20^ = +14.3 (c = 1.0, CHCl_3_); Major rotamer: ^1^H-NMR (CDCl_3_, 400 MHz) δ 1.68 (3 H, d, J = 6.2 Hz, H-7), 2.42–2.60 (2 H, m, H-10), 4.40 (1 H, dt, J = 7.0, 5.7 Hz, H-2), 4.59 (2 H, d, J = 5.3 Hz, H-4), 5.18–5.27 (1 H, m, NH), 5.23 (1 H, dd, J = 10.4, 1.0 Hz, H-6), 5.27–5.39 (1 H, m, H-9), 5.31 (1 H, d, J = 16.8 Hz, H-6’), 5.60 (1 H, dq, J = 15.0, 6.5 Hz, H-8), 5.92 (1 H, ddt, J = 17.1, 10.5, 5.4 Hz, H-5), 5.97 (1 H, bs, COOH); Minor rotamer (selected signals): ^1^H-NMR (CDCl_3_, 400 MHz) δ 4.29 (1 H, bs, H-2); ^13^C-NMR (CDCl_3_, 100 MHz) δ 18.0 (q, C-7), 35.1 (t, C-10), 53.3 (d, C-2), 66.0 (t, C-4), 118.0 (t, C-6), 124.1 (d, C-9), 130.7 (d, C-8), 132.5 (d, C-5), 155.9 (s, C-3), 176.3 (s, C-1); HRMS (CI) *m*/*z* calculated for C_10_H_16_NO_4_ [M + H]^+^ 214.1074, found 214.1081.

### 4.2. Synthesis of the Linear Heptapeptide

#### 4.2.1. Synthesis of Methyl {[2*S*,4*S*]-2-({[benzyloxy]carbonyl}(methyl)amino)-5-[(tert-butyldimethyl-silyl)oxy]-4-methylpentanoyl}-L-leucinate (**17**)

To a solution of H-Leu-OMe·HCl (143 mg, 790 μmol) in 7.8 mL anhydrous DCM was added hydroxyleucine **12** (355 mg, 880 μmol) at room temperature. The reaction mixture was cooled to 0 °C, followed by successive addition of NMM (ρ = 0.91 g/mL, 295 mL, 2.68 mmol), HOBt (133 mg, 880 μmol) and EDC·HCl (166 mg, 880 μmol). Overnight, the solution was allowed to warm to room temperature (17 h). The reaction mixture was diluted with EtOAc. The organic phase was washed with 1 M aq. KHSO_4_ solution, sat. aq. NaHCO_3_ solution and brine, dried over Na_2_SO_4_ and concentrated under reduced pressure. After purification by flash chromatography (silica gel, cyclohexane → cyclohexane/EtOAc 8:2), 372 mg (690 μmol, 88%) of the dipeptide **17** was obtained as a colorless resin. R_f_ = 0.35 (PE/EtOAc 7:3); [α]_D_^20^ = –53.4 (c = 1.0, CHCl_3_); Major rotamer: ^1^H-NMR (CDCl_3_, 400 MHz) δ -0.01–0.06 (6 H, m, H-8), 0.73–0.98 (9 H, m, H-11, H-21), 0.88 (9 H, s, H-10), 1.44–1.53 (2 H, m, H-5, H-6), 1.49–1.58 (1 H, m, H-20), 1.49–1.66 (2 H, m, H-19), 1.92–2.10 (1 H, m, H-5’), 2.84 (3 H, s, H-12), 3.28–3.41 (1 H, m, H-7), 3.41–3.50 (1 H, m, H-7’), 3.71 (3 H, s, H-22), 4.51–4.59 (1 H, m, H-2), 4.79–4.88 (1 H, m, H-4), 5.09–5.26 (2 H, m, H-14), 6.38 (1 H, d, J = 7.5 Hz, NH), 7.29–7.40 (5 H, m, H-16, H-17, H-18); Minor rotamer (selected signals): ^1^H-NMR (CDCl_3_, 400 MHz) δ 0.89 (9 H, s, H-10), 4.70–4.79 (1 H, m, H-4), 6.08 (1 H, d, J = 7.1 Hz, NH); ^13^C-NMR (CDCl_3_, 100 MHz) δ -5.5 (q, C-8), 16.1 (q, C-11), 18.3 (s, C-9), 21.8 (q, C-21), 22.8 (q, C-21’), 24.9 (d, C-20), 25.9 (q, C-10), 29.4 (q, C-12), 30.8 (t, C-5), 32.4 (d, C-6), 41.2 (t, C-19), 50.6 (d, C-2), 52.3 (q, C-22), 56.3 (d, C-4), 67.6 (t, C-14), 68.3 (t, C-7), 127.8 (d, C-16), 128.1 (d, C-18), 128.5 (d, C-17), 136.4 (s, C-15), 157.4 (s, C-13), 170.8 (s, C-3), 173.1 (s, C-1); HRMS (CI) *m*/*z* calculated for C_28_H_49_N_2_O_6_Si [M + H]^+^ 537.3354, found 537.3355.

#### 4.2.2. Synthesis of Methyl {[2*S*,4*S*]-2-({*S*}-2-{[(allyloxy)carbonyl]amino}-*N*-methylpropan amido)-5-[(tert-butyldimethylsilyl)oxy]-4-methylpentanoyl}-L-leucinate (**18**)

To a solution of dipeptide **17** (807 mg, 1.50 mmol) in 15 mL MeOH was added 10 wt-% Pd-C (80.7 mg, 10% on activated charcoal). Until complete conversion (3 h, TLC), the reaction mixture was stirred under 1 atm H_2_ atmosphere at room temperature. The reaction solution was filtered through Celite and the solvent was removed under reduced pressure. The residue was dissolved in 30 mL dry DCM. After addition of Alloc-AlaOH (286 mg, 1.65 mmol) the reaction mixture was cooled to −20 °C, followed by addition of NMM (ρ = 0.91 g/mL, 0.36 mL, 3.3 mmol) and BEP (453 mg, 1.65 mmol). Overnight, it was slowly warmed to room temperature. After 18 h (TLC), the reaction mixture was diluted with DCM. The organic phase was washed with water, sat. aq. NaHCO_3_ solution as well as with brine, dried over Na_2_SO_4_ and concentrated under reduced pressure. Tripeptide **18** (654 mg, 1.17 mmol, 78%) was obtained as a colorless resin after purification by flash chromatography (silica gel, PE/EtOAc 7:3). R_f_ = 0.35 (PE/EtOAc 6:4); [α]_D_^20^ = −90.3 (c = 1.0, CHCl_3_); Major rotamer: ^1^H-NMR (CDCl_3_, 400 MHz) δ 0.03 (6 H, s, H-16), 0.85 (3 H, d, J = 6.2 Hz, H-19), 0.86–0.96 (6 H, m, H-22), 0.87 (9 H, s, H-18), 1.31 (3 H, d, J = 6.7 Hz, H-7), 1.43–1.53 (1 H, m, H-14), 1.48–1.59 (3 H, m, H-13, H-20, H-21), 1.57–1.70 (1 H, m, H-20’), 2.00–2.09 (1 H, m, H-13’), 2.96 (3 H, s, H-12), 3.31-3.40 (1 H, m, H-15), 3.47 (1 H, dd, J = 9.7, 4.8 Hz, H-15’), 3.71 (3 H, s, H-23), 4.50–4.59 (1 H, m, H-2), 4.56 (2 H, d, J = 5.3 Hz, H-9), 4.65 (1 H, dq, J = 7.1, 7.1 Hz, H-6), 5.14–5.22 (1 H, m, H-4), 5.21 (1 H, dd, J = 10.4, 1.2 Hz, H-11), 5.30 (1 H, dd, J = 17.2, 1.5 Hz, H-11’), 5.71 (1 H, d, J = 7.8 Hz, NH_b_), 5.91 (1 H, ddt, J = 17.4, 10.5, 5.3 Hz, H-10), 6.36 (1 H, d, J = 8.1 Hz, NH_a_); Minor rotamer (selected signals): ^1^H-NMR (CDCl_3_, 400 MHz) δ 0.87 (9 H, s, H-18), 1.35 (3 H, d, J = 6.9 Hz, H-7), 2.77 (3 H, s, H-12), 3.52 (1 H, dd, J = 9.9, 4.9 Hz, H-15’), 3.71 (3 H, s, H-23), 4.43–4.50 (3 H, m, H-2, H-9), 4.68–4.74 (1 H, m, H-6), 4.74 (1 H, dd, J = 8.1, 6.8 Hz, H-4) 5.24–5.31 (1 H, m, H-11’), 5.36 (1 H, d, J = 7.0 Hz, NH_b_), 5.80–5.90 (1 H, m, H-10), 7.88 (1 H, d, J = 7.6 Hz, NH_a_); Major rotamer: ^13^C-NMR (CDCl_3_, 100 MHz) δ -5.5 (q, C-16), 16.2 (q, C-19), 18.3 (s, C-17), 18.5 (q, C-7), 21.6 (q, C-22), 22.8 (q, C-22’), 24.8 (d, C-21), 25.9 (q, C-18), 30.1 (q, C-12), 30.6 (t, C-13), 32.3 (d, C-14), 41.3 (t, C-20), 47.2 (d, C-6), 50.5 (d, C-2), 52.3 (q, C-23), 54.1 (d, C-4), 65.6 (t, C-9), 68.2 (t, C-15), 117.7 (t, C-11), 132.7 (d, C-10), 155.3 (s, C-8), 170.2 (s, C-3), 173.0 (s, C-1), 173.5 (s, C-5); Minor rotamer (selected signals): ^13^C-NMR (CDCl_3_, 100 MHz) δ 16.9 (q, C-19), 18.0 (q, C-7), 21.2 (q, C-22), 23.0 (q, C-22’), 24.7 (d, C-21), 28.9 (q, C-12), 32.1 (d, C-14), 32.3 (t, C-13), 39.9 (t, C-20), 45.8 (d, C-6), 51.3 (d, C-2), 52.1 (q, C-23), 58.1 (d, C-4), 66.1 (t, C-9), 68.1 (t, C-15), 118.1 (t, C-11), 132.2 (d, C-10), 156.5 (s, C-8), 169.7 (s, C-3), 173.2 (s, C-1), 173.4 (s, C-5); HRMS (CI) *m*/*z* calculated for C_27_H_52_N_3_O_7_Si [M + H]^+^ 558.3569, found 558.3586.

#### 4.2.3. Synthesis of Methyl {[2*S*,4*S*]-2-({*S*}-2-[(*S*)-2-{[(allyloxy)carbonyl]amino}-3-{4-[(2-methoxy-ethoxy)methoxy]-3-nitrophenyl}propanamido]-*N*-methylpropanamido)-5-[(tert-butyldimethylsilyl)oxy]-4-methylpentanoyl}-L-leucinate (**19**)

Tripeptide **18** (600 mg, 1.08 mmol) was reacted according to the general procedure for allyl deprotection with diethylamine (ρ = 0.707 g/mL, 0.56 mL, 5.4 mmol), TPPTS (24 mg, 43 μmol) and Pd(OAc)_2_ (1.1 mL, 22 μmol, 0.02 M in MeCN) in 10.8 mL of MeCN and water for 3 h. The solvent was removed in vacuo and the residue was dissolved in 10.8 mL anhydrous DCM. Afterwards nitrotyrosine **5** (472 mg, 1.18 mmol) was added at room temperature. The reaction mixture was cooled to 0 °C, DIPEA (ρ = 0.742 g/mL, 400 μL, 2.26 mmol) was directly added and after 10 min TBTU (380 mg, 1.18 mmol). Overnight, the reaction solution was slowly warmed to room temperature. After complete conversion (19 h, LCMS), the solvent was removed in vacuo and the crude product was dissolved in EtOAc. The organic phase was successively washed with 1 M aq. KHSO_4_ solution, sat. aq. NaHCO_3_ solution and brine and dried over Na_2_SO_4_. The solvent was removed under reduced pressure. Purification by reverse phase flash chromatography (C18 silica gel, H_2_O/MeCN 9:1 → 5:95) afforded tetrapeptide **19** (637 mg, 750 μmol, 69%) as a yellow resin. [α]_D_^20^ = −51.3 (c = 0.75, CHCl_3_); Major rotamer: ^1^H-NMR (CDCl_3_, 400 MHz) δ 0.04 (6 H, s, H-29), 0.84 (3 H, d, J = 6.1 Hz, H-32), 0.85–0.96 (6 H, m, H-35), 0.88 (9 H, s, H-31), 1.30 (3 H, d, J = 6.7 Hz, H-24), 1.42–1.57 (4 H, m, H-26, H-27, H-33, H-34), 1.57–1.65 (1 H, m, H-33’), 2.01–2.12 (1 H, m, H-26’), 2.96–3.06 (1 H, m, H-13), 3.00 (3 H, s, H-25), 3.10 (1 H, dd, J = 14.1, 5.7 Hz, H-13’), 3.32–3.40 (1 H, m, H-28), 3.36 (3 H, s, H-23), 3.46–3.54 (1 H, m, H-28’), 3.54–3.58 (2 H, m, H-22), 3.72 (3 H, s, H-36), 3.84–3.89 (2 H, m, H-21), 4.38–4.59 (4 H, m, H-2, H-8, H-10), 4.76–4.91 (1 H, m, H-6), 5.17 (1 H, dd, J = 10.3, 5.5 Hz, H-4), 5.20 (1 H, dd, J = 10.4, 1.2 Hz, H-12), 5.26 (1 H, d, J = 17.2 Hz, H-12’), 5.31–5.36 (1 H, m, NH_c_), 5.35 (2 H, s, H20), 5.86 (1 H, ddt, J = 17.2, 10.7, 5.5 Hz, H-11), 6.46 (1 H, d, J = 8.1 Hz, NH_a_), 7.04–7.13 (1 H, m, NH_b_), 7.25–7.30 (1 H, m, H-18), 7.33 (1 H, dd, J = 8.6, 2.2 Hz, H-19), 7.60 (1 H, d, J = 1.2 Hz, H-15); Minor rotamer (selected signals): ^1^H-NMR (CDCl_3_, 400 MHz) δ 0.03 (6 H, s, H-29), 0.87 (9 H, s, H-31), 1.36 (3 H, d, J = 6.7 Hz, H-24), 3.71 (3 H, s, H-36), 6.65 (1 H, d, J = 5.9 Hz, NH_b_), 7.34–7.39 (1 H, m, H-19), 7.67 (1 H, d, J = 2.1 Hz, H-15), 7.92 (1 H, d, J = 7.5 Hz, NH_a_); ^13^C-NMR (CDCl_3_, 100 MHz) δ -5.5 (q, C-29), 16.2 (q, C-32), 18.1 (q, C-24), 18.3 (s, C-30), 21.7 (q, C-35), 22.8 (q, C-35’), 24.8 (d, C-34), 25.9 (q, C-31), 30.3 (q, C-25), 31.1 (t, C-26), 32.4 (d, C-27), 37.4 (t, C-13), 41.3 (t, C-33), 46.1 (d, C-6), 50.5 (d, C-2), 52.3 (q, C-36), 54.3 (d, C-4), 59.0 (d, C-8, C-23), 66.0 (t, C-10), 68.2 (t, C-28), 68.4 (t, C-21), 71.4 (t, C-22), 94.3 (t, C-20), 117.5 (d, C-18), 118.1 (t, C-12), 126.0 (d, C-15), 130.1 (s, C-14), 132.3 (d, C-11), 134.8 (d, C-19), 140.4 (s, C-16), 149.4 (s, C-17), 155.6 (s, C-9), 169.3 (s, C-7), 170.3 (s, C-3), 172.9 (s, C-5), 173.0 (s, C-1); HRMS (ESI) *m*/*z* calculated for C_40_H_68_N_5_O_13_Si [M + H]^+^ 854.4577, found 854.4577.

#### 4.2.4. Synthesis of Methyl {[2*S*,4*S*]-2-({*S*}-2-[(*S*)-2-{[*S*]-2-({[allyloxy]carbonyl}(methyl)amino)-4-methylpentanamido}-3-{4-[(2-methoxyethoxy)methoxy]-3-nitrophenyl}-propan-amido]-*N*-methylpropanamido)-5-[(*tert*-butyldimethylsilyl)oxy]-4-methyl-pentanoyl}-L-leucinate (**20**)

Tetrapeptide **19** (494 mg, 580 μmol) was reacted according to the general procedure for allyl deprotection with diethylamine (ρ = 0.707 g/mL, 0.30 mL, 2.9 mmol), TPPTS (13.2 mg, 23.0 μmol) and Pd(OAc)_2_ (580 μL, 12 μmol, 0.02 M in MeCN) in 5.8 mL of MeCN and water for 3 h. After removal of the solvent under reduced pressure, the residue was dissolved in 5.8 mL anhydrous DCM. To the reaction mixture was added Alloc-*N*-Me-LeuOH (177 mg, 690 μmol) and it was cooled to 0 °C. DIPEA (ρ = 0.742 g/mL, 313 µL, 1.79 mmol) was directly added and after 10 min PyBOP (361 mg, 690 μmol). Overnight, the reaction mixture was slowly warmed to room temperature. After 17 h (LCMS), the solvent was removed in vacuo and the crude product was dissolved in EtOAc. The organic phase was washed with 1 M aq. KHSO_4_ solution, sat. aq. NaHCO_3_ solution and brine, dried over Na_2_SO_4_ and concentrated. Pentapeptide **20** (434 mg, 440 μmol, 77%) was obtained as a yellow foam after purification by reverse phase flash chromatography (C18 silica gel, H_2_O/MeCN 9:1 → 5:95). [α]_D_^20^ = −70.1 (c = 1.0, CHCl_3_); Major rotamer: ^1^H-NMR (CDCl_3_, 400 MHz) δ 0.04 (6 H, s, H-35), 0.84 (3 H, d, J = 6.0 Hz, H-38), 0.85-0.95 (12 H, m, H-18, H-41), 0.87 (9 H, s, H-37), 1.29 (3 H, d, J = 6.7 Hz, H-30), 1.40–1.56 (2 H, m, H-17, H-40), 1.46–1.56 (3 H, m, H-32, H-33, H39), 1.56–1.71 (3 H, m, H-16, H-39’), 2.01–2.12 (1 H, m, H-32’), 2.66 (3 H, s, H-15), 2.95 (1 H, dd, J = 14.3, 7.5 Hz, H-19), 2.98 (3 H, s, H-31), 3.09–3.19 (1 H, m, H-19’), 3.32–3.40 (1 H, m, H-34), 3.36 (3 H, s, H-29), 3.49 (1 H, dd, J = 10.1, 4.4 Hz, H-34’), 3.52–3.57 (2 H, m, H-28), 3.72 (3 H, s, H-42), 3.82–3.88 (2 H, m, H-27), 4.44–4.57 (1 H, m, H-2), 4.50–4.65 (3 H, m, H-10, H-12), 4.65–4.73 (1 H, m, H-8), 4.81 (1 H, dq, J = 7.0, 7.0 Hz, H-6), 5.15 (1 H, dd, J = 10.0, 5.3 Hz, H-4), 5.22 (1 H, d, J = 10.5 Hz, H-14), 5.30 (1 H, d, J = 17.9 Hz, H-14’), 5.34 (2 H, s, H-26), 5.83–6.01 (1 H, m, H-13), 6.39 (1 H, d, J = 7.7 Hz, NH_a_), 6.64 (1 H, d, J = 7.2 Hz, NH_c_), 7.00–7.08 (1 H, m, NH_b_), 7.21–7.30 (1 H, m, H-24), 7.28–7.37 (1 H, m, H-25), 7.56 (1 H, s, H-21); Minor rotamer (selected signals): ^1^H-NMR (CDCl_3_, 400 MHz) δ 0.02 (6 H, s, H-35), 0.87 (9 H, s, H-37), 0.89–0.92 (3 H, m, H-38), 1.35 (3 H, d, J = 6.9 Hz, H-30), 3.71 (3 H, s, H-42), 7.62 (1 H, bs, H-21); Major rotamer: ^13^C-NMR (CDCl_3_, 100 MHz) δ -5.5 (q; C-35), 16.2 (q, C-38), 18.0 (q, C-30), 18.3 (s, C-36), 21.7 (q, C-18, C-41), 22.8 (q, C-18’, C-41’), 24.8 (d, C-17, C-40), 25.9 (q, C-37), 29.6 (q; C-15), 30.2 (q, C-31), 30.8 (t, C-32), 32.3 (d, C-33), 36.3 (t; C-16), 36.8 (t, C-19), 41.3 (t, C-39), 46.1 (d, C-6), 50.5 (d, C-2), 52.3 (q, C-42), 53.4 (d, C-8), 54.2 (d, C-4), 56.9 (d, C-10), 59.0 (q, C-29), 66.6 (t, C-12), 68.2 (t, C-34), 68.4 (t, C-27), 71.3 (t, C-28), 94.3 (t, C-26), 117.5 (d, C-24), 117.6 (t, C-14), 125.9 (d, C-21), 130.3 (s, C-20), 132.6 (d, C-13), 134.6 (d, C-25), 140.4 (s, C-22), 149.3 (s, C-23), 157.1 (s, C-11), 169.0 (s, C-7, C-9), 170.2 (s, C-3), 172.8 (s, C-5), 173.0 (s, C-1); Minor rotamer (selected signals): ^13^C-NMR (CDCl_3_, 100 MHz) δ -5.5 (q; C-35), 16.9 (q, C-38), 17.6 (q, C-30); HRMS (ESI) *m*/*z* calculated for C_47_H_81_N_6_O_14_Si [M + H]^+^ 981.5575, found 981.5574.

#### 4.2.5. Synthesis of Methyl {[2*S*,4*S*]-2-({*S*}-2-[(*S*)-2-{[*S*]-2-({*S*}-2-[({allyloxy}carbonyl)amino]-N-methyl-3-[1-methyl-1H-indol-3-yl]propanamido)-4-methylpentanamido}-3-(4-{[2-methoxyethoxy]methoxy}-3-nitrophenyl)propanamido]-N-methylpropanamido)-5-[(tert-butyldimethylsilyl)oxy]-4-methylpentanoyl}-L-leucinate (**21**)

According to the general procedure for allyl deprotection, pentapeptide **20** (30 mg, 31 μmol) was reacted with diethylamine (ρ = 0.707 g/mL, 16.0 μL, 0.15 mmol), TPPTS (0.7 mg, 1.2 μmol) and Pd(OAc)_2_ (31 μL, 0.61 μmol, 0.02 M in MeCN) in 0.3 mL of MeCN and water for 3 h. After the solvent was removed in vacuo, the residue was dissolved in 0.3 mL dry DMF. Alloc-*N’*-Me-TrpOH (18.5 mg, 61.0 μmol), HATU (23.2 mg, 61.0 μmol), HOAt (4.2 mg, 31.0 μmol) and DIPEA (ρ = 0.742 g/mL, 27 µL, 0.15 mmol) were added successively to the reaction mixture at room temperature. After complete conversion (16 h, LCMS), the reaction solution was diluted with EtOAc. The organic phase was washed once with 1 M aq. KHSO_4_, three times with water and once with sat. aq. NaHCO_3_ solution and brine, dried over Na_2_SO_4_ and concentrated under reduced pressure. Hexapeptide **21** (28 mg, 24 μmol, 78%) could be isolated as a yellow foam after purification by reverse phase flash chromatography (C18 silica gel, H_2_O/MeCN 9:1 → 5:95). [α]_D_^20^ = −28.3 (c = 1.0, CHCl_3_); Major rotamer: ^1^H-NMR (CDCl_3_, 400 MHz) δ 0.03 (6 H, s, H-47), 0.70–0.93 (12 H, m, H-30, H-53), 0.79–0.86 (3 H, m, H-50), 0.88 (9 H, s, H-49), 1.31 (3 H, d, J = 6.7 Hz, H-42), 1.42–1.71 (8 H, m, H-28, H-29, H-44, H-45, H-51, H-52), 2.00–2.12 (1 H, m, H-44’), 2.41 (3 H, s, H-27), 2.65–3.32 (4 H, m, H-17, H-31), 2.98 (3 H, s, H43), 3.31-3.40 (1 H, m, H-46), 3.33 (3 H, s, H-41), 3.42–3.55 (1 H, m, H-46’), 3.46–3.50 (2 H, m, H-40), 3.67–3.75 (6 H, m, H-26, H-54), 3.76–3.81 (2 H, m, H-39), 4.38–4.75 (5 H, m, H-2, H-8, H-10, H-14), 4.75–5.00 (2 H, m, H-6, H-12), 5.10–5.22 (1 H, m, H-4), 5.12–5.31 (2 H, m, H-16), 5.22 (2 H, s, H-38), 5.78–5.95 (2 H, m, H-15, NH_d_), 6.27 (1 H, d, J = 8.1 Hz, NH_c_), 6.40 (1 H, d, J = 7.7 Hz, NH_a_), 6.88 (1 H, s, H-19), 7.10 (1 H, dd, J = 6.9 Hz, H-23), 7.16–7.29 (3 H, m, H-36, H-37, NH_b_), 7.18–7.26 (1 H, m, H-22), 7.23–7.31 (1 H, m, H-21), 7.30–7.35 (1 H, m, H-33), 7.63–7.69 (1 H, m, H-24); Minor rotamer (selected signals): ^1^H-NMR (CDCl_3_, 400 MHz) δ 0.02 (6 H, s, H-47), 0.45 (6 H, d, J = 6.6 Hz, H-30, H-53), 0.54 (6 H, d, J = 6.6 Hz, H-30’, H-53’), 0.86 (9 H, s, H-49), 0.97 (3 H, d, J = 6.6 Hz, H-50), 1.41 (3 H, d, J = 6.5 Hz, H-42), 2.36 (3 H, s, H-27), 2.93 (3 H, s, H43), 3.37 (3 H, s, H-41), 3.50–3.59 (2 H, m, H-40), 3.81–3.93 (2 H, m, H-39), 5.28–5.39 (2 H, m, H-38), 6.85 (1 H, s, H-19), 7.54 (1 H, d, J = 8.1 Hz, H-24), 7.58 (1 H, d, J = 2.1 Hz, H-33); Major rotamer: ^13^C-NMR (CDCl_3_, 100 MHz) δ -5.5 (q, C-47), 16.1 (q, C-50), 18.1 (q, C-42), 18.3 (s, C-48), 21.7 (q, C-30, C-53), 22.8 (q, C-30’, C-53’), 24.8 (d, C-29, C-52), 25.9 (q, C-49), 28.7 (t, C-17), 28.8 (q, C-27), 30.1 (q, C-43), 30.6 (t, C-44), 32.3 (d, C-45), 32.7 (q, C-26), 36.1 (t, C-31), 36.6 (t, C-28), 41.3 (t, C-51), 45.9 (d, C-6), 50.5 (d, C-2), 51.6 (d, C-12), 52.3 (q, C-54), 54.0 (d, C-8), 54.3 (d, C-4), 58.4 (d, C-10), 59.0 (q, C-41), 66.3 (t, C-14), 68.2 (t, C-46), 68.3 (t, C-39), 71.3 (t, C-40), 94.2 (t, C-38), 108.2 (s, C-18), 109.4 (d, C-21), 117.5 (d, C-36), 117.8 (t, C-16), 118.3 (d, C-24), 119.4 (d, C-23), 122.0 (d, C-22), 125.8 (d, C-33), 127.6 (d, C-19), 127.9 (s, C-25), 130.9 (s, C-32), 132.7 (d, C-15), 134.6 (d, C-37), 136.9 (s, C-20), 140.4 (s, C-34), 149.1 (s, C-35), 156.9 (s, C-13), 169.0 (s, C-9), 169.3 (s, C-7), 170.1 (s, C-3), 173.0 (s, C-5), 173.0 (s, C-1), 173.3 (s, C-11); Minor rotamer (selected signals): ^13^C-NMR (CDCl_3_, 100 MHz) δ -5.5 (q, C-47), 16.7 (q, C-50), 17.5 (q, C-42), 21.7 (q, C-30, C-53), 23.0 (q, C-30’, C-53’), 24.6 (d, C-29, C-52), 36.4 (t, C-28), 41.2 (t, C-51), 45.5 (d, C-6), 50.5 (d, C-2), 58.9 (q, C-41), 71.4 (t, C-40), 94.5 (t, C-38); HRMS (ESI) *m*/*z* calculated for C_59_H_93_N_8_O_15_Si [M + H]^+^ 1181.6524, found 1181.6522.

#### 4.2.6. Synthesis of Methyl {[2*S*,4*S*]-2-({*S*}-2-[(*S*)-2-{[*S*]-2-({*S*}-2-[(S,E)-2-{[(allyloxy)carbonyl]amino}-hex-4-enamido]-N-methyl-3-{1-methyl-1H-indol-3-yl}propanamido)-4-methyl-pentanamido}-3-[4-({2-methoxyethoxy}methoxy)-3-nitrophenyl]propanamido]-N-methylpropanamido)-5-[(tert-butyldimethylsilyl)oxy]-4-methylpentanoyl}-L-leucinate (**22**)

To a solution of hexapeptide **21** (226 mg, 190 μmol) in 1.9 mL dry DCM was added 1,3-dimethylbarbituric acid (90 mg, 0.57 mmol) and Pd(Ph_3_P)_4_ (6.6 mg, 5.7 µmol) at room temperature. After 45 min the reaction mixture was diluted with EtOAc and the organic phase was washed three times with sat. aq. NaHCO_3_ solution. Subsequently, a back extraction from the aqueous phase was carried out with EtOAc. The combined organic phases were dried over Na_2_SO_4_ and concentrated under reduced pressure. The residue was dissolved again in 1.9 mL dry DCM and Alloc-crotylglycine **16** (44.9 mg, 210 μmol) was added. The reaction mixture was cooled to 0 °C, treated with NMM (ρ = 0.91 g/mL, 51 µL, 0.46 mmol), HOBt (32.2 mg, 210 μmol) and EDC·HCl (40.4 mg, 210 μmol) and warmed to room temperature overnight. After 18 h (LCMS), the solution was diluted with EtOAc. The organic phase was washed with 1 M aq. KHSO_4_ solution, sat. aq. NaHCO_3_ solution and brine, dried over Na_2_SO_4_ and concentrated. Heptapeptide **22** (215 mg, 170 μmol, 87%) was obtained as a yellow foam after purification by reverse phase flash chromatography (C18 silica gel, H_2_O/MeCN 9:1 → 5:95). [α]_D_^20^ = −76.8 (c = 0.75, CHCl_3_); Major rotamer: ^1^H-NMR (CDCl_3_, 500 MHz) δ 0.04 (6 H, s, H-53), 0.73–0.82 (6 H, m, H-36), 0.82–0.87 (3 H, m, H-56), 0.82–1.01 (6 H, m, H-59), 0.89 (9 H, s, H-55), 1.08–1.22 (1 H, m, H-35), 1.39 (3 H, d, J = 6.7 Hz, H-48), 1.43–1.62 (6 H, m, H-34, H-50, H-51, H-57, H-58), 1.61 (3 H, d, J = 5.3 Hz, H-22), 1.66–1.76 (1 H, m, H-34’), 2.11–2.19 (1 H, m, H-50’), 2.25–2.38 (1 H, m, H-19), 2.38–2.48 (1 H, m, H-19’), 2.81 (3 H, s, H-33), 2.91-2.99 (1 H, m, H-37), 3.04–3.11 (1 H, m, H-37’), 3.10–3.20 (2 H, m, H-23), 3.12 (3 H, s, H-49), 3.32–3.40 (1 H, m, H-52), 3.34 (3 H, s, H-47), 3.43–3.53 (1 H, m, H-52’), 3.48–3.58 (2 H, m, H-46), 3.68–3.75 (6 H, m, H-32, H-60), 3.79–3.89 (2 H, m, H-45), 4.31–4.42 (1 H, m, H-14), 4.49–4.57 (2 H, m, H-2, H-10), 4.50–4.59 (2 H, m, H-16), 4.54–4.64 (1 H, m, H-8), 4.92–5.02 (1 H, m, H-6), 5.17–5.28 (3 H, m, H-4, H-12, H-18), 5.25–5.38 (4 H, m, H-18’, H-20, H-44), 5.52 (1 H, dq, J = 8.4, 6.8 Hz, H-21), 5.66 (1 H, bs, NH_e_), 5.90 (1 H, ddt, J = 16.9, 10.5, 5.3 Hz, H-17), 6.27 (1 H, d, J = 8.4 Hz, NH_c_), 6.83 (1 H, s, H-25), 6.95 (1 H, bs, NH_a_), 7.11 (1 H, dd, J = 7.0, 7.0 Hz, H-29), 7.17–7.24 (1 H, m, H-28), 7.20–7.34 (3 H, m, H-27, H-42, H-43), 7.45 (1 H, bs, H-39), 7.49 (1 H, bs, NH_b_), 7.68 (1 H, d, J = 7.9 Hz, H-30), 7.68–7.72 (1 H, m, NH_d_); Minor rotamer (selected signals): ^1^H-NMR (CDCl_3_, 500 MHz) δ 0.01–0.08 (6 H, m, H-53), 0.74–0.81 (3 H, m, H-56), 0.87 (9 H, s, H-55), 1.27 (3 H, d, J = 6.9 Hz, H-48), 1.64 (3 H, d, J = 6.0 Hz, H-22), 2.02–2.10 (1 H, m, H-50’), 2.76 (3 H, s, H-33), 2.92–2.96 (3 H, m, H-49), 2.96–3.02 (2 H, m, H-23), 3.36 (3 H, s, H-47), 4.81 (1 H, dq, J = 6.9, 6.9 Hz, H-6), 5.10–5.19 (2 H, m, H-4, H-12), 6.86 (1 H, s, H-25); Major rotamer: ^13^C-NMR (CDCl_3_, 125 MHz) δ -5.5 (q, C-53), 15.9 (q, C-56), 17.8 (q, C-48), 18.0 (q, C-22), 18.3 (s, C-54), 21.6 (q, C-59), 21.8 (q, C-36), 22.9 (q, C-59’), 23.0 (q, C-36’), 24.7 (d, C-35), 24.9 (d, C-58), 25.9 (q, C-55), 28.4 (t, C-23), 29.2 (q, C-33), 30.6 (q, C-49), 31.6 (t, C-50), 32.5 (d, C-51), 32.7 (q, C-32), 35.9 (t, C-37), 36.7 (t, C-19), 36.8 (t, C-34), 40.9 (t, C-57), 45.8 (d, C-6), 49.8 (d, C-12), 50.4 (d, C-2), 52.2 (q, C-60), 53.9 (d, C-8), 54.2 (d, C-4), 54.4 (d, C-14), 58.7 (d, C-10), 59.0 (q, C-47), 65.8 (t, C-16), 68.2 (t, C-52), 68.4 (t, C-45), 71.3 (t, C-46), 94.3 (t, C-44), 108.9 (s, C-24), 109.3 (d, C-27), 117.6 (d, C-42), 117.8 (t, C-18), 118.9 (d, C-30), 119.3 (d, C-29), 121.9 (d, C-28), 124.9 (d, C-20), 125.9 (d, C-39), 127.7 (d, C-25), 127.9 (s, C-31), 129.7 (d, C-21), 130.8 (s, C-38), 132.6 (d, C-17), 134.7 (d, C-43), 136.9 (s, C-26), 140.4 (s, C-40), 149.2 (s, C-41), 155.9 (s, C-15), 169.1 (s, C-7), 170.4 (s, C-9), 170.9 (s, C-3), 171.7 (s, C-13), 172.7 (s, C-11), 173.1 (s, C-1), 173.7 (s, C-5); Minor rotamer (selected signals): ^13^C-NMR (CDCl_3_, 125 MHz) δ 16.0 (q, C-56), 26.0 (q, C-55), 28.9 (q, C-33), 30.0 (q, C-49), 30.6 (t, C-50), 59.0 (q, C-47), 68.4 (t, C-45), 71.4 (t, C-46), 94.4 (t, C-44), 109.4 (d, C-27), 140.5 (s, C-40), 149.0 (s, C-41); HRMS (ESI) *m*/*z* calculated for C_65_H_102_N_9_O_16_Si [M + H]^+^ 1292.7208, found 1292.7222.

### 4.3. Synthesis of Cyclic Ilamycin Derivatives

#### 4.3.1. Synthesis of (3*S*,6*S*,9*S*,12*S*,15*S*,18*S*,21*S*)-15-[(*E*)-But-2-en-1-yl]-21-[(*S*)-3-{(*tert*-butyldimethyl-silyl)oxy}-2-methylpropyl]-9,18-diisobutyl-6-[4-({2-methoxyethoxy}methoxy)-3-nitrobenzyl]-1,3,10-trimethyl-12-[(1-methyl-1*H*-indol-3-yl)methyl]-1,4,7,10,13,16,19-heptaazacyclohenicosane-2,5,8,11,14,17,20-heptaone (**23**)

To a solution of linear heptapeptide **22** (181 mg, 140 μmol) in 1.4 mL THF was added a 1 M aq. LiOH solution (210 µL, 210 μmol) at 0 °C. Overnight the reaction mixture was slowly warmed to room temperature. After 15 h (LCMS), the solvent was removed under reduced pressure. According to the general procedure for allyl deprotection the residue was reacted with diethylamine (ρ = 0.707 g/mL, 73 µL, 0.70 mmol), TPPTS (3.2 mg, 5.6 µmol) and Pd(OAc)_2_ (140 µL, 2.8 µmol, 0.02 M in MeCN) in 1.4 mL of MeCN and water for 1 h. The reaction mixture was concentrated and the crude product was dried under high vacuum. HATU (186 mg, 490 μmol) and DIPEA (ρ = 0.717 g/mL, 110 μL, 630 μmol) were dissolved in 135 mL dry DCM in a three-neck flask at room temperature. Via syringe pump the heptapeptide, which was dissolved in 4.5 mL anhydrous DMF, was added dropwise over 4 h to the reaction solution. Stirring was performed overnight at room temperature. After complete conversion (16 h, LCMS), the solvent was removed in vacuo and the residue was dissolved in EtOAc. The organic phase was washed once with 1 M aq. KHSO_4_ solution, three times with water and once with sat. aq. NaHCO_3_ solution and brine, dried over Na_2_SO_4_ and concentrated under reduced pressure. The crude product was first purified by reverse phase flash chromatography (C18 silica gel, H_2_O/MeCN 9:1 → 5:95). Further purification by preparative HPLC (Luna, H_2_O/MeCN 7:3 → MeCN) yielded 23 (77 mg, 65 μmol, 47%) as a yellowish foam. [α]_D_^20^ = –75.9 (c = 1.0, CHCl_3_); ^1^H-NMR ((CD_3_)_2_SO, 400 MHz) δ −0.41-(-0.30) (1 H, m, H-30), −0.02 (3 H, s, H 49), -0.02 (3 H, s, H-49’), 0.22 (3 H, d, J = 6.6 Hz, H-32), 0.44 (3 H, d, J = 6.6 Hz, H-32’), 0.77–0.86 (6 H, m, H-55), 0.81 (9 H, s, H-51), 0.81–0.92 (3 H, m, H-52), 0.94-1.07 (1 H, m, H-31), 1.13 (3 H, d, J = 6.7 Hz, H-44), 1.36–1.48 (1 H, m, H-47), 1.45–1.60 (4 H, m, H-30’, H-53, H-54), 1.46 (3 H, d, J = 6.2 Hz, H-18), 1.61–1.78 (2 H, m, H-46), 2.34–2.45 (1 H, m, H-15), 2.50 (3 H, s, H-29), 2.50–2.61 (1 H, m, H-15’), 2.53–2.65 (1 H, m, H-33), 2.59 (3 H, s, H-45), 2.96 (1 H, dd, J = 13.0, 9.6 Hz, H-33’), 3.04 (1 H, dd, J = 13.1, 4.1 Hz, H-19), 3.20 (3 H, s, H-43), 3.29 (1 H, dd, J = 13.5, 9.6 Hz, H-19’), 3.30–3.39 (1 H, m, H-48), 3.44 (2 H, t, J = 4.7 Hz, H-42), 3.48 (1 H, dd, J = 10.0, 4.8 Hz, H-48’), 3.69–3.76 (2 H, m, H-41), 3.71 (3 H, s, H-28), 4.22–4.35 (1 H, m, H-2), 4.39 (1 H, dt, J = 6.0, 5.0 Hz, H-14), 4.46–4.56 (2 H, m, H-8, H-10), 4.67–4.84 (2 H, m, H-6, H-12), 4.95 (1 H, t, J = 7.1 Hz, H-4), 5.03 (1 H, ddd, J = 15.3, 7.5, 7.5 Hz, H-16), 5.26–5.37 (1 H, m, H-17), 5.36 (2 H, s, H-40), 6.99 (1 H, dd, J = 7.5, 7.5 Hz, H-25), 7.05 (1 H, s, H-21), 7.13 (1 H, dd, J = 7.5, 7.5 Hz, H-24), 7.24–7.33 (2 H, m, H-38, H-39), 7.38 (1 H, d, J = 8.4 Hz, H-23), 7.44 (1 H, d, J = 1.6 Hz, H-35), 7.47 (1 H, d, J = 7.8 Hz, H-26), 7.48 (1 H, d, J = 6.2 Hz, NH_e_), 8.61 (1 H, d, J = 9.2 Hz, NH_a_), 8.74 (1 H, d, J = 8.0 Hz, NH_c_), 9.16 (1 H, d, J = 3.9 Hz, NH_b_), 9.30 (1 H, d, J = 6.2 Hz, NH_d_); ^13^C-NMR ((CD_3_)_2_SO, 100 MHz) δ -5.6 (q, C-49), -5.5 (q, C-49‘), 16.6 (q, C-44), 16.9 (q, C-52), 18.0 (q, C-18), 18.0 (s, C-50), 20.7 (q, C-32), 21.3 (q, C-55), 22.7 (q, C-55’), 22.9 (q, C-32’), 23.6 (d, C-31), 24.1 (d, C-54), 25.7 (q, C-51), 26.9 (t, C-19), 28.1 (q, C-45), 28.6 (q, C-29), 31.8 (d, C-47), 32.3 (q, C-28), 33.0 (t, C-46), 34.5 (t, C-15), 36.8 (t, C-30, C-33), 42.4 (t, C-53), 45.0 (d, C-6), 50.2 (d, C-12), 51.3 (d, C-14), 53.0 (d, C-2), 53.1 (d, C-8), 57.2 (d, C-4), 57.6 (d, C-10), 58.0 (q, C-43), 68.0 (t, C-41), 68.2 (t, C-48), 70.9 (t, C-42), 93.9 (t, C-40), 108.5 (s, C-20), 109.7 (d, C-23), 117.2 (d, C-38), 118.4 (d, C-26), 118.6 (d, C-25), 121.3 (d, C-24), 124.7 (d, C-16), 125.4 (d, C-35), 127.3 (s, C-27), 128.1 (d, C-21), 128.7 (d, C-17), 130.1 (s, C-34), 134.7 (d, C-39), 136.5 (s, C-22), 140.1 (s, C-36), 148.2 (s, C-37), 167.3 (s, C-9), 169.1 (s, C-3), 170.4 (s, C-7), 170.7 (s, C-1), 171.2 (s, C-13), 171.4 (s, C-11), 172.3 (s, C-5); HRMS (ESI) *m*/*z* calculated for C_60_H_94_N_9_O_13_Si [M + H]^+^ 1176.6735, found 1176.6732.

#### 4.3.2. Synthesis of (3*S*,6*S*,9*S*,12*S*,15*S*,18*S*,21*S*)-15-[(*E*)-But-2-en-1-yl]-21-[(*S*)-3-hydroxy-2-methyl-propyl]-6-(4-hydroxy-3-nitrobenzyl)-9,18-diisobutyl-1,3,10-trimethyl-12-[(1-methyl-1*H*-indol-3-yl)methyl]-1,4,7,10,13,16,19-heptaazacyclohenicosane-2,5,8,11,14,17,20-heptaone (**24**)

To a solution of protected cyclic heptapeptide **23** (77 mg, 65 μmol) in 6.5 mL dry DCM was added 1,2-ethanedithiol (ρ = 1.12 g/mL, 165 mL, 1.96 mmol) as well as BF_3_·OEt_2_ (ρ = 1.12 g/mL, 166 µL, 1.31 mmol) at 0 °C. After complete conversion (2 h, LCMS) at this temperature, the reaction mixture was quenched with approx. 10 mL sat. aq. NaHCO_3_ solution. The aqueous phase was extracted three times with DCM and the combined organic phases were dried over Na_2_SO_4_ and concentrated under reduced pressure. The crude product was first purified by flash chromatography (silica gel, DCM/MeOH 95:5 → 9:1). Further purification was carried out by preparative HPLC (Luna, H_2_O/MeCN 9:1 → MeCN) to afford the deprotected cyclic heptapeptide **24** (41 mg, 42 μmol, 64%, mp. 168 °C) as a yellow solid. [α]_D_^20^ = −96.7 (c = 0.5, MeOH); Major rotamer: ^1^H-NMR ((CD_3_)_2_SO, 500 MHz) δ -0.22 (1 H, ddd, J = 12.9, 8.6, 4.3 Hz, H-30), 0.29 (3 H, d, J = 6.7 Hz, H-32), 0.46 (3 H, d, J = 6.6 Hz, H-32‘), 0.77–0.84 (3 H, m, H-48), 0.81–0.90 (6 H, m, H-45, H-48‘), 0.95–1.05 (1 H, m, H-31), 1.14 (3 H, d, J = 6.7 Hz, H-40), 1.24–1.33 (1 H, m, H-43), 1.45–1.59 (4 H, m, H-30‘, H-42, H-46, H-47), 1.47 (3 H, d, J = 6.3 Hz, H-18), 1.62 (1 H, dd, J = 9.3, 7.7 Hz, H-46‘), 1.72–1.82 (1 H, ddd, J = 14.3, 9.6, 4.4 Hz, H-42‘), 2.27–2.38 (1 H, m, H-15), 2.42–2.53 (1 H, m, H-15‘), 2.50 (3 H, s, H-29), 2.55–2.60 (1 H, m, H-33), 2.61 (3 H, s, H-41), 2.92 (1 H, dd, J = 13.2, 9.1 Hz, H-33‘), 3.04 (1 H, dd, J = 13.6, 5.2 Hz, H-19), 3.13–3.20 (1 H, m, H-44), 3.23–3.30 (2 H, m, H-19‘, H-44‘), 3.71 (3 H, s, H-28), 4.18–4.26 (1 H, m, H-2), 4.39 (1 H, dt, J = 5.4, 5.4 Hz, H-14), 4.46–4.53 (1 H, m, H-8), 4.54–4.61 (1 H, m, Hyleu-OH), 4.56 (1 H, t, J = 5.1 Hz, H-10), 4.70–4.77 (1 H, m, H-6), 4.77–4.86 (2 H, m, H-4, H-12), 5.03 (1 H, dt, J = 15.1, 7.3 Hz, H-16), 5.31 (1 H, dq, J = 8.7, 6.4 Hz, H-17), 6.95 (1 H, d, J = 8.5 Hz, H-38), 7.00 (1 H, dd, J = 7.4, 7.4 Hz, H-25), 7.07 (1 H, s, H-21), 7.13 (1 H, dd, J = 7.6, 7.6 Hz, H-24), 7.17 (1 H, dd, J = 8.7, 2.1 Hz, H-39), 7.38 (1 H, d, J = 8.2 Hz, H-23), 7.50 (1 H, d, J = 7.9 Hz, H-26), 7.51–7.55 (1 H, m, NH_e_), 7.54 (1 H, d, J = 2.1 Hz, H-35), 8.56-8.62 (2 H, m, NH_a_, NH_c_), 9.07 (1 H, d, J = 4.4 Hz, NH_b_), 9.23 (1 H, d, J = 6.7 Hz, NH_d_) 10.69 (1 H, s, Tyr-OH); Minor rotamer: ^1^H-NMR ((CD_3_)_2_SO, 500 MHz) δ 0.50 (3 H, d, J = 6.4 Hz, H-48), 0.56 (3 H, d, J = 6.4 Hz, H-48’), 1.18 (3 H, d, J = 6.9 Hz, H-40), 1.44 (3 H, d, J = 4.9 Hz, H-18), 2.59 (3 H, s, H-41), 3.72 (3 H, s, H-28); Major rotamer: ^13^C-NMR ((CD_3_)_2_SO, 125 MHz) δ 16.6 (q, C-40), 17.0 (q, C-45), 18.0 (q, C-18), 20.9 (q, C-32), 21.3 (q, C-48), 22.7 (q, C-48’), 22.9 (q, C-32’), 23.6 (d, C-31), 24.1 (d, C-47), 26.9 (t, C-19), 28.1 (q, C-41), 28.5 (q, C-29), 31.7 (d, C-43), 32.2 (q, C-28), 32.5 (t, C-42), 34.4 (t, C-15), 36.7 (t, C-33), 36.8 (t, C-30), 41.9 (t, C-46), 45.0 (d, C-6), 50.0 (d, C-12), 51.3 (d, C-14), 53.1 (d, C-2), 53.3 (d, C-8), 57.3 (d, C-4), 57.5 (d, C-10), 66.4 (t, C-44), 108.6 (s, C-20), 109.6 (d, C-23), 118.5 (d, C-26), 118.6 (d, C-25), 118.9 (d, C-38), 121.2 (d, C-24), 124.8 (d, C-16), 125.6 (d, C-35), 127.3 (s, C-34), 127.6 (s, C-27), 128.1 (d, C-21), 128.4 (d, C-17), 136.1 (d, C-39), 136.2 (s, C-36), 136.4 (s, C-22), 151.0 (s, C-37), 167.4 (s, C-9), 169.4 (s, C-3), 170.3 (s, C-7), 170.7 (s, C-1), 171.3 (s, C-13), 171.4 (s, C-11), 172.3 (s, C-5); Minor rotamer: ^13^C-NMR ((CD_3_)_2_SO, 125 MHz) δ 16.3 (q, C-40), 16.8 (q, C-45), 17.7 (q, C-18), 21.0 (q; C-48), 22.7 (q, C-48’); HRMS (ESI) *m*/*z* calculated for C_50_H_72_N_9_O_11_ [M + H]^+^ 974.5346, found 974.5344.

#### 4.3.3. Synthesis of (2*S*,5*S*,8*S*,11*S*,14*S*,17*S*,20*S*,22*S*,23*R*)-5-[(*E*)-But-2-en-1-yl]-23-hydroxy-14-(4-hydroxy-3-nitrobenzyl)-2,11-diisobutyl-10,17,19,22-tetramethyl-8-[(1-methyl-1*H*-indol-3-yl)methyl]-1,4,7,10,13,16,19-heptaazabicyclo[18.3.1]tetracosane-3,6,9,12,15,18,24-heptaone (**26**)

To a solution of **24** (5.0 mg, 5.1 μmol) in 5.1 mL anhydrous DCM was added pyridine (ρ = 0.982 g/mL, 8.3 µL, 0.1 mmol) as well as Dess-Martin periodinane (10.9 mg, 26.0 μmol) at 0 °C. The ice bath was removed and the reaction mixture was stirred for 1 h at room temperature. After complete conversion (LCMS), the reaction solution was quenched with approx. 1.6 mL sat. aq. Na_2_SO_3_ solution. The aqueous phase was extracted three times with EtOAc and the combined organic phases were washed with sat. aq. CuSO_4_ solution and brine, dried over Na_2_SO_4_ and concentrated. After dissolving the residue in 2 mL MeOH, the reaction mixture was cooled again to 0 °C and K_2_CO_3_ (3.6 mg, 26 μmol) was added. Overnight the reaction solution was slowly warmed to room temperature (18 h). The reaction mixture was quenched with 1.5 mL sat. aq. NH_4_Cl solution and the aqueous phase was extracted three times with EtOAc. The combined organic phases were washed with 1 M aq. NH_4_Cl solution and brine and dried over Na_2_SO_4_. The solvent was removed in vacuo and the crude product was purified by reverse phase flash chromatography (C18 silica gel, H_2_O/MeCN 9:1 → MeCN). Further purification was carried out by preparative HPLC (Luna, H_2_O/MeCN 9:1 → MeCN). The desired ilamycin derivative **26** (2.0 mg, 2.1 μmol, 40%) was isolated as a yellow amorphous solid. [α]_D_^20^ = −98.7 (c = 0.25, CHCl_3_); Major rotamer: ^1^H-NMR (CD_3_OD, 500 MHz) δ -0.46 (1 H, ddd, J = 13.1, 8.7, 4.0 Hz, H-30), 0.24 (3 H, d, J = 6.6 Hz, H-32), 0.48 (3 H, d, J = 6.6 Hz, H-32’), 0.92 (3 H, d, J = 6.4 Hz, H-48), 0.90–1.00 (1 H, m, H-31), 1.02 (3 H, d, J = 6.7 Hz, H-48’), 1.09 (3 H, d, J = 6.7 Hz, H-45), 1.27 (3 H, d, J = 6.6 Hz, H-40), 1.38–1.48 (2 H, m, H-30’, H-47), 1.66 (3 H, dd, J = 6.1, 1.1 Hz, H-18), 1.83–1.89 (1 H, m, H-42), 1.88–1.96 (2 H, m, H-46), 1.94–2.00 (1 H, m, H-43), 2.23–2.29 (1 H, m, H-42’), 2.29 (3 H, s, H-29), 2.57–2.65 (1 H, m, H-15), 2.76–2.85 (1 H, m, H-15’), 2.91 (1 H, dd, J = 14.4, 10.3 Hz, H-33), 3.09 (1 H, dd, J = 14.2, 5.8 Hz, H-33’), 3.13–3.27 (2 H, m, H-19), 3.25 (3 H, s, H-41), 3.74 (3 H, s, H-28), 3.74–3.82 (1 H, m, H-4), 4.21 (1 H, dd, J = 10.3, 3.9 Hz, H-10), 4.57 (1 H, t, J = 7.4 Hz, H-14), 4.62 (1 H, dd, J = 10.2, 5.7 Hz, H-8), 4.77 (1 H, d, J = 1.5 Hz, H-44), 4.77–4.82 (1 H, m, H-6), 4.86–4.94 (1 H, m, H-12), 5.22 (1 H, dd, J = 11.3, 5.1 Hz, H-2), 5.52–5.60 (1 H, m, H-16), 5.60–5.68 (1 H, m, H-17), 7.00–7.07 (3 H, m, H-21, H-25, H-38), 7.13–7.19 (1 H, m, H-24), 7.21 (1 H, d, J = 8.2 Hz, H-23), 7.37 (1 H, dd, J = 9.0, 1.5 Hz, H-39), 7.44 (1 H, d, J = 7.9 Hz, H-26), 7.83 (1 H, d, J = 2.0 Hz, H-35); Minor rotamer: ^1^H-NMR (CD_3_OD, 500 MHz) δ 0.27 (3 H, d, J = 6.6 Hz, H-32), 0.45 (3 H, d, J = 6.1 Hz, H-32’), 1.61 (3 H, d, J = 6.4 Hz, H-18); ^13^C-NMR (CD_3_OD, 125 MHz) δ 17.7 (q, C-45), 18.0 (q, C-40), 18.5 (q, C-18), 21.4 (q, C-48), 21.8 (q, C-32), 23.4 (q, C-32’), 24.0 (q, C-48’), 25.9 (d, C-31), 26.0 (d, C-47), 27.0 (t, C-42), 29.5 (t, C-19), 29.5 (q, C-29), 33.0 (q, C-28), 34.4 (d, C-43), 35.2 (t, C-15), 36.0 (t, C-46), 37.4 (t, C-30), 38.0 (t, C-33), 38.6 (q, C-41), 47.8 (d, C-6), 52.3 (d, C-12), 54.4 (d, C-14), 55.6 (d, C-2), 57.4 (d, C-8), 59.7 (d, C-10), 63.3 (d, C-4), 79.6 (d, C-44), 109.7 (s, C-20), 110.6 (d, C-23), 119.9 (d, C-26), 120.3 (d, C-25), 121.6 (d, C-38), 123.0 (d, C-24), 126.6 (d, C-35), 127.8 (d, C-16), 129.0 (s, C-34), 129.3 (d, C-21), 129.4 (s, C-27), 129.5 (d, C-17), 135.9 (s, C-36), 138.5 (s, C-22), 139.0 (d, C-39), 156.3 (s, C-37), 170.2 (s, C-9), 172.0 (s, C-7), 172.2 (s, C-3), 172.7 (s, C-5), 173.3 (s, C-13), 173.3 (s, C-1), 174.4 (s, C-11); HRMS (ESI) *m*/*z* calculated for C_50_H_70_N_9_O_11_ [M + H]^+^ 972.5189, found 972.5189.

#### 4.3.4. Synthesis of (*S*)-3-[(2*S*,5*S*,8*S*,11*S*,14*S*,17*S*,20*S*)-8-{(*E*)-But-2-en-1-yl}-17-[4-hydroxy-3-nitro-benzyl]-5,14-diisobutyl-1,13,20-trimethyl-11-{(1-methyl-1*H*-indol-3-yl)-methyl}-3,6,9,12,15,18,21-heptaoxo-1,4,7,10,13,16,19-heptaazacyclo-henicosan-2-yl]-2-methylpropanoic Acid (**27**)

To a solution of **24** (5.0 mg, 5.1 μmol) in 5.1 mL dry DCM was added pyridine (ρ = 0.982 g/mL, 8.3 µL, 0.1 mmol) as well as Dess-Martin periodinane (10.9 mg, 26.0 μmol) at 0 °C. The ice bath was removed and the reaction mixture was stirred for 1 h at room temperature. After complete conversion (LCMS), the reaction solution was quenched with approx. 1.6 mL sat. aq. Na_2_SO_3_ solution. The aqueous phase was extracted three times with EtOAc and the combined organic phases were washed with sat. aq. CuSO_4_ solution and brine, dried over Na_2_SO_4_ and concentrated. After the residue was dissolved in 0.3 mL of *t*-BuOH and water, NaH_2_PO_4_ dihydrate (16 mg, 0.1 mmol) and 2-methyl-2-butene (ρ = 0.66 g/mL, 27 µL, 0.26 mmol) were added to the reaction mixture at room temperature followed by the addition of sodium chlorite (5.8 mg, 51 μmol) at 0 °C. The ice bath was removed and stirring was continued for two hours at room temperature. The reaction mixture was diluted with approx. 2.6 mL EtOAc and 0.6 mL water and the phases were separated. The aqueous phase was extracted three times with EtOAc and the combined organic phases were washed with water and brine, dried over Na_2_SO_4_ and concentrated under reduced pressure. After purification by reverse phase flash chromatography (C18 silica gel, H_2_O/MeCN 9:1 → MeCN), the crude product was further purified via preparative HPLC (Luna, H_2_O/MeCN 9:1 → MeCN) to give the desired ilamycin derivative **27** (2.0 mg, 2.0 μmol, 39%) as a yellowish amorphous solid. [α]_D_^20^ = −79.0 (c = 0.25, CHCl_3_); Major rotamer: ^1^H-NMR (CD_3_OD, 500 MHz) δ -0.61 (1 H, dd, J = 12.8, 9.7, 3.1 Hz, H-30), 0.24 (3 H, d, J = 6.6 Hz, H-32), 0.44 (3 H, d, J = 6.7 Hz, H-32’), 0.95 (3 H, d, J = 5.8 Hz, H-48), 0.98 (3 H, d, J = 5.7 Hz, H-48’), 1.00–1.08 (1 H, m, H-31), 1.23 (3 H, d, J = 7.2 Hz, H-45), 1.27 (3 H, d, J = 6.9 Hz, H-40), 1.50–1.59 (1 H, m, H-30), 1.56 (3 H, d, J = 6.3 Hz, H-18), 1.68–1.78 (3 H, m, H-46, H-47), 1.82 (1 H, ddd, J = 13.0, 9.4, 4.5 Hz, H-42), 2.14–2.24 (1 H, m, H-42’), 2.42–2.49 (1 H, m, H-43), 2.46–2.54 (1 H, m, H-15), 2.61 (3 H, s, H-41), 2.69 (1 H, dd, J = 13.1, 4.7 Hz, H-33), 2.73 (3 H, s, H-29), 2.74–2.84 (1 H, m, H-15’), 3.03 (1 H, dd, J = 13.0, 9.9 Hz, H-33’), 3.15 (1 H, dd, J = 13.5, 3.7 Hz, H-19), 3.43 (1 H, dd, J = 13.1, 10.8 Hz, H-19’), 3.76 (3 H, s, H-28), 4.54–4.60 (3 H, m, H-8, H-10, H-14), 4.60–4.67 (1 H, m, H-2), 4.75–4.83 (1 H, m, H-6), 4.88–4.94 (1 H, m, H-12), 4.97 (1 H, dd, J = 8.5, 4.6 Hz, H-4), 5.17 (1 H, dt, J = 14.8, 7.0 Hz, H-16), 5.49 (1 H, dq, J = 15.0, 6.5 Hz, H-17), 6.97 (1 H, s, H-21), 7.02–7.09 (1 H, m, H-25), 7.06 (1 H, d, J = 8.5 Hz, H-38), 7.18 (1 H, dd, J = 7.3, 7.3 Hz, H-24), 7.36 (1 H, d, J = 8.2 Hz, H-23), 7.41 (1 H, dd, J = 8.6, 2.1 Hz, H-39), 7.52 (1 H, d, J = 7.9 Hz, H-26), 7.78 (1 H, d, J = 2.1 Hz, H-35); Minor rotamer: ^1^H-NMR (CD_3_OD, 500 MHz) δ 0.56 (3 H, d, J = 6.6 Hz, H-32), 0.64 (3 H, d, J = 6.6 Hz, H-32’), 0.90 (3 H, d, J = 5.7 Hz, H-48’), 0.92 (3 H, d, J = 5.8 Hz, H-48), 1.36 (3 H, d, J = 6.9 Hz, H-40), 1.52 (3 H, d, J = 6.0 Hz, H-18), 2.77 (3 H, s, H-29), 3.77 (3 H, s, H-28), 7.62 (1 H, d, J = 7.9 Hz, H-26), 7.90 (1 H, d, J = 2.0 Hz, H-35); Major rotamer: ^13^C-NMR (CD_3_OD, 125 MHz) δ 17.1 (q, C-40), 18.7 (q, C-18), 19.3 (q, C-45), 21.4 (q, C-32), 22.2 (q, C-48), 23.3 (q, C-48’), 23.6 (q, C-32’), 25.6 (d, C-31), 26.1 (d, C-47), 28.6 (t, C-19), 30.0 (q, C-41), 30.0 (q, C-29), 33.0 (q, C-28), 35.2 (t, C-42), 36.2 (t, C-15), 38.2 (t, C-30), 38.4 (t, C-33), 39.0 (d, C-43), 45.1 (t, C-46), 47.0 (d, C-6), 52.5 (d, C-12), 53.5 (d, C-14), 55.0 (d, C-2), 55.7 (d, C-8), 60.1 (d, C-10), 60.2 (d, C-4), 110.0 (s, C-20), 110.8 (d, C-23), 119.8 (d, C-26), 120.4 (d, C-38), 121.3 (d, C-25), 123.1 (d, C-24), 125.5 (d, C-16), 126.9 (d, C-35), 129.2 (s, C-27), 129.3 (d, C-21), 129.8 (s, C-34), 131.4 (d, C-17), 135.7 (s, C-36), 138.7 (s, C-22), 139.3 (d, C-39), 154.8 (s, C-37), 169.9 (s, C-9), 171.1 (s, C-3), 172.5 (s, C-7), 173.2 (s, C-13), 174.0 (s, C-1), 174.2 (s, C-11), 175.1 (s, C-5), 181.0 (s, C-44); Minor rotamer: ^13^C-NMR (CD_3_OD, 125 MHz) δ 16.8 (q, C-40), 18.4 (q, C-18), 21.6 (q, C-32), 23.4 (q, C-32’); HRMS (ESI) *m*/*z* calculated for C_50_H_70_N_9_O_12_ [M + H]^+^ 988.5138, found 988.5139.

## 5. Conclusions

The ilamycins/rufomycins are highly interesting marine cycloheptapeptides characterized by their incorporation of unusual amino acids. The natural products are produced by *Streptomyces* sp. and show potent activity against a range of mycobacteria, including multidrug-resistant strains of *Mycobacterium tuberculosis*. Simplified derivatives 26 and 27 of the ilamycins are synthesized. Key steps in the synthesis of the unusual amino acid building brocks are an asymmetric hydrogenation for the synthesis of the required δ-hydroxyleucine derivative and a chelate enolate Claisen rearrangement to generate the γ,δ-unsaturated amino acid. The ilamycin derivative **26** with a cyclic hemiaminal structure is highly active against *M. tuberculosis* (MIC: 50 nM) as well as *M. smegmatis* and *M. marinum* (MIC: 0.26 µM) and shown no significant cytotoxicity. In contrast, derivative 27, with a glutamic acid at this position was significantly less active with MIC`s in the mid µM-range. Detailed investigations of the mode of action of 26 indicate that **26** deregulates ClpC1 activity and strongly enhances ClpC1-WT ATPase activity. Degradation of FITC-casein, which binds to the NTD like ClpC1 targeting cyclic peptides, remained largely unaffected by **26**. Notably, a strong inhibition of FITC-casein proteolysis by **26** is observed for ClpC1-F444S, a ClpC1 MD mutant, which is constitutively hexameric and thus has a high basal ATPase activity. Probably, **26** has dual effects, negative and positive ones, on ClpC1-WT function. The positive effect relates to ClpC1-WT ATPase activation and the negative one to substrate binding to the NTD. 

## Figures and Tables

**Table 1:** Biological activities of ilamycin derivatives **26** and **27**.

	MIC [μM]			IC_50_ [μM]
Compound	*M. smegmatis* [mc^2^155]	*M. marinum* [Strain M]	*M. tuberculosis* [H37Ra]	*HepG2*
**26**	0.26	0.26	0.05	24.0 ± 5.8
**27**	32.4	64.8	16.2	>37.4

## Data Availability

The authors confirm that the data supporting the findings of this study are available within the article or the Supplementary Materials.

## Supplementary Materials

The following supporting information can be downloaded at: https://www.mdpi.com/article/10.3390/md20100632/s1, Figure S1: Biochemical analysis of **26** impact on ClpC1 activity; Copies of NMR spectra of all new compounds.
