# Supplementary material for: Total Synthesis and Biological Evaluation of Modified Ilamycin Derivatives
Source: Mar Drugs. 2022 Oct 3;20(10):632. doi: 10.3390/md20100632 (PMC9605216; doi:10.3390/md20100632)
Supplement: Supplementary file 1 [file marinedrugs-20-00632-s001.zip › marinedrugs-1930177-supplementary.pdf]

Supporting Materials

Total Synthesis and Biological Evaluation of Modified Ilamycin Derivatives

Jennifer Greve,[1] Axel Mogk[2,3] and Uli Kazmaier [1*]

1 Organic Chemistry, Saarland University, Campus Building C4.2, D-66123 Saarbruecken, Germany;
2 Center for Molecular Biology of Heidelberg University (ZMBH), DKFZ-ZMBH Alliance, Im Neuenheimer Feld 282, 69120 Heidelberg, Germany
3 German Cancer Research Center (DKFZ), Im Neuenheimer Feld 280, 69120 Heidelberg, Germany
* Correspondence: u.kazmaier@mx.uni-saarland.de; Tel.: +49-681-302-3409

Copies of the NMR spectra and GC chromatogram

$^1H/^{13}C$-NMR Compound **2** S1

$^1H/^{13}C$-NMR Compound **3** S2

$^1H/^{13}C$-NMR Compound **4** S3

$^1H/^{13}C$-NMR Compound **5** S4

$^1H/^{13}C$-NMR Compound **7** S5

$^1H/^{13}C$-NMR Compound **9** S6

$^1H/^{13}C$-NMR Compound **10** S7

GC chromatogramm Compound **10** S8

$^1H/^{13}C$-NMR Compound **11** S9

$^1H/^{13}C$-NMR Compound **12** S10

$^1H/^{13}C$-NMR Compound **14** S11

$^1H/^{13}C$-NMR Compound **15** S12

$^1H/^{13}C$-NMR Compound **16** S13

$^1H/^{13}C$-NMR Compound **17** S14

$^1H/^{13}C$-NMR Compound **18** S15

$^1H/^{13}C$-NMR Compound **19** S16

$^1H/^{13}C$-NMR Compound **20** S17

$^1H/^{13}C$-NMR Compound **21** S18

$^1H/^{13}C$-NMR Compound **22** S19

$^1H/^{13}C$-NMR Compound **23** S20

$^1H/^{13}C$-NMR Compound **24** S21

$^1H/^{13}C$-NMR Compound **26** S22/23

$^1H/^{13}C$-NMR Compound **27** S24/25

Supplementary Figure S1 .... S26

(*S*)-2-{[(Allyloxy)carbonyl]amino}-3-(4-hydroxy-3-nitrophenyl)propanoic acid (2)

^{1}H-NMR (400 MHz, $CDCl_3$):

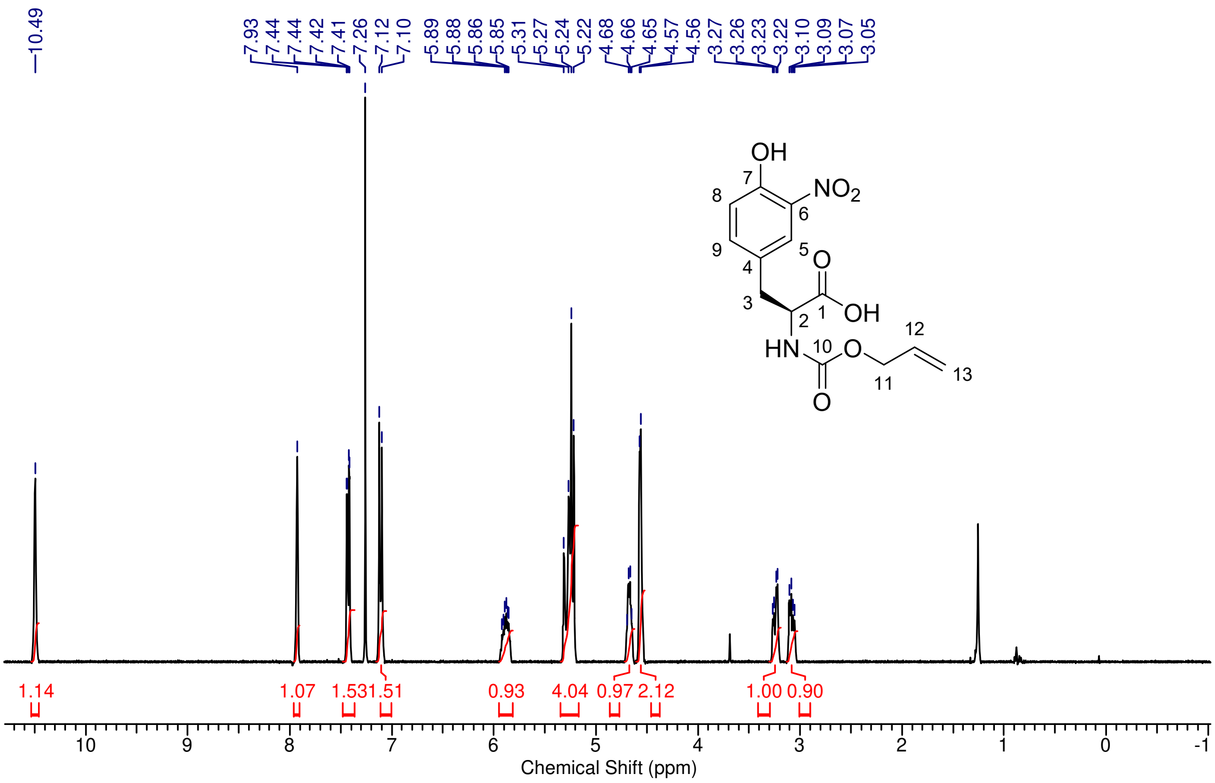

^{13}C-NMR (100 MHz, $CDCl_3$):

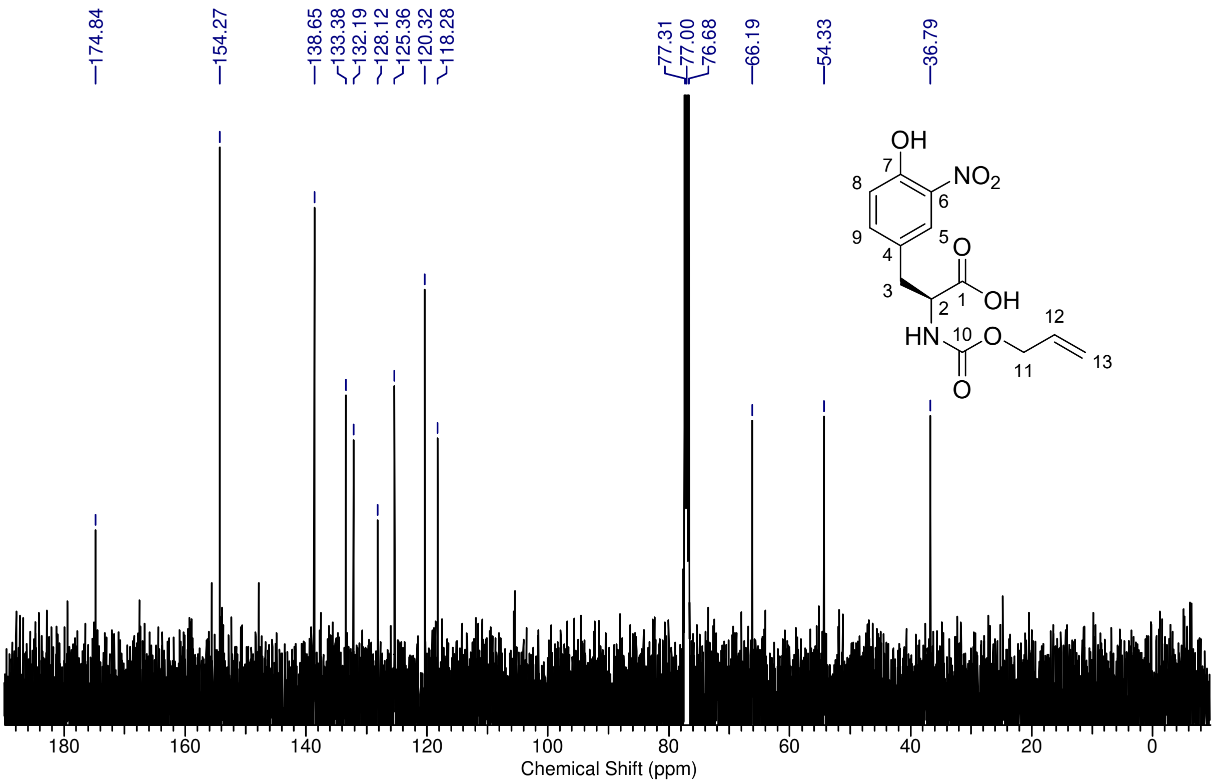

Methyl (*S*)-2-{[(allyloxy)carbonyl]amino}-3-(4-hydroxy-3-nitrophenyl)propanoate (3)

[1]**H-NMR** (400 MHz, CDCl3):

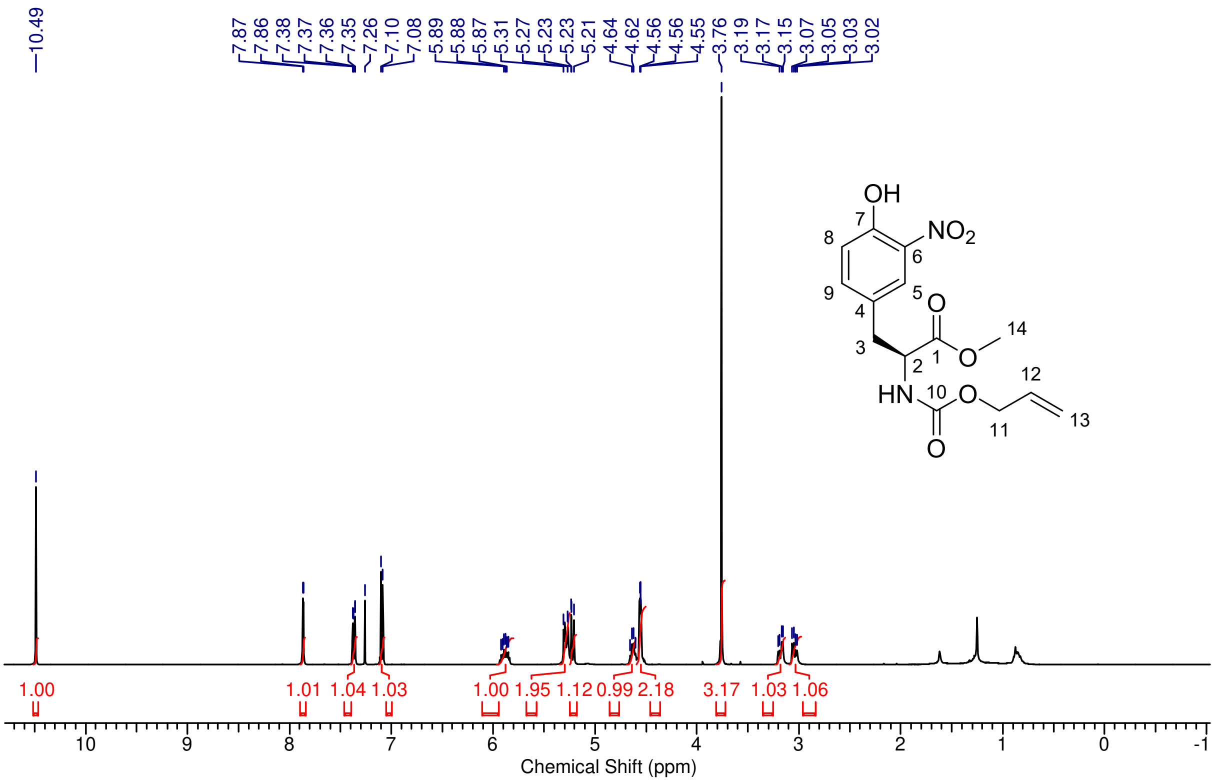

[13]**C-NMR** (100 MHz, CDCl3):

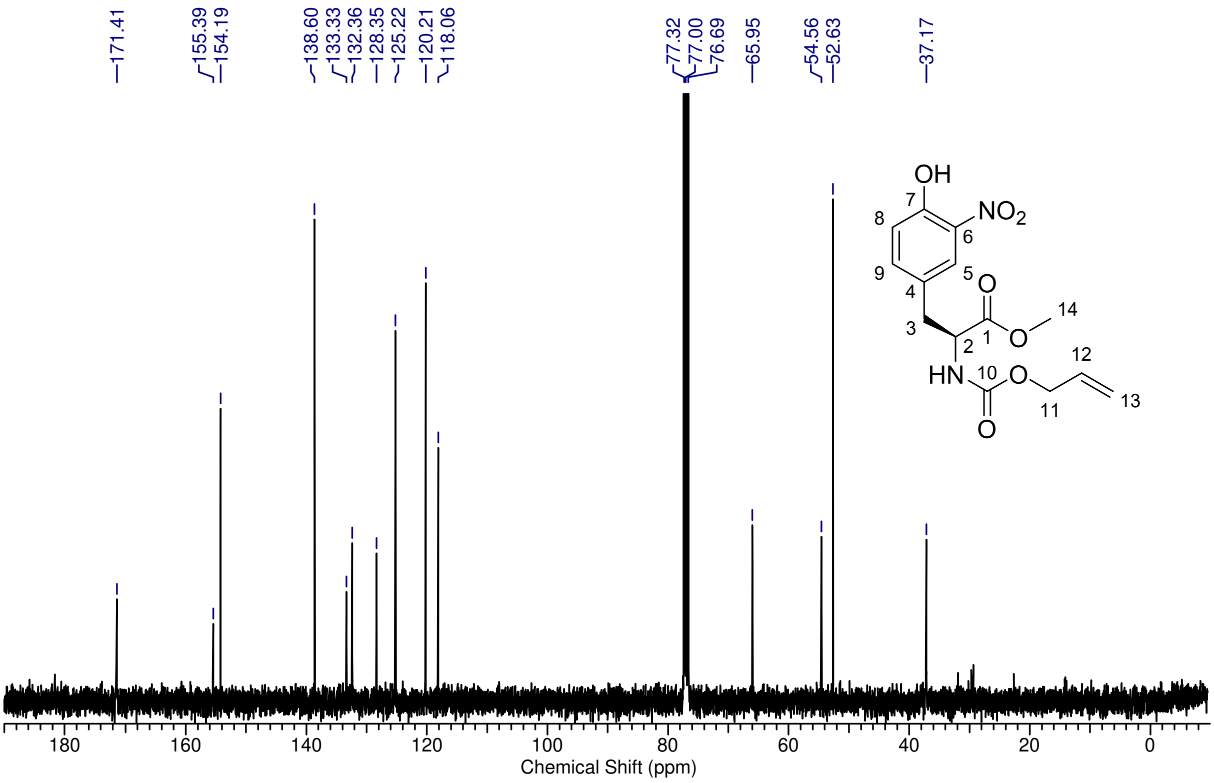

Methyl (*S*)-2-{[(allyloxy)carbonyl]amino}-3-{4-[(2-methoxyethoxy)methoxy]-3-nitrophenyl}-propanoate (4)

^{1}H-NMR (400 MHz, $CDCl_3$):

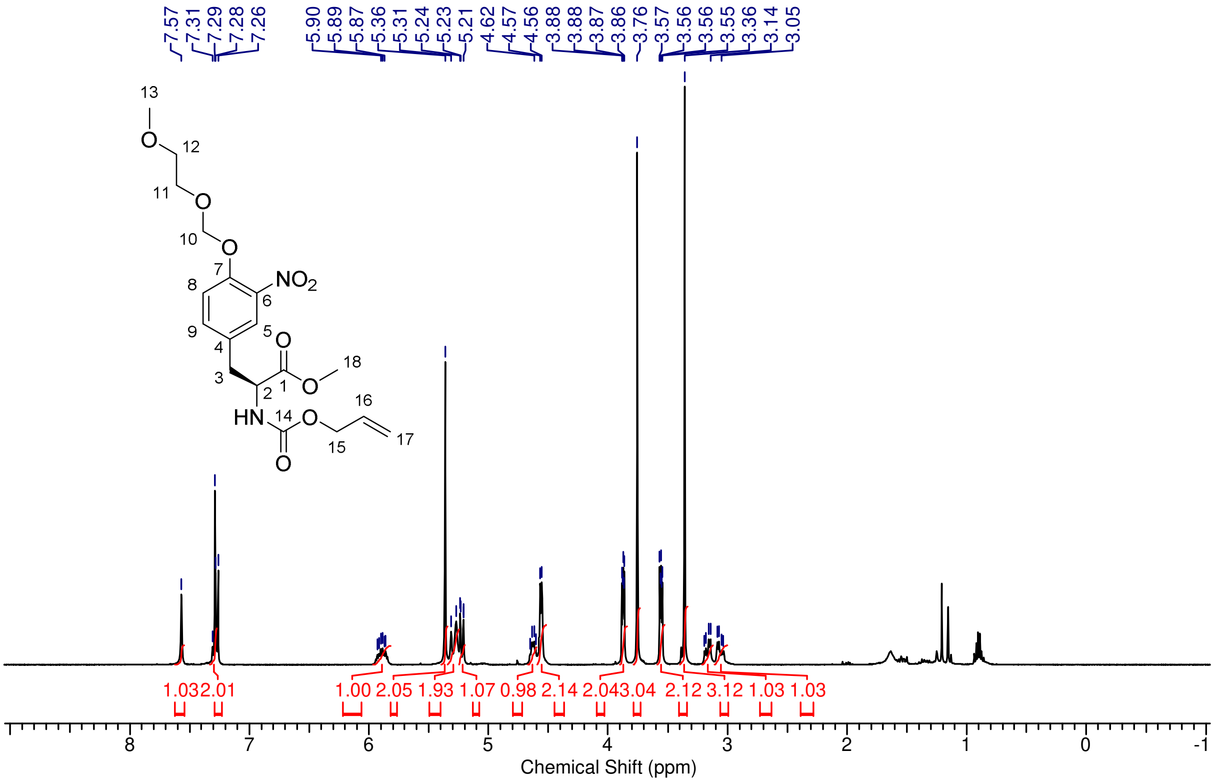

^{13}C-NMR (100 MHz, $CDCl_3$):

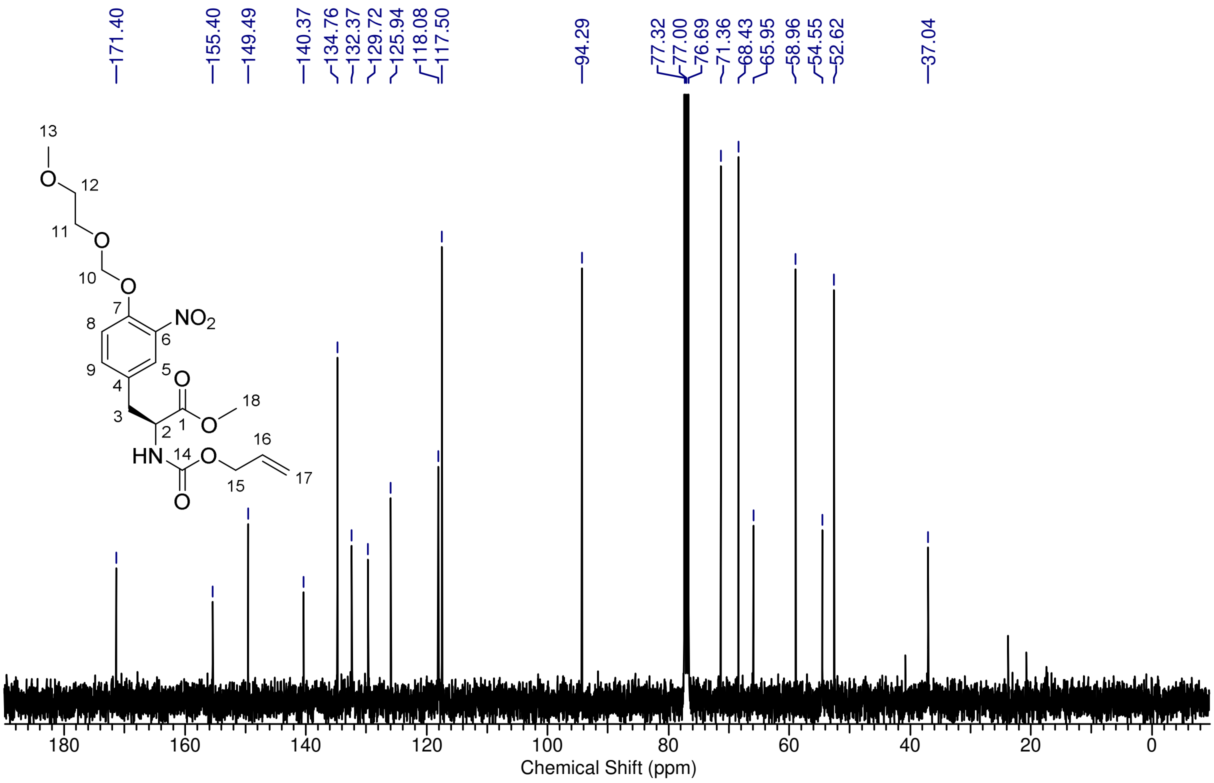

(*S*)-2-{[(Allyloxy)carbonyl]amino}-3-{4-[(2-methoxyethoxy)methoxy]-3-nitro-phenyl}propanoic acid (5)

¹H-NMR (400 MHz, CDCl₃):

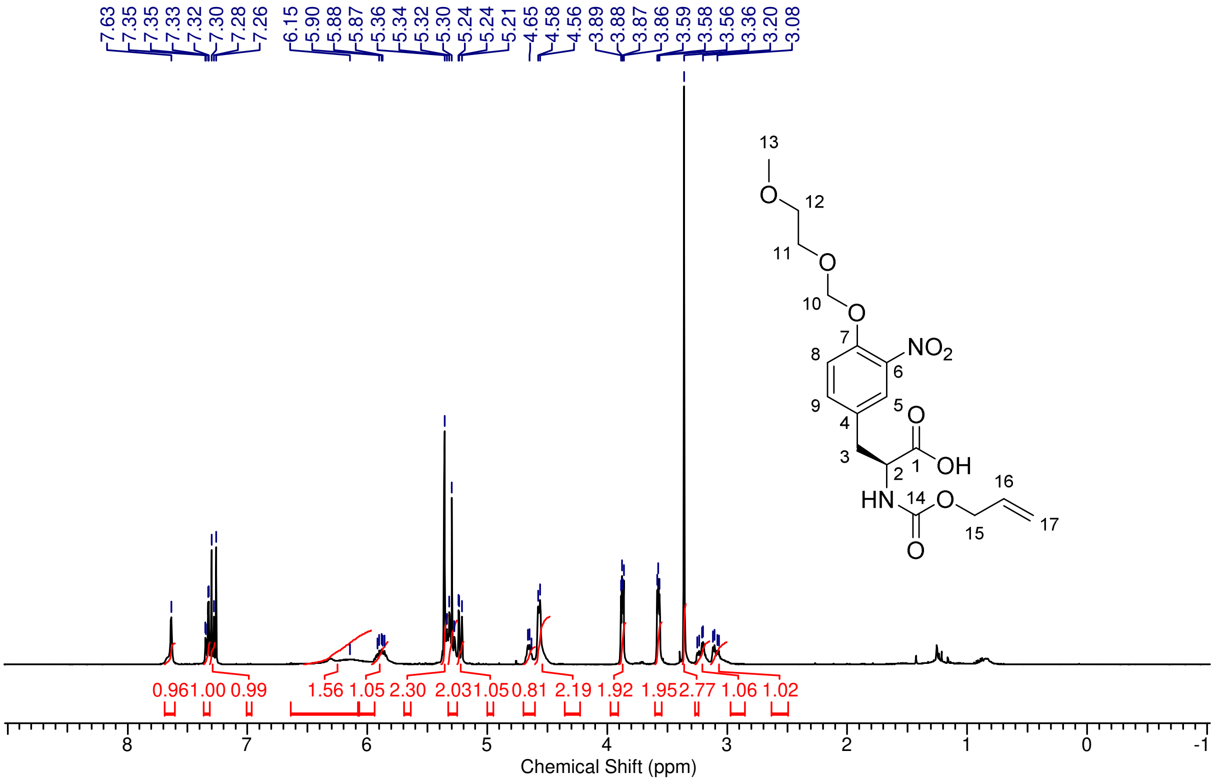

¹³C-NMR (100 MHz, CDCl₃):

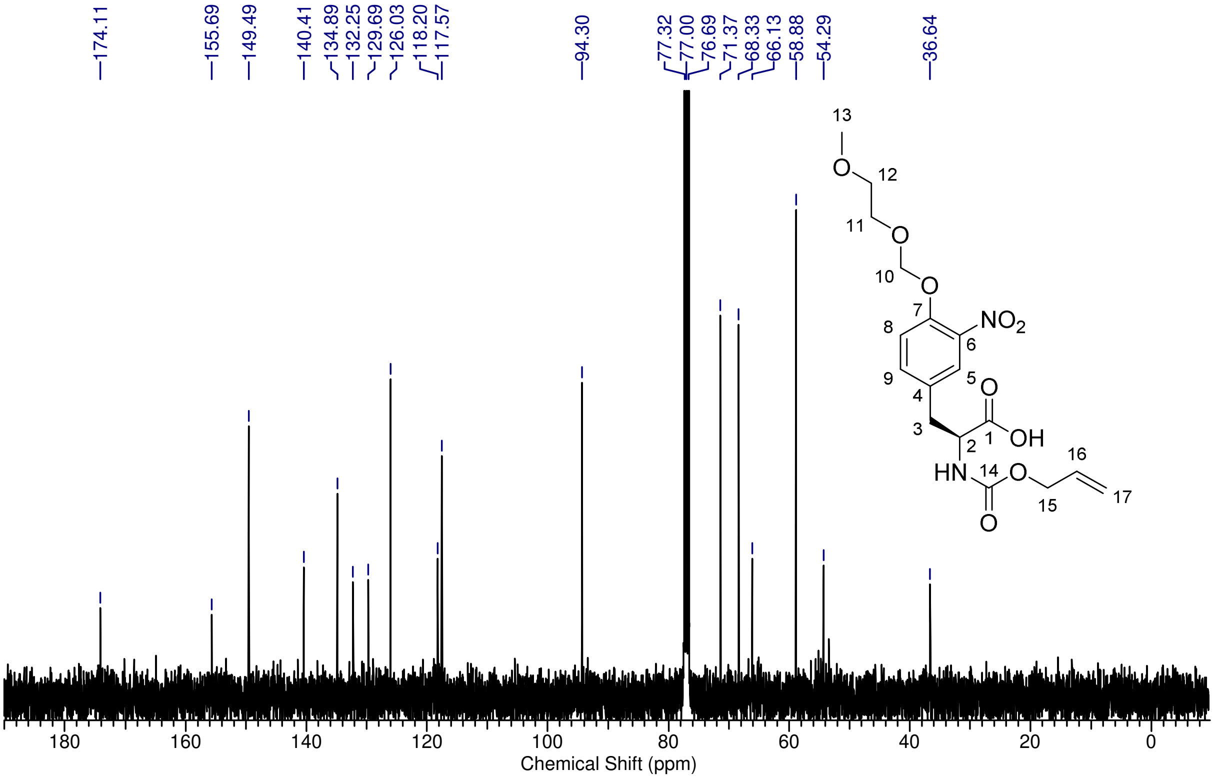

Methyl (*R*)-3-[(*tert*-butyldimethylsilyl)oxy]-2-methylpropanoate (7)

^{1}H-NMR (400 MHz, $CDCl_3$):

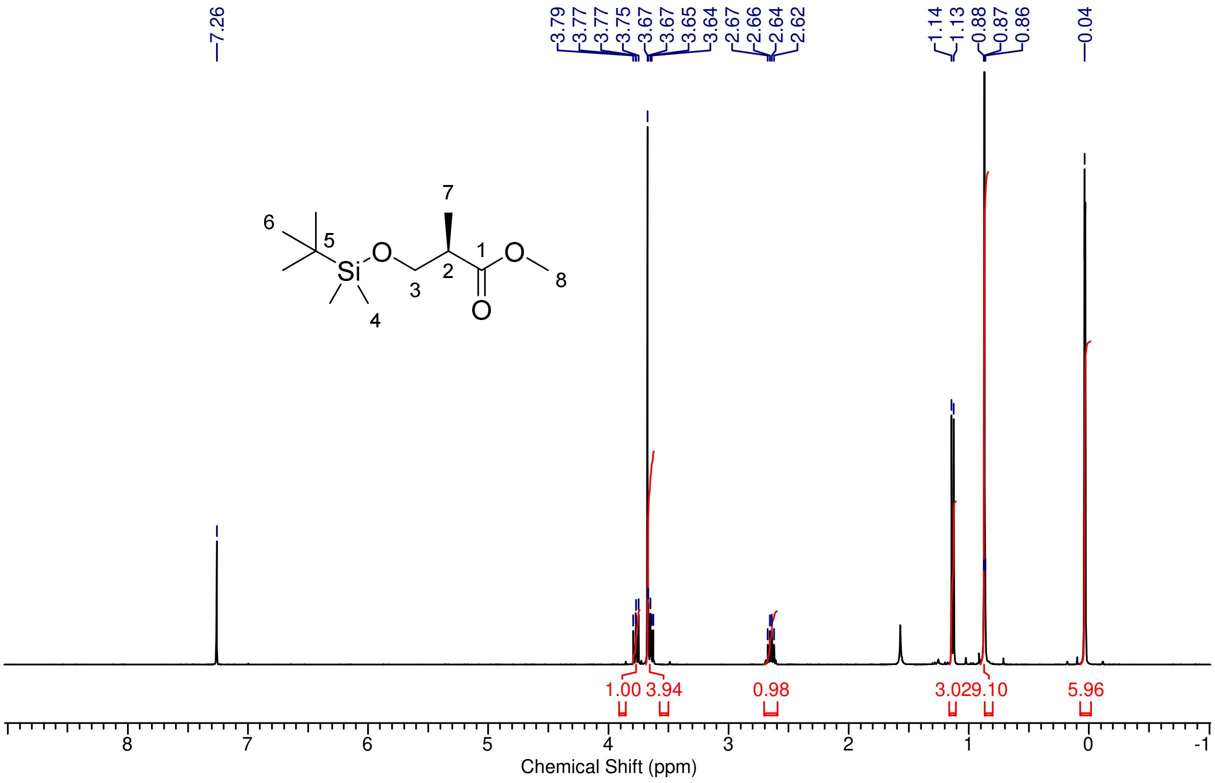

^{13}C-NMR (100 MHz, $CDCl_3$):

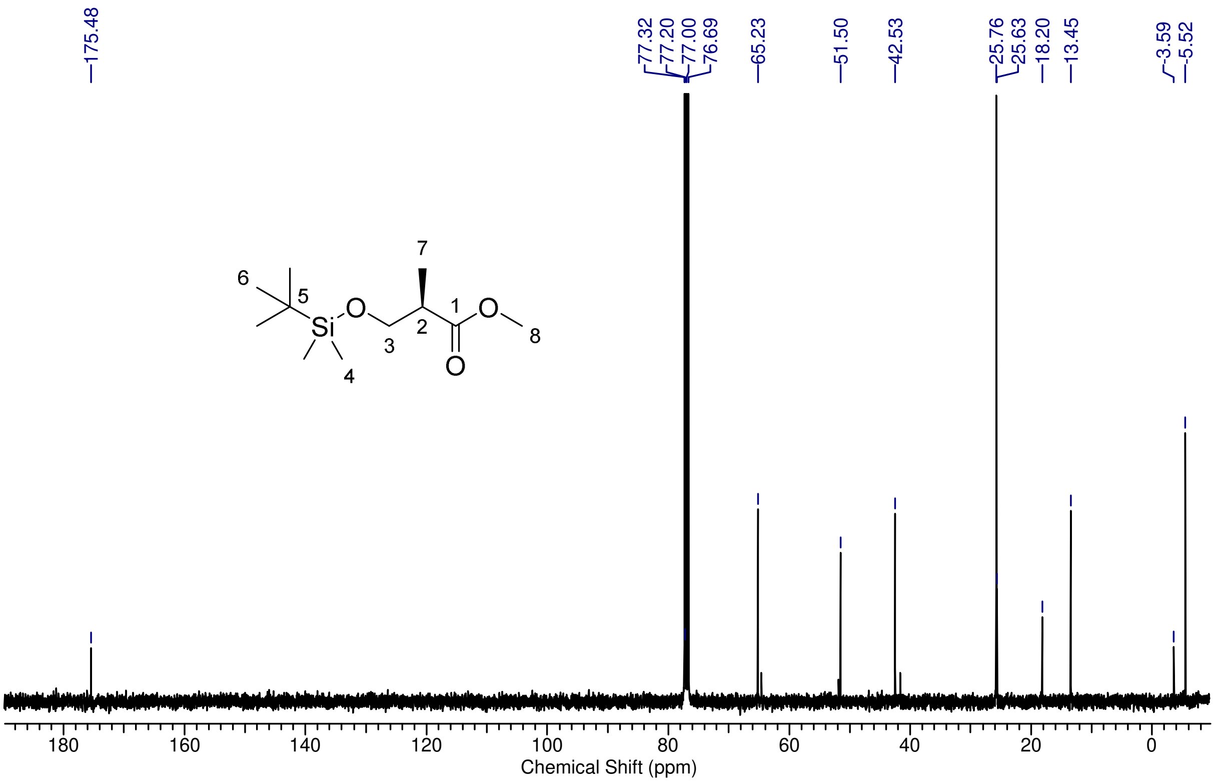

Methyl (*S*,*Z*)-2-{[(benzyloxy)carbonyl]amino}-5-[(*tert*-butyldimethylsilyl)oxy]-4-methylpent-2-enoate (9; *E*/*Z* ratio 1:3)

^{1}H-NMR (400 MHz, $CDCl_3$):

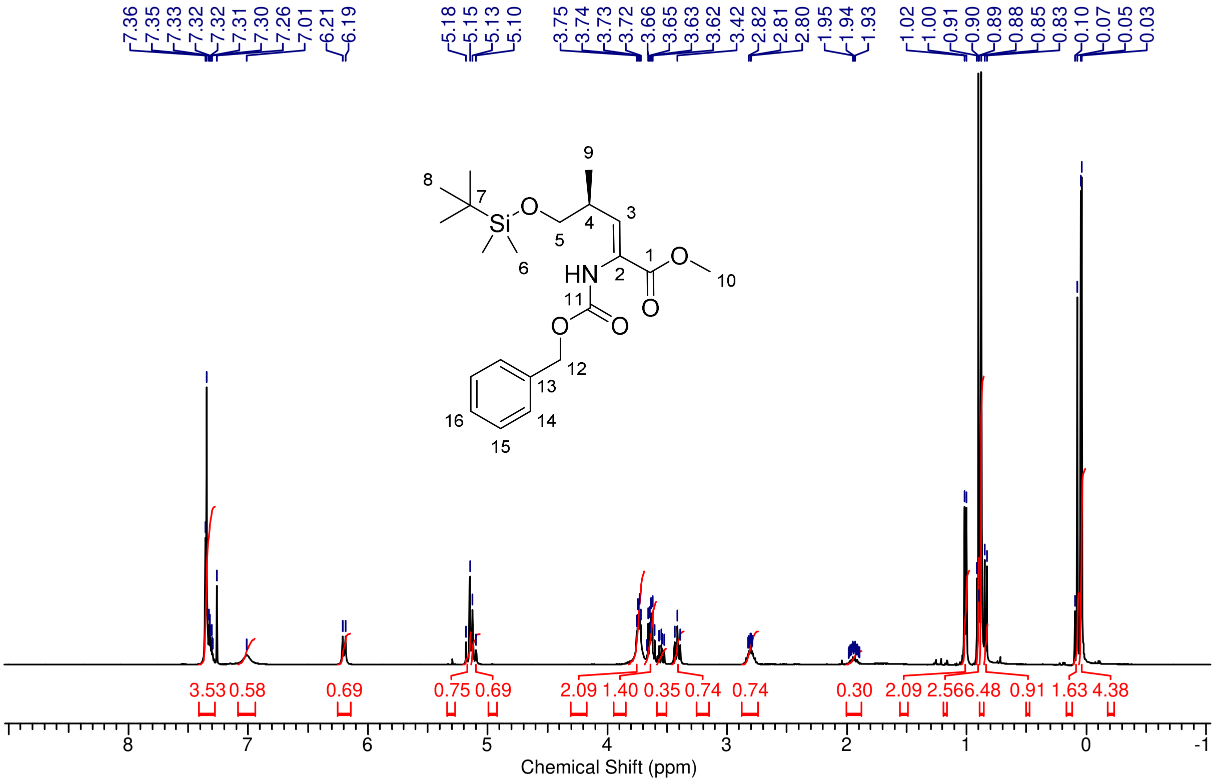

^{13}C-NMR (100 MHz, $CDCl_3$):

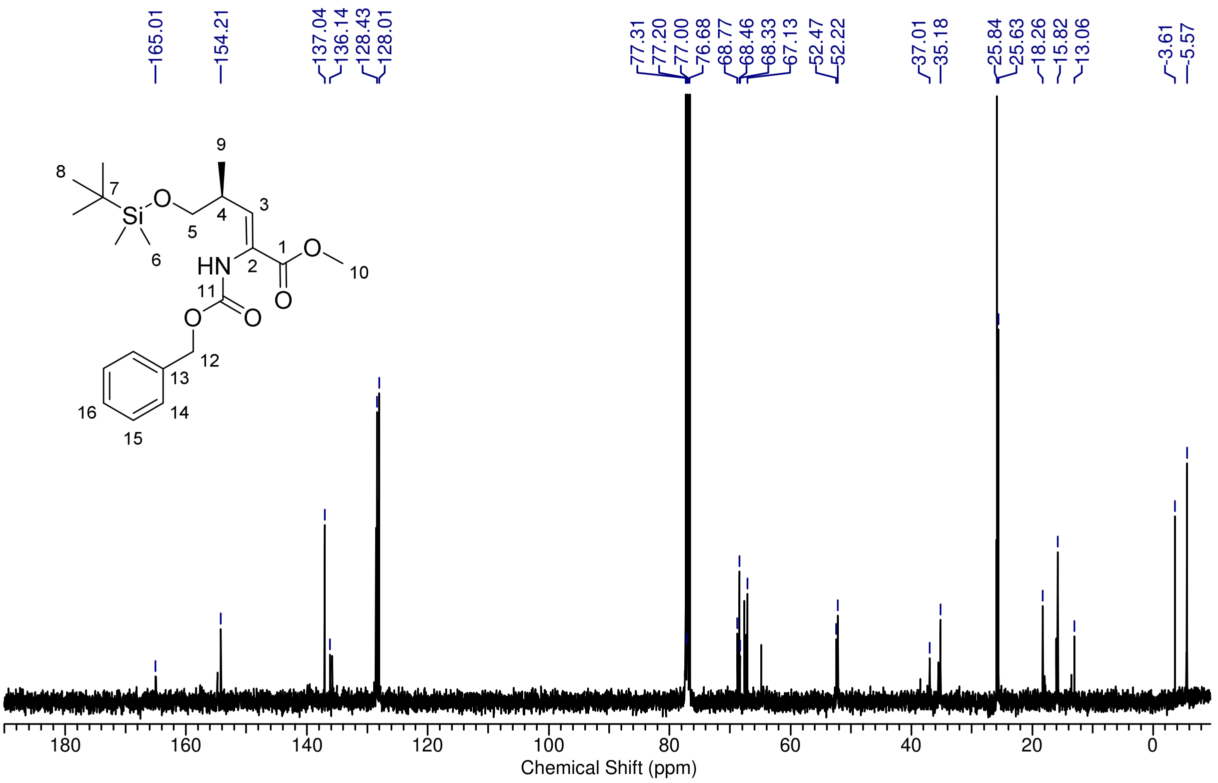

Methyl (2*S*,4*S*)-2-{[(benzyloxy)carbonyl]amino}-5-[(*tert*-butyldimethylsilyl)oxy]-4-methyl-pentanoate (10)

^{1}H-NMR (500 MHz, $CDCl_3$):

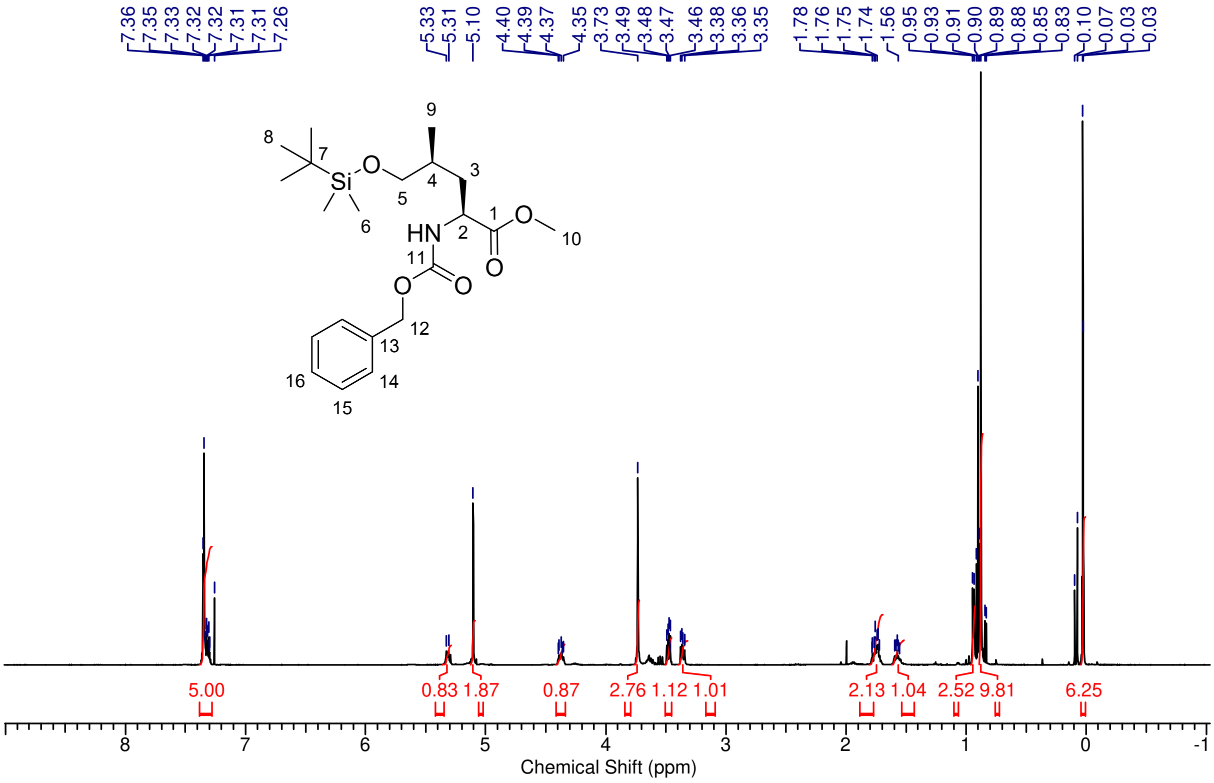

^{13}C-NMR (125 MHz, $CDCl_3$):

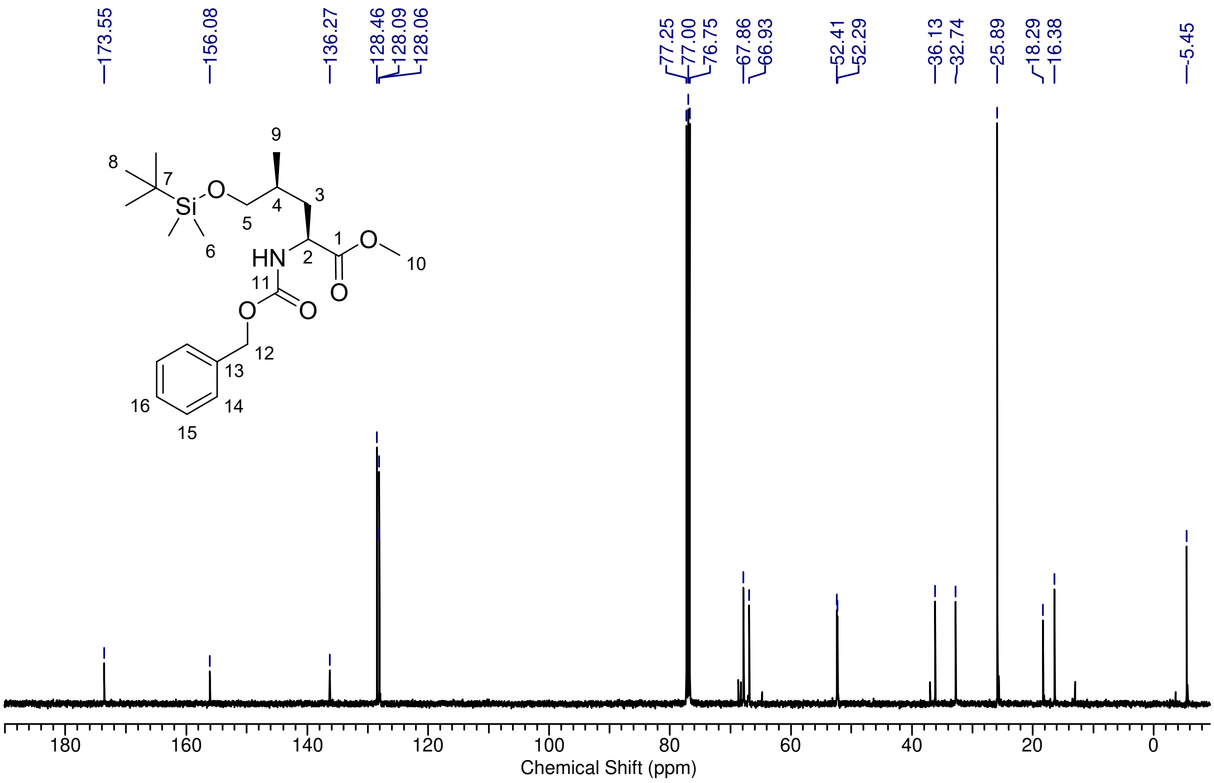

GC-FID:

Column: Agilent CP-Chirasil-Dex CB (25 m x 0.25 mm, 0.25 µm ID); **Carrier gas**: N_2

T_0 [1 min] = 110 °C, 2.0 °C/min to 180 °C, injector: 250 °C, detector: 275 °C

N-acetyl-(2*R*,4*S*): t_r = 32.42 min, *N*-acetyl-(2*S*,4*S*): t_r = 32.76 min.

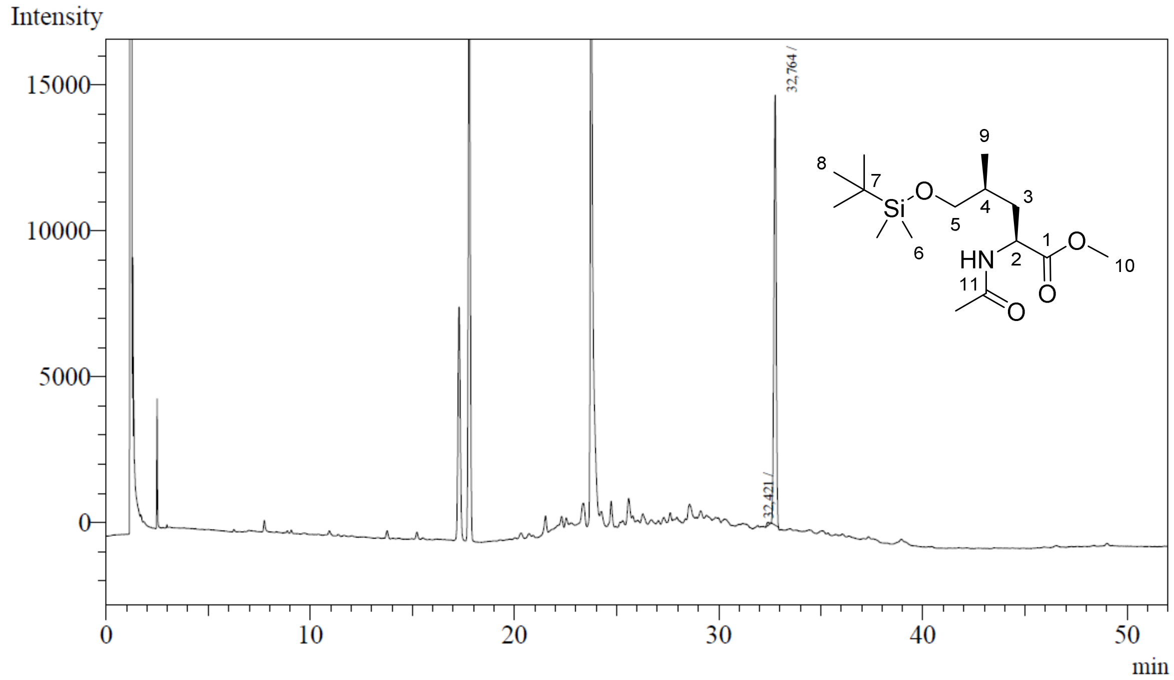

Peak#	Ret.Time	Area	Height	Conc.	Unit	Mark	ID#	Cmpd Name
1	32,421	977	105	0,841				
2	32,764	115222	14255	99,159				
Total		116199	14360					

(2*S*,4*S*)-2-{[(Benzyloxy)carbonyl]amino}-5-[(*tert*-butyldimethylsilyl)oxy]-4-methylpentanoic acid (11)

¹H-NMR (400 MHz, $CDCl_3$):

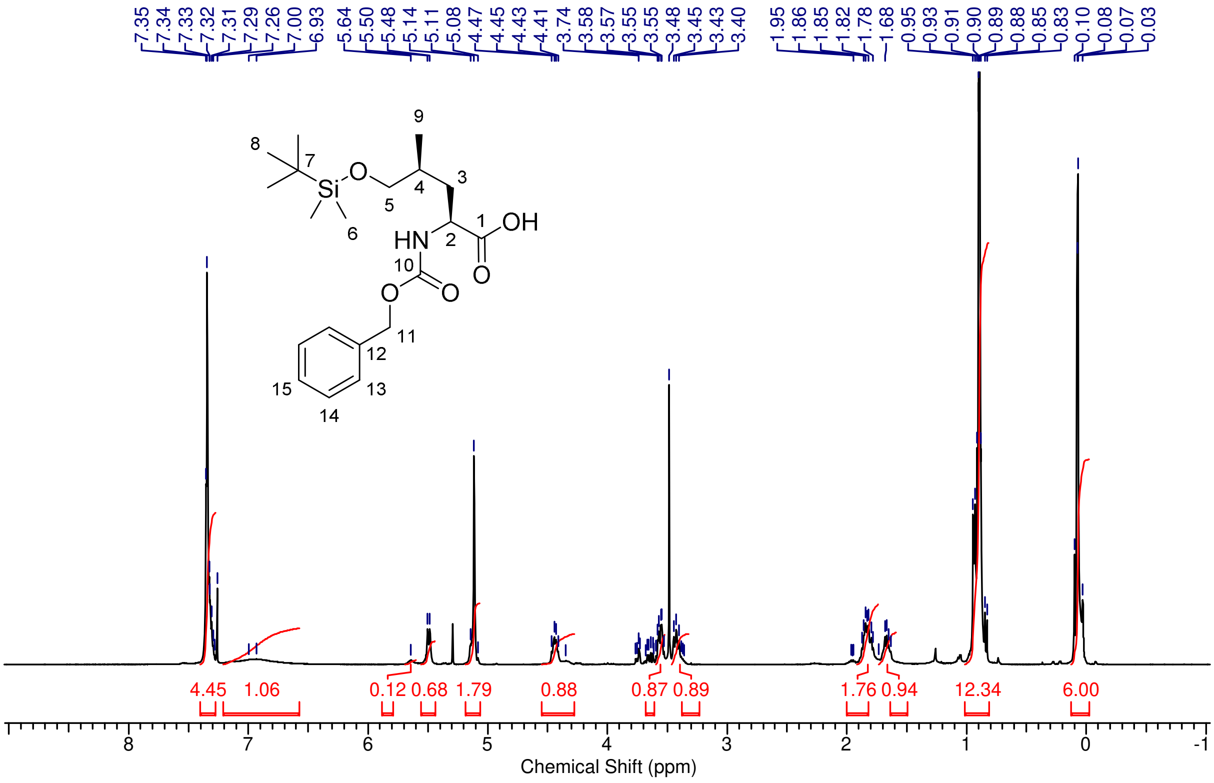

¹³C-NMR (100 MHz, $CDCl_3$):

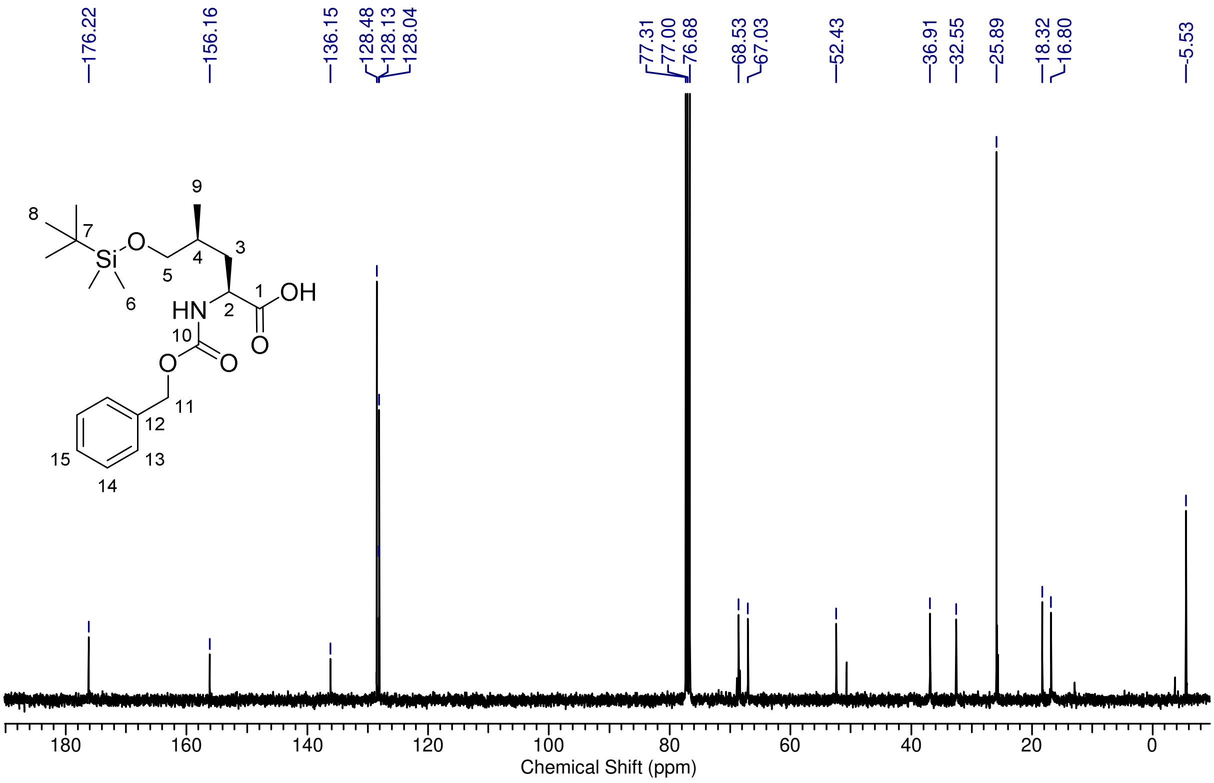

(2*S*,4*S*)-2-{[(Benzyloxy)carbonyl](methyl)amino}-5-[(*tert*-butyldimethylsilyl)oxy]-4-methylpentanoic acid (12)

^{1}H-NMR (400 MHz, $CDCl_3$):

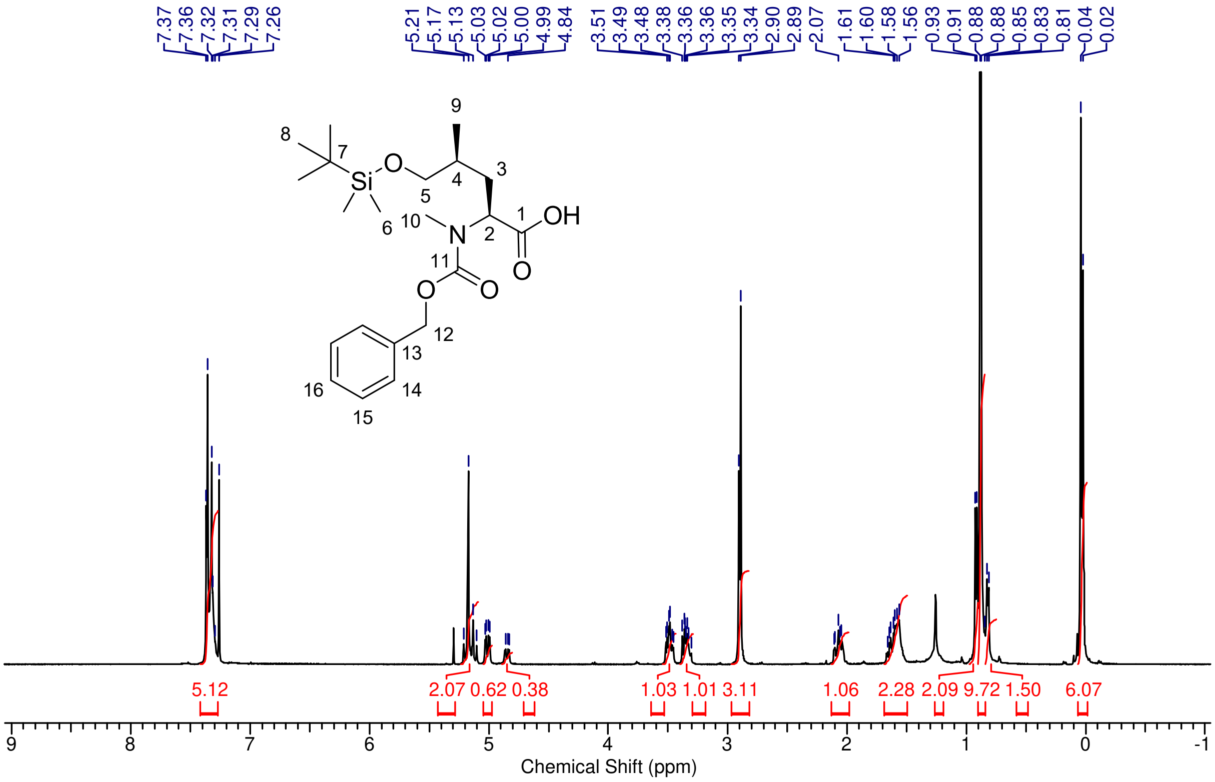

^{13}C-NMR (100 MHz, $CDCl_3$):

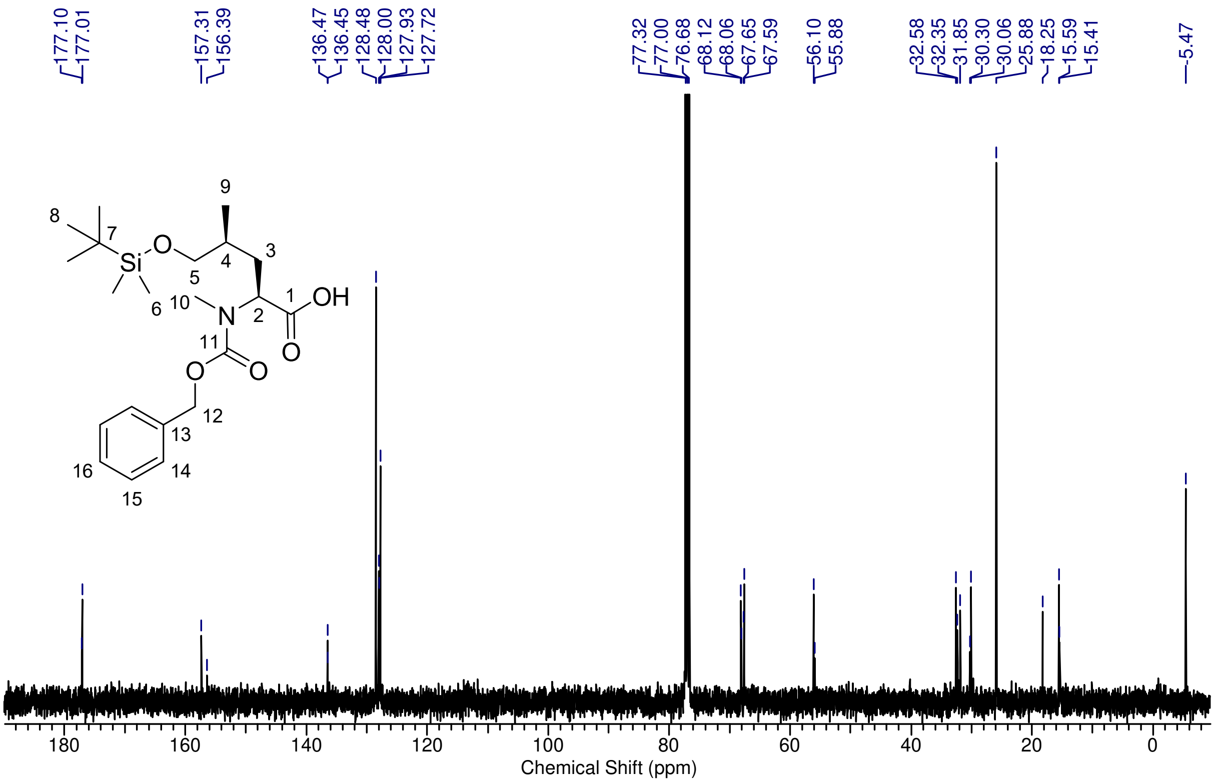

(*S*)-But-3-yn-2-yl [(allyloxy)carbonyl]glycinate (14)

^{1}H-NMR (400 MHz, $CDCl_3$):

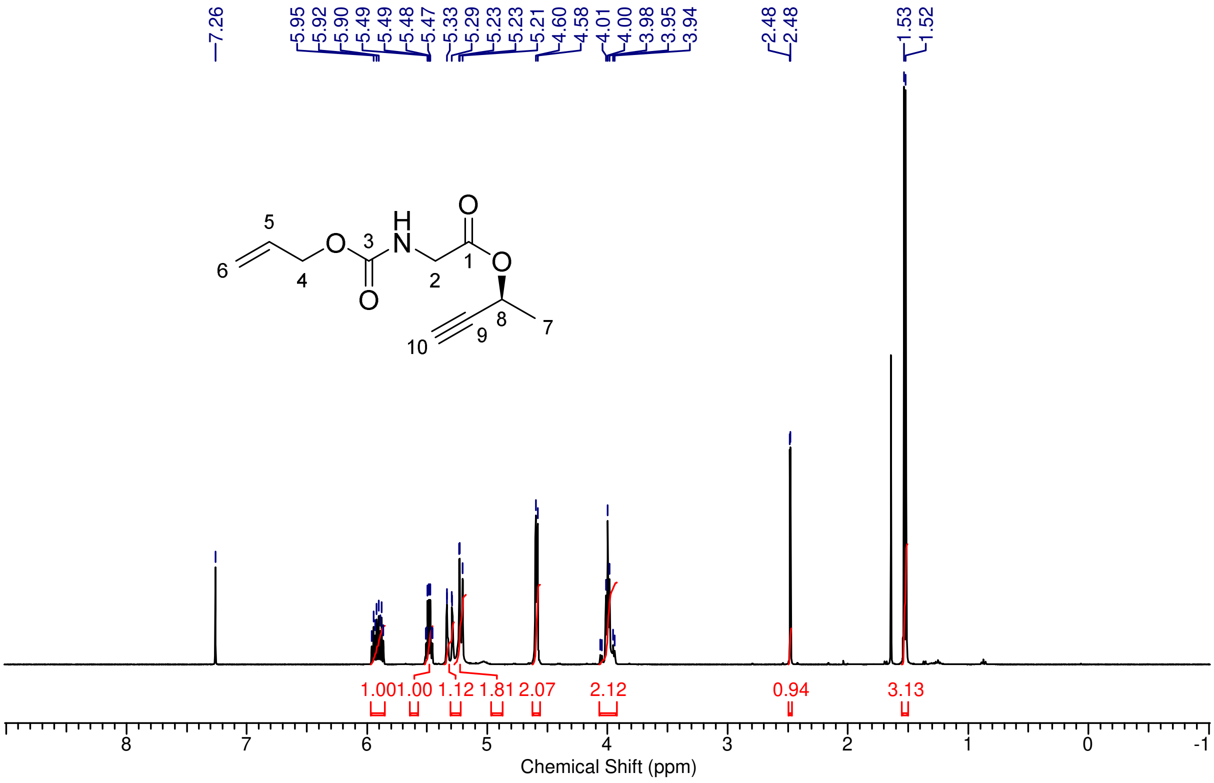

^{13}C-NMR (100 MHz, $CDCl_3$):

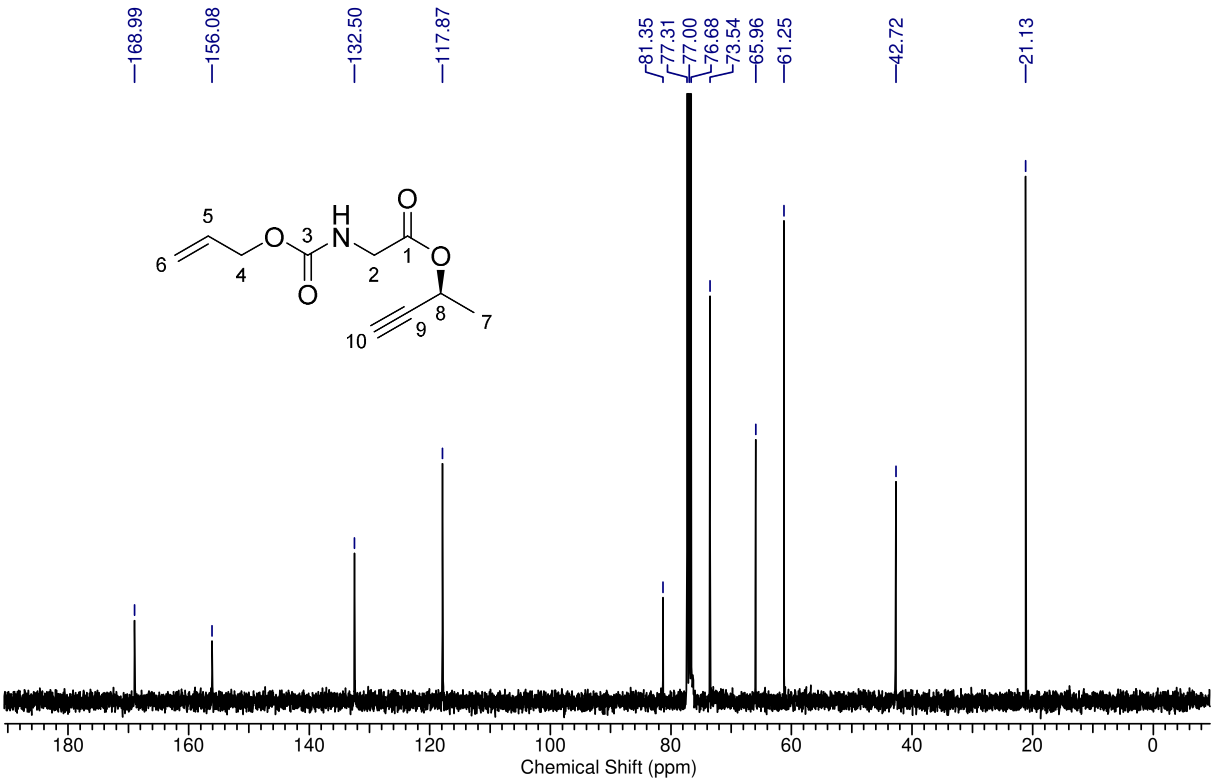

(*S*)-But-3-en-2-yl [(allyloxy)carbonyl]glycinate (15)

^{1}H-NMR (400 MHz, $CDCl_3$):

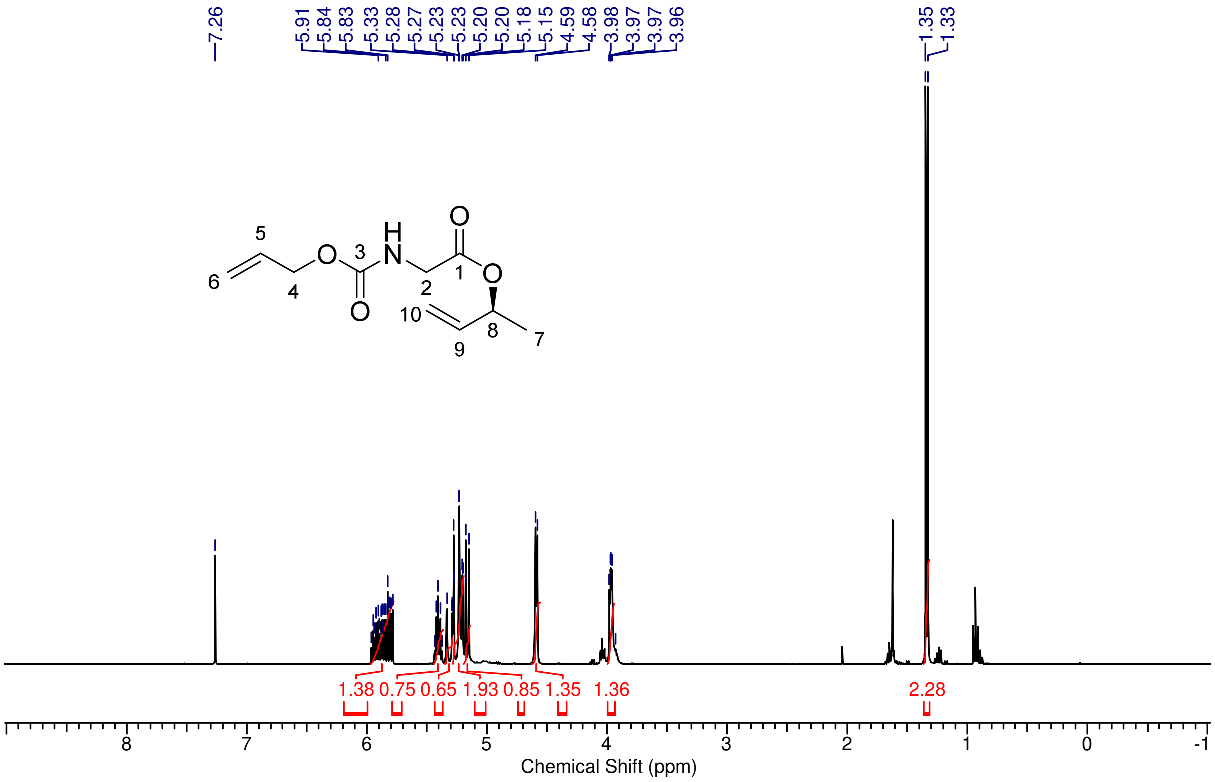

^{13}C-NMR (100 MHz, $CDCl_3$):

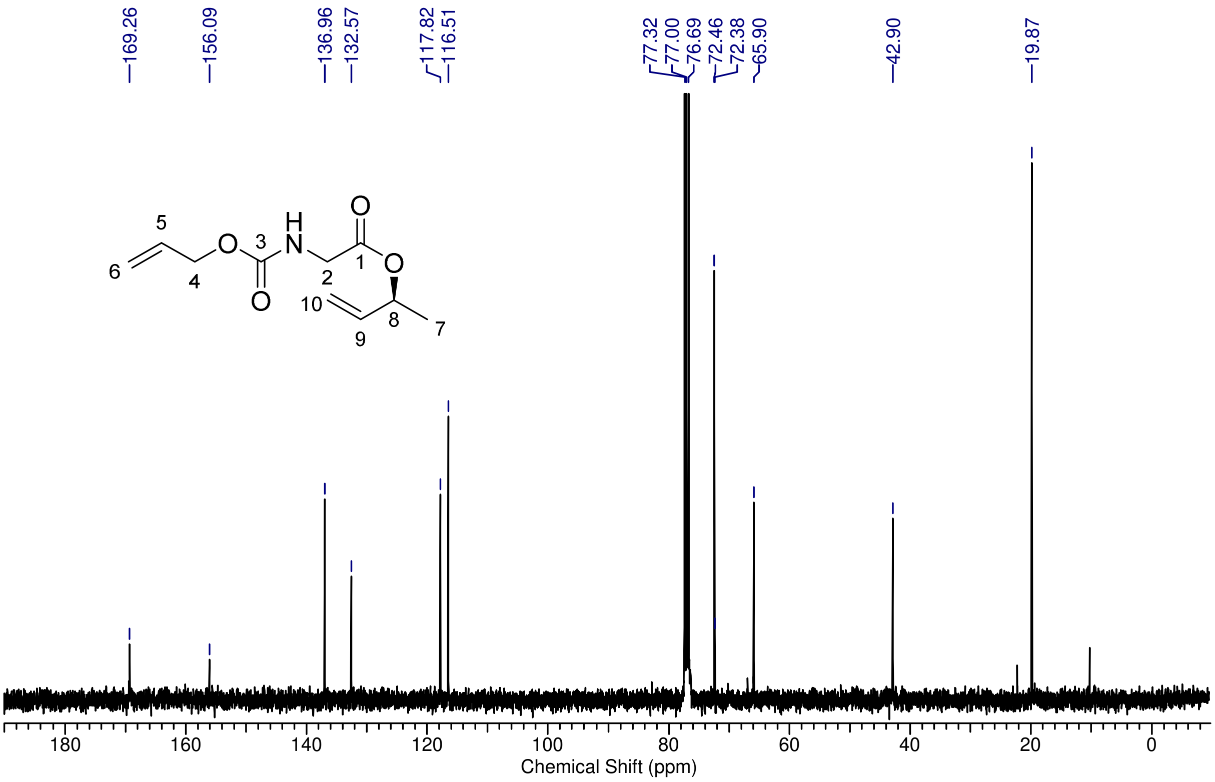

(*S*,*E*)-2-{[(Allyloxy)carbonyl]amino}hex-4-enoic acid (16)

^{1}H-NMR (400 MHz, $CDCl_3$):

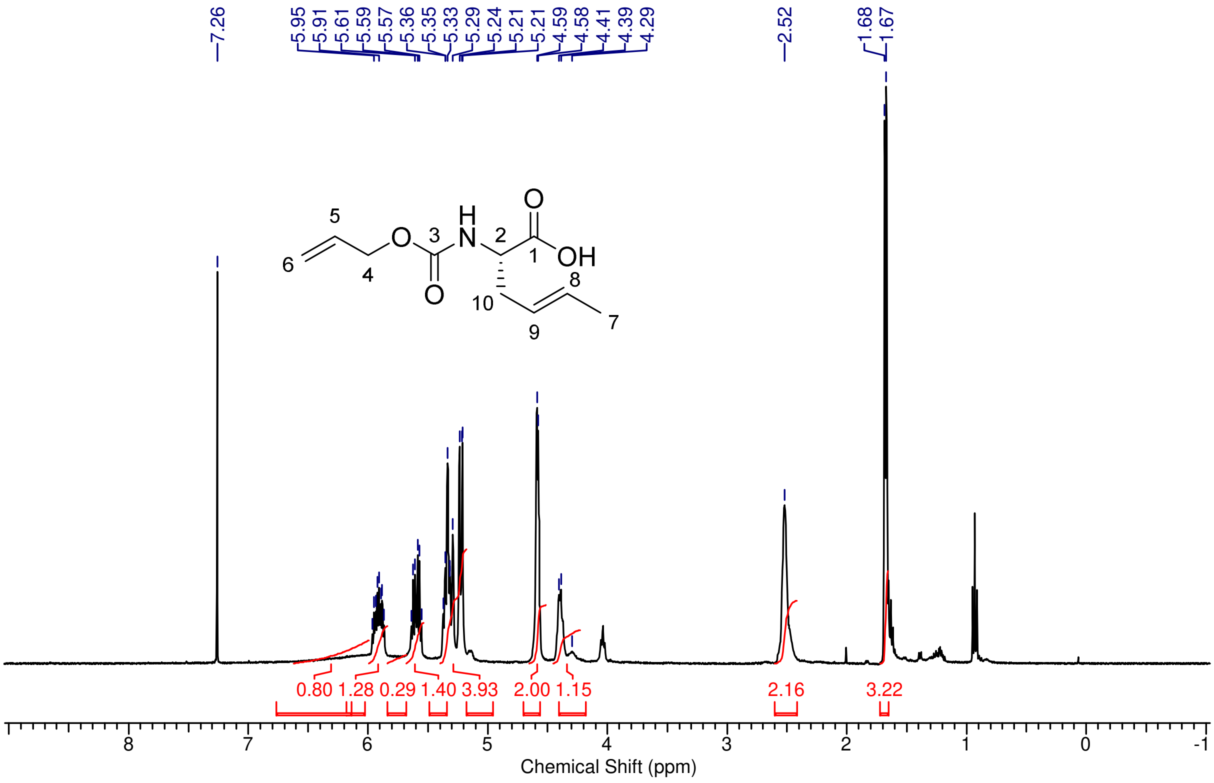

^{13}C-NMR (100 MHz, $CDCl_3$):

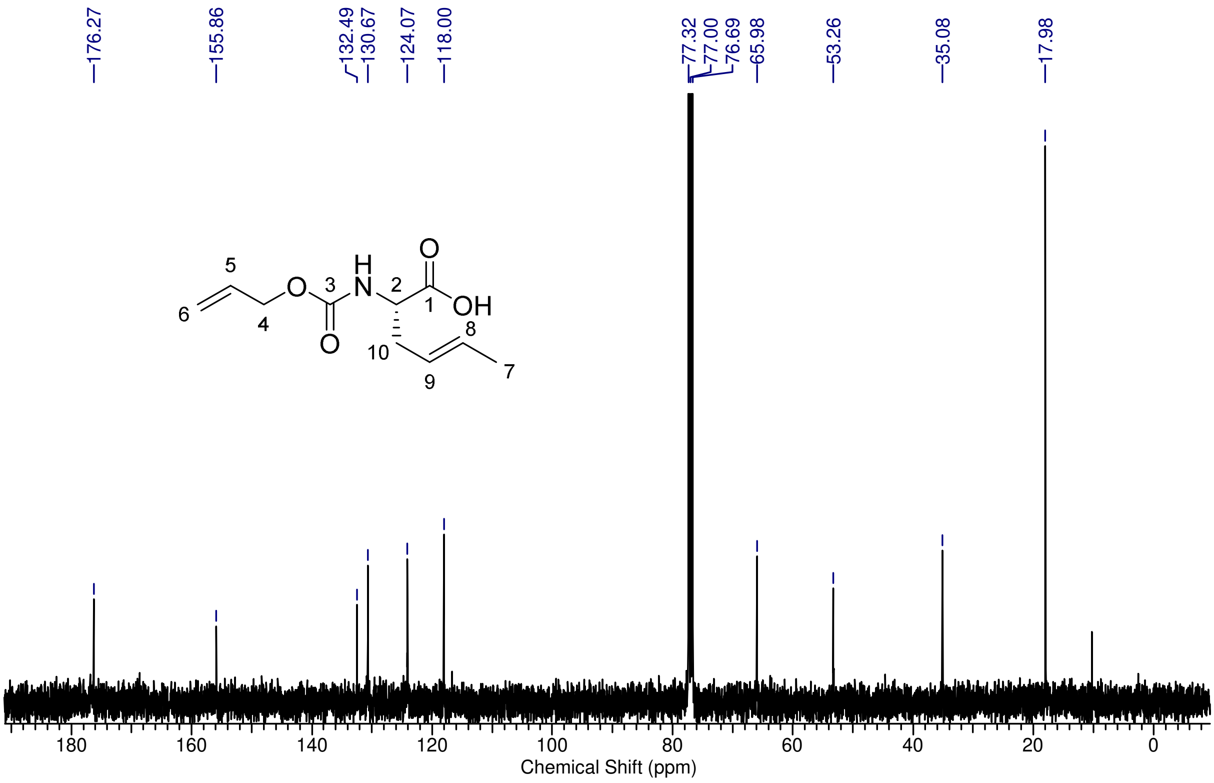

Methyl {[2*S*,4*S*]-2-({[benzyloxy]carbonyl}(methyl)amino)-5-[(*tert*-butyldimethyl-silyl)oxy]-4-methyl-pentanoyl}-L-leucinate (17)

^{1}H-NMR (400 MHz, $CDCl_3$):

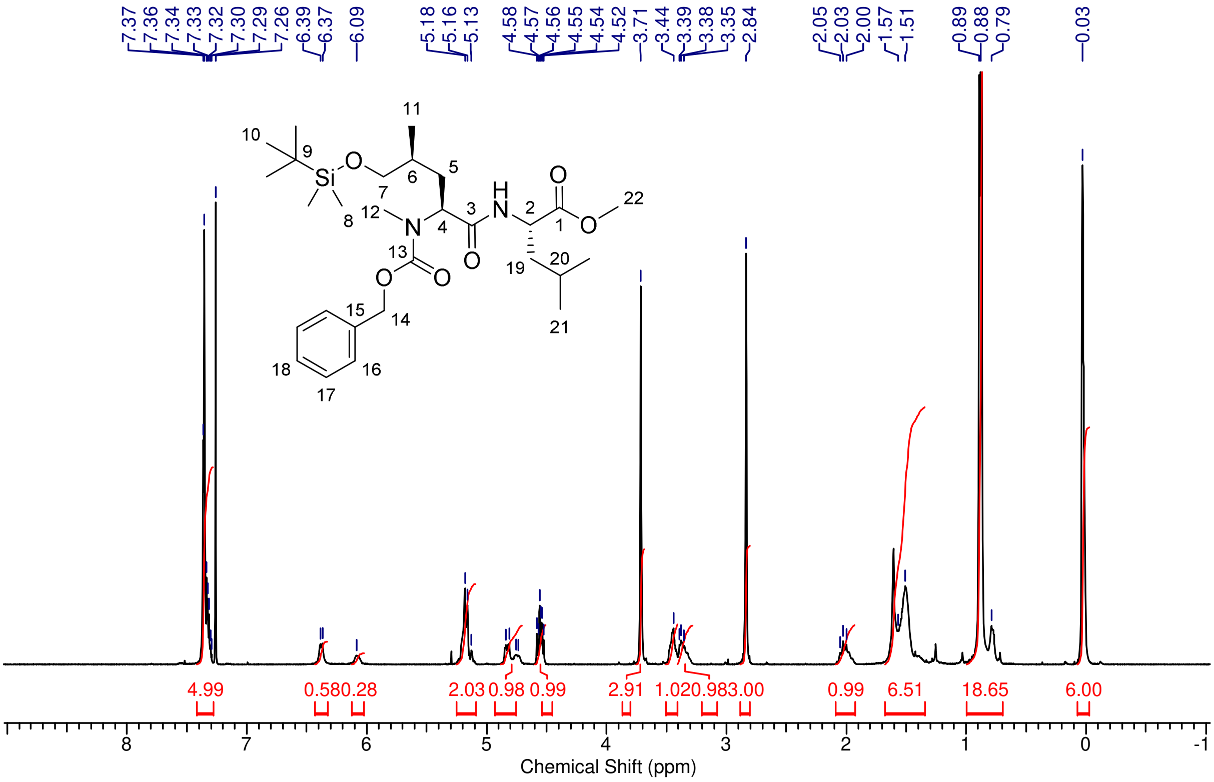

^{13}C-NMR (100 MHz, $CDCl_3$):

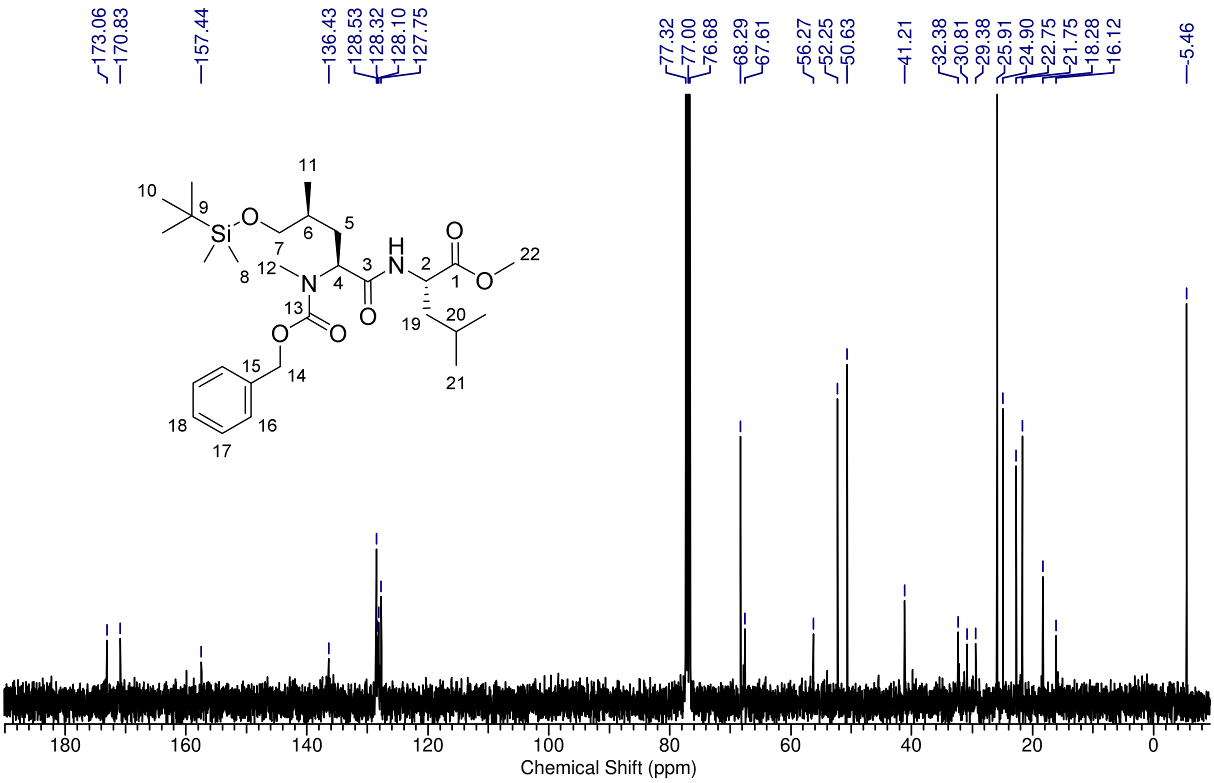

Methyl {[2*S*,4*S*]-2-({*S*}-2-{[(allyloxy)carbonyl]amino}-*N*-methylpropanamido)-5-[(*tert*-butyldimethylsilyl)oxy]-4-methylpentanoyl}-L-leucinate (18)

^{1}H-NMR (400 MHz, $CDCl_3$):

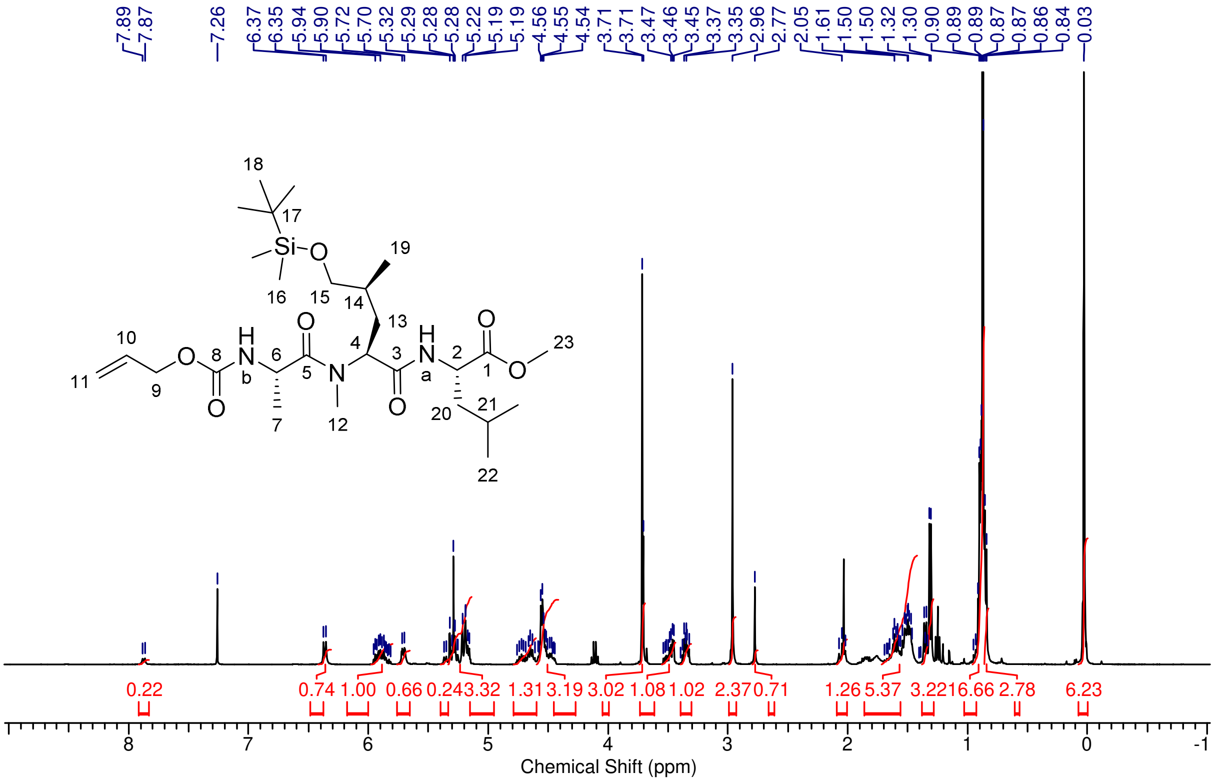

^{13}C-NMR (100 MHz, $CDCl_3$):

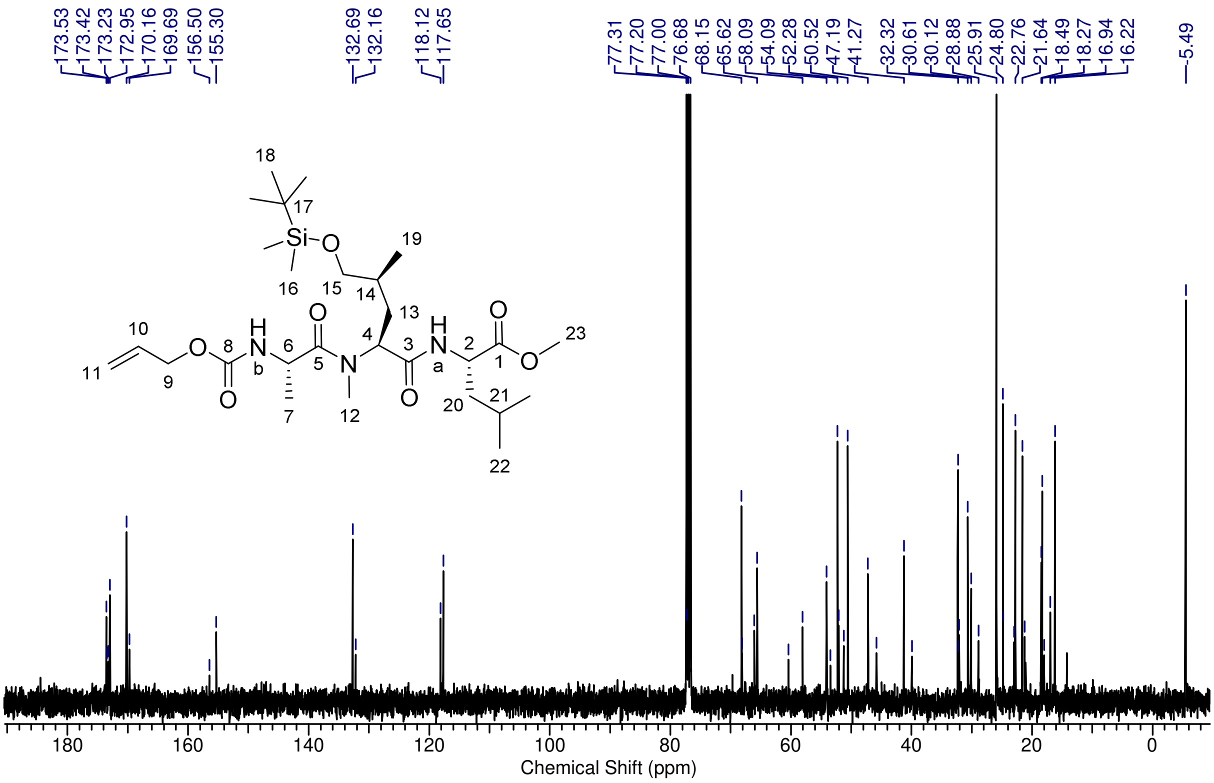

Methyl {[2*S*,4*S*]-2-({*S*}-2-[(*S*)-2-{[(allyloxy)carbonyl]amino}-3-{4-[(2-methoxy-ethoxy)methoxy]-3-nitrophenyl}propanamido]-*N*-methylpropanamido)-5-[(*tert*-butyldimethylsilyl)oxy]-4-methyl-pentanoyl}-L-leucinate (19)

^{1}**H-NMR** (400 MHz, $CDCl_3$):

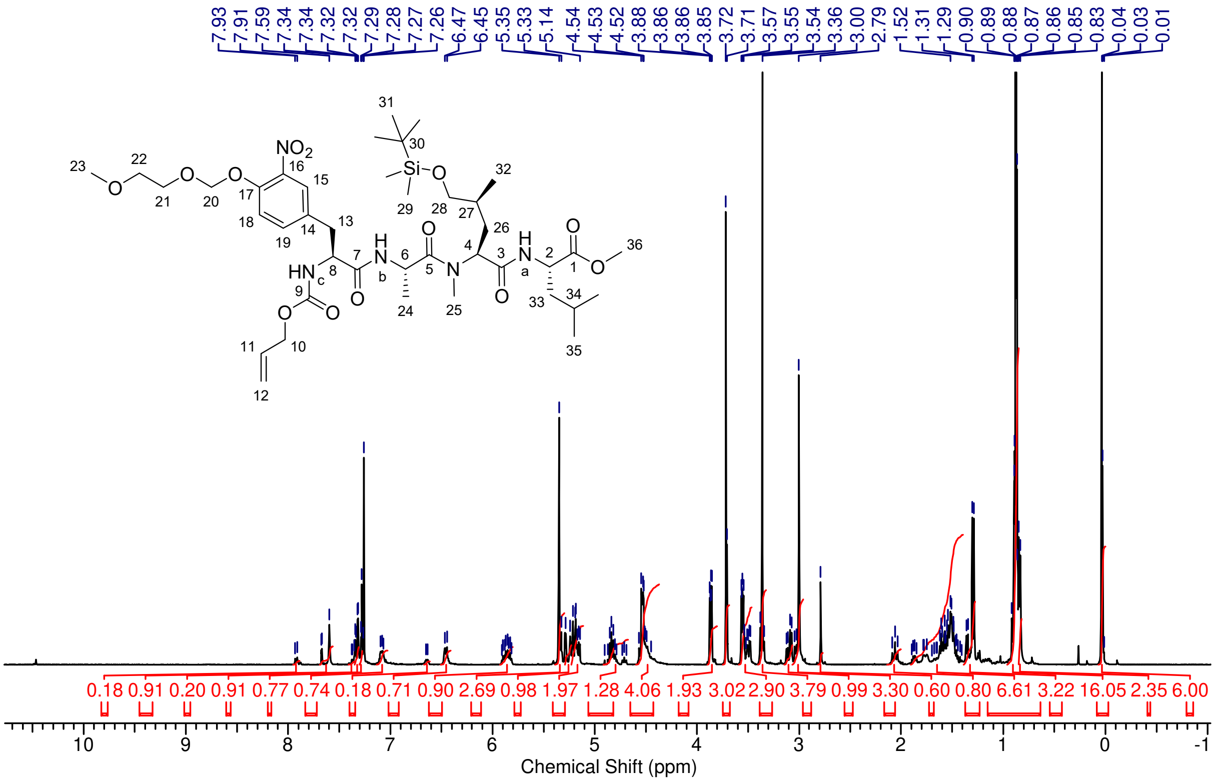

^{13}**C-NMR** (100 MHz, $CDCl_3$):

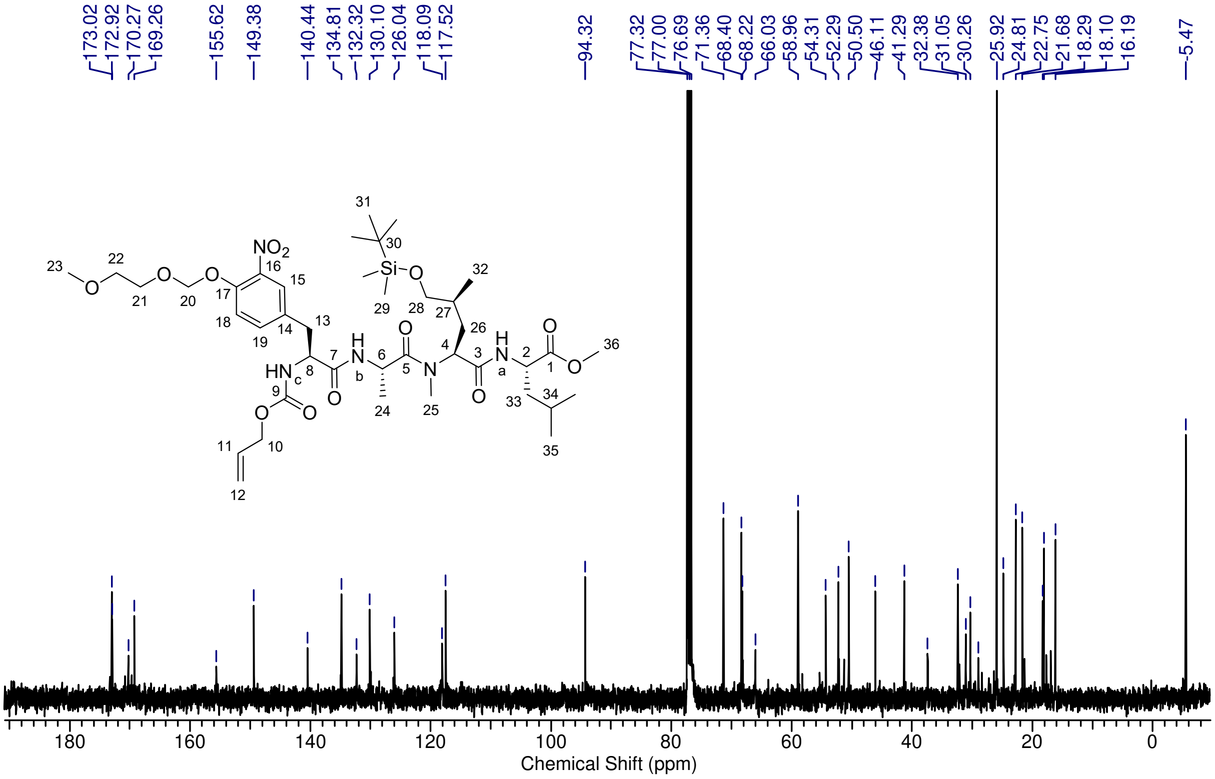

Methyl {[2*S*,4*S*]-2-({*S*}-2-[(*S*)-2-{[*S*]-2-({[allyloxy]carbonyl}(methyl)amino)-4-methylpentanamido}-3-{4-[(2-methoxyethoxy)methoxy]-3-nitrophenyl}propan-amido]-*N*-methylpropanamido)-5-[(*tert*-butyldimethylsilyl)oxy]-4-methyl-pentanoyl}-L-leucinate (20)

^{1}H-NMR (400 MHz, $CDCl_3$):

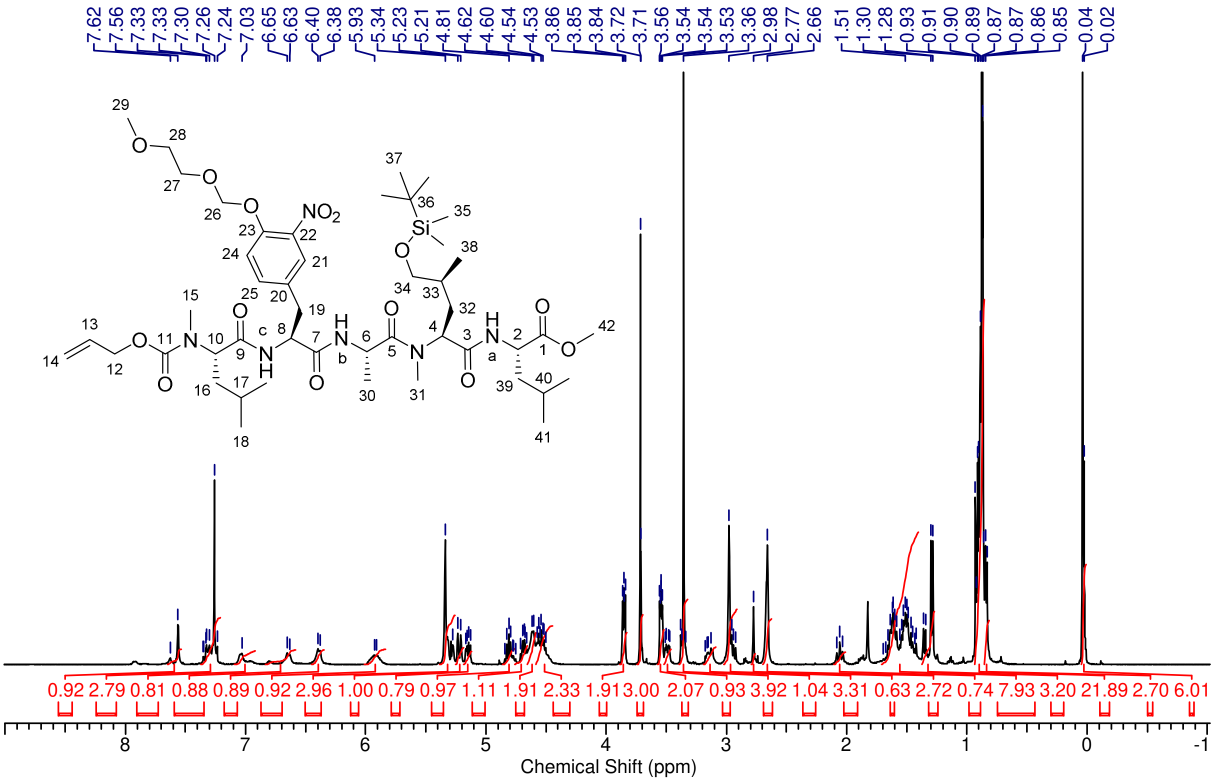

^{13}C-NMR (100 MHz, $CDCl_3$):

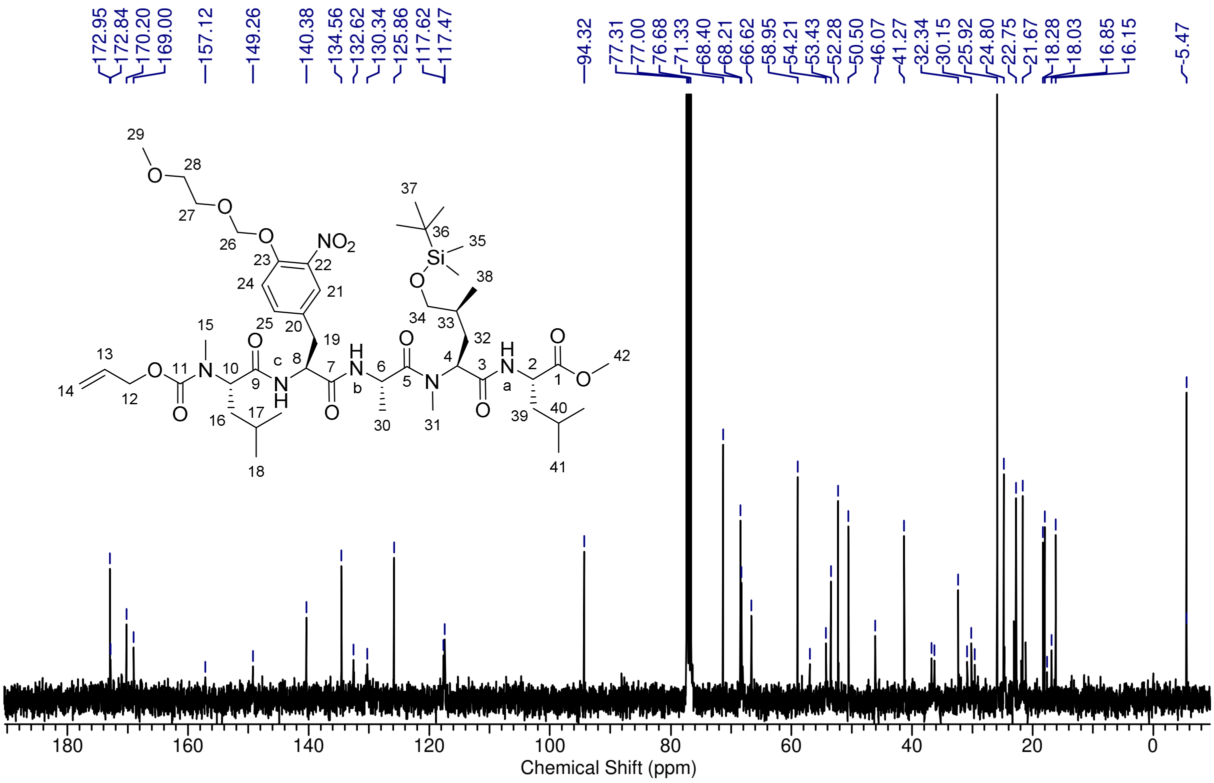

Methyl {[2*S*,4*S*]-2-({*S*}-2-[(*S*)-2-{[*S*]-2-({*S*}-2-[({allyloxy}carbonyl)amino]-*N*-methyl-3-[1-methyl-1*H*-indol-3-yl]propanamido)-4-methylpentanamido}-3-(4-{[2-methoxyethoxy]methoxy}-3-nitrophenyl)-propanamido]-*N*-methylpropanamido)-5-[(*tert*-butyldimethylsilyl)oxy]-4-methylpentanoyl}-L-leucinate (21)

^{1}H-NMR (400 MHz, $CDCl_3$):

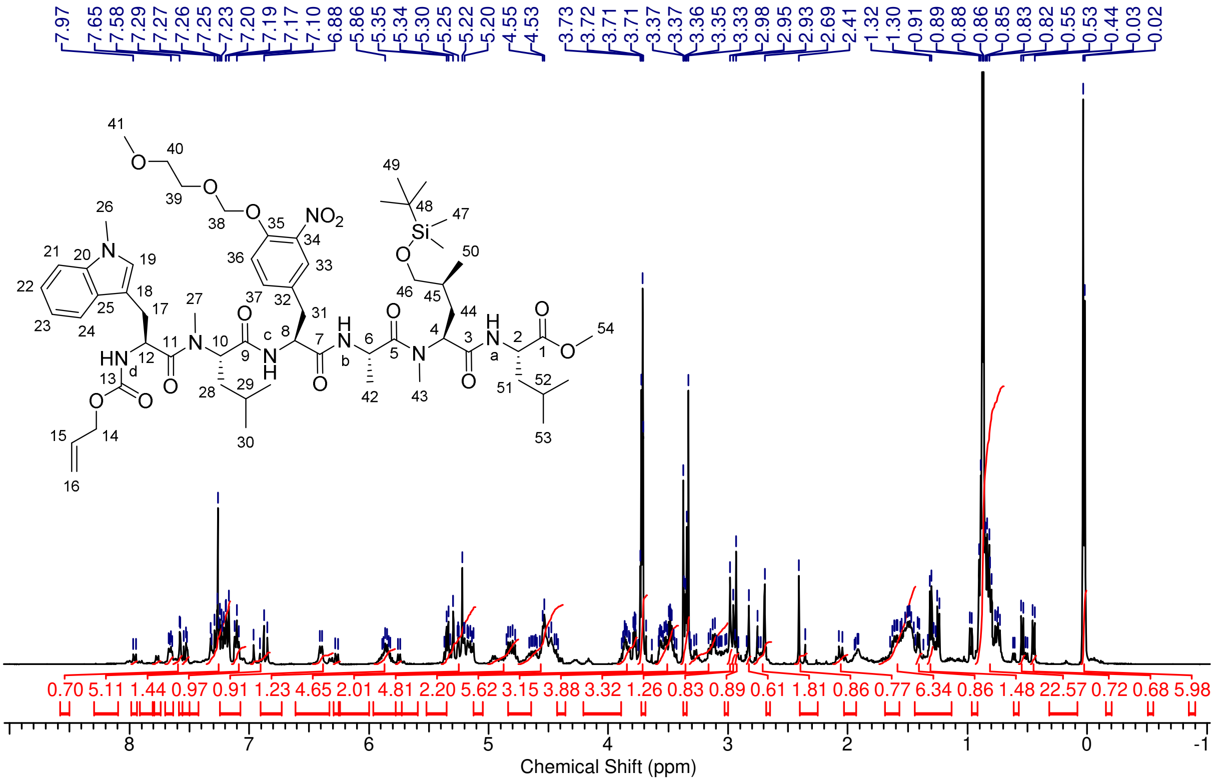

^{13}C-NMR (100 MHz, $CDCl_3$):

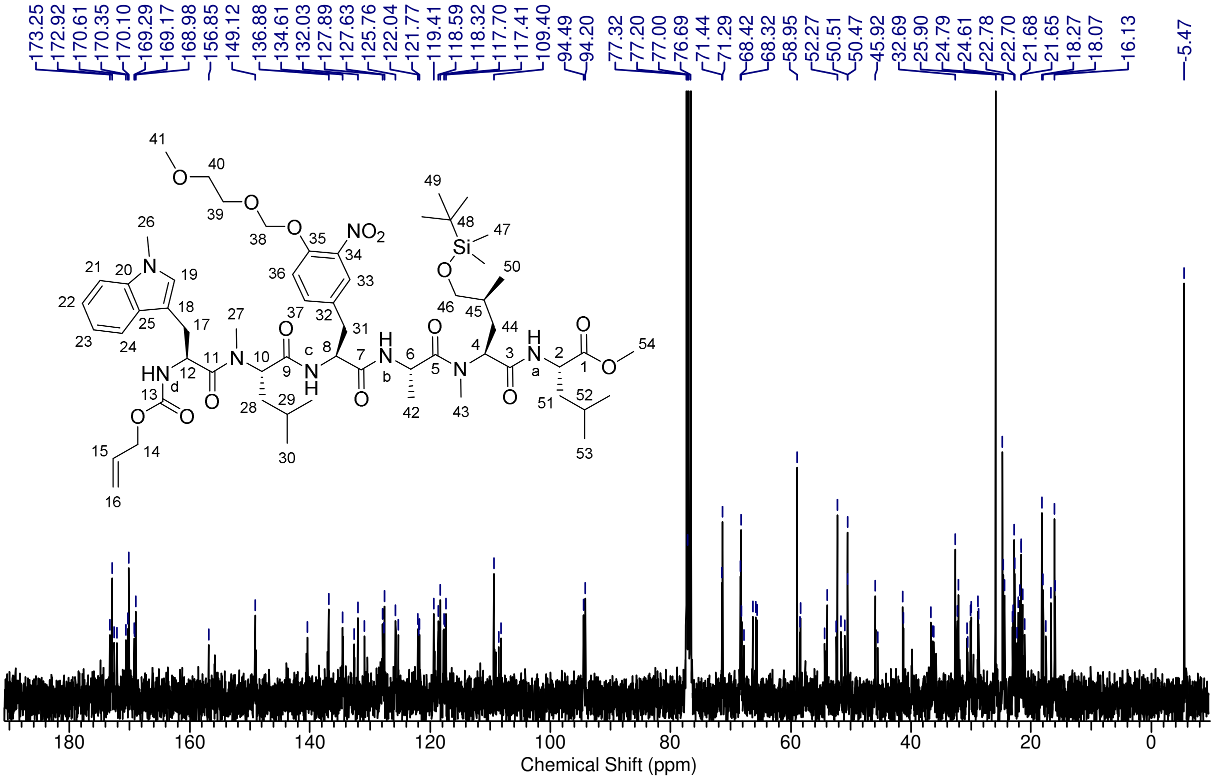

Methyl {[2*S*,4*S*]-2-({*S*}-2-[(*S*)-2-{[*S*]-2-({*S*}-2-[(*S*,*E*)-2-{[(allyloxy)carbonyl]amino}-hex-4-enamido]-*N*-methyl-3-{1-methyl-1*H*-indol-3-yl}propanamido)-4-methyl-pentanamido}-3-[4-({2-methoxyethoxy}-methoxy)-3-nitrophenyl]propanamido]-*N*-methylpropanamido)-5-[(*tert*-butyldimethylsilyl)oxy]-4-methylpentanoyl}-L-leucinate (22)

^{1}H-NMR (500 MHz, $CDCl_3$):

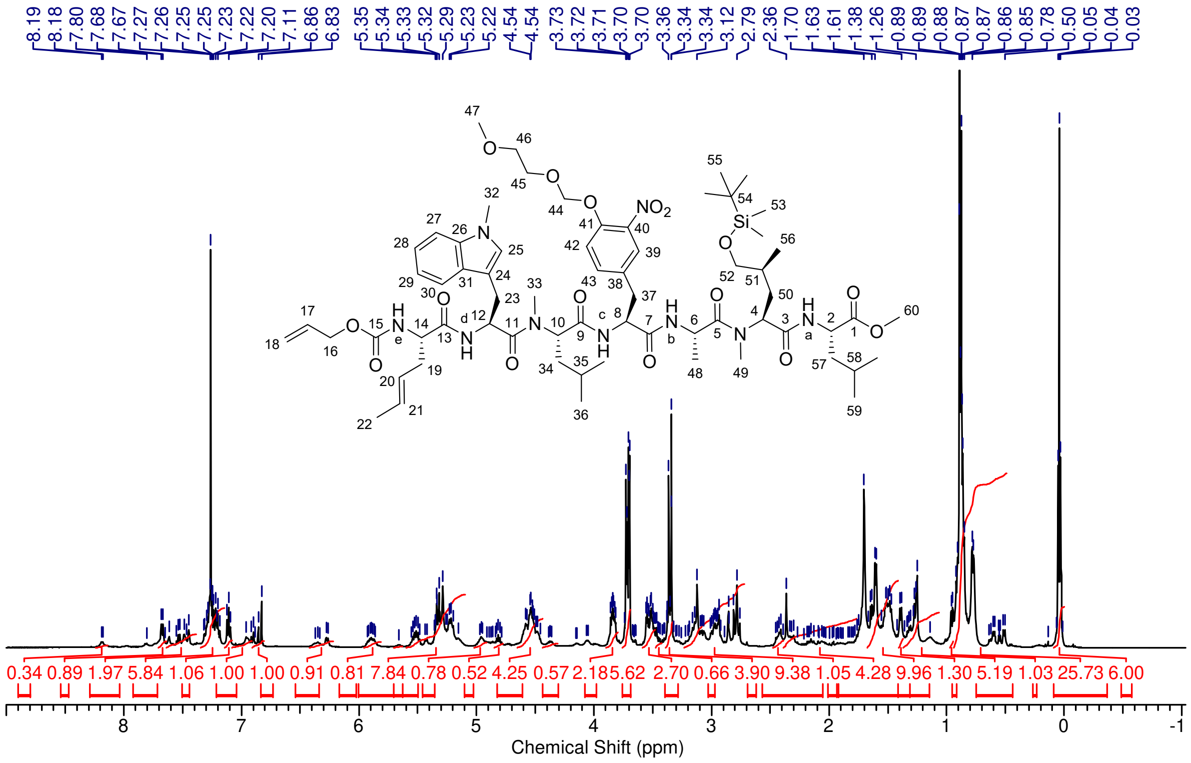

^{13}C-NMR (125 MHz, $CDCl_3$):

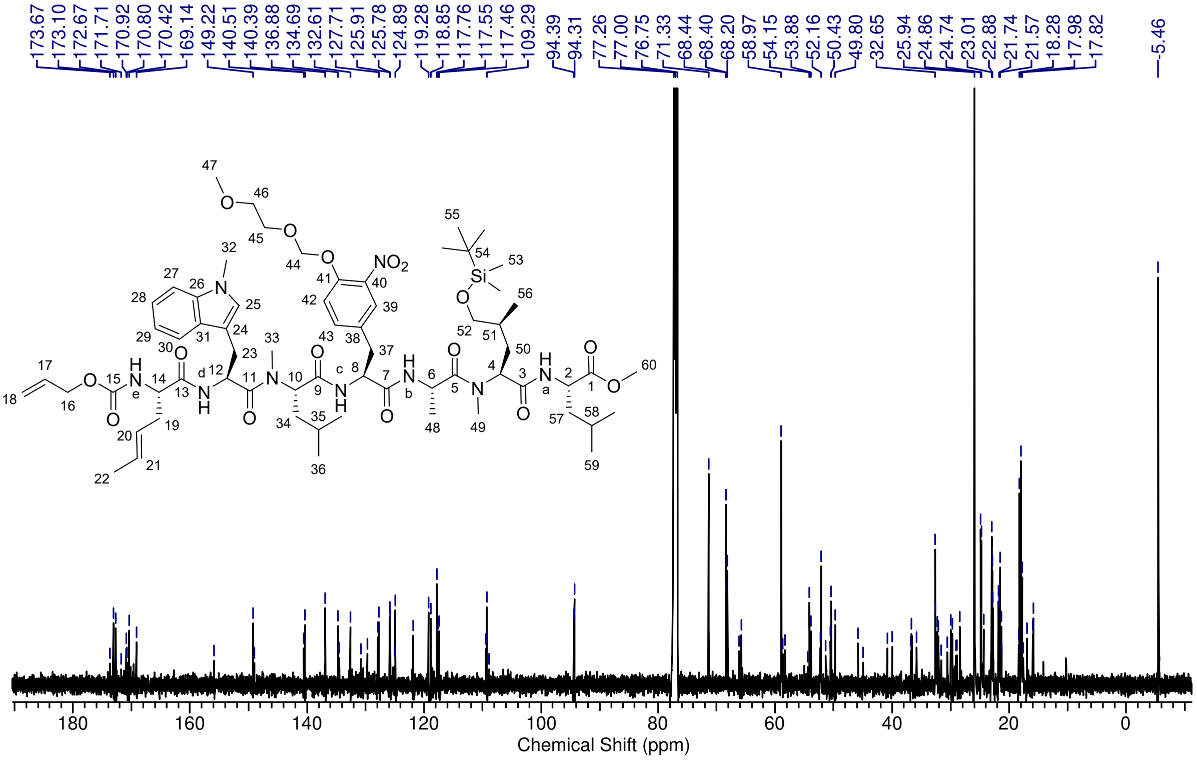

(3*S*,6*S*,9*S*,12*S*,15*S*,18*S*,21*S*)-15-[(*E*)-But-2-en-1-yl]-21-[(*S*)-3-{(*tert*-butyldimethyl-silyl)oxy}-2-methyl-propyl]-9,18-diisobutyl-6-[4-({2-methoxyethoxy}methoxy)-3-nitrobenzyl]-1,3,10-trimethyl-12-[(1-methyl-1*H*-indol-3-yl)methyl]-1,4,7,10,13,16,19-heptaazacyclohenicosane-2,5,8,11,14,17,20-heptaone (23)

^{1}H-NMR (400 MHz, $(CD_3)_2SO$):

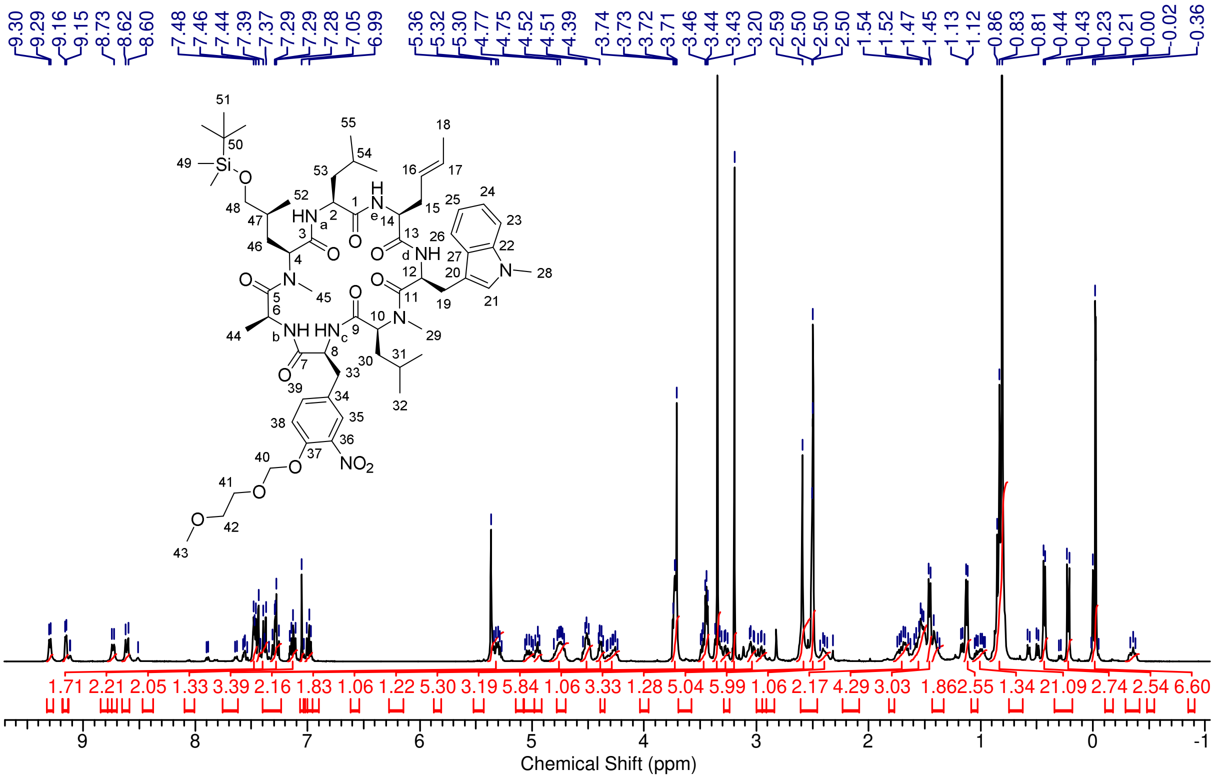

^{13}C-NMR (100 MHz, $(CD_3)_2SO$):

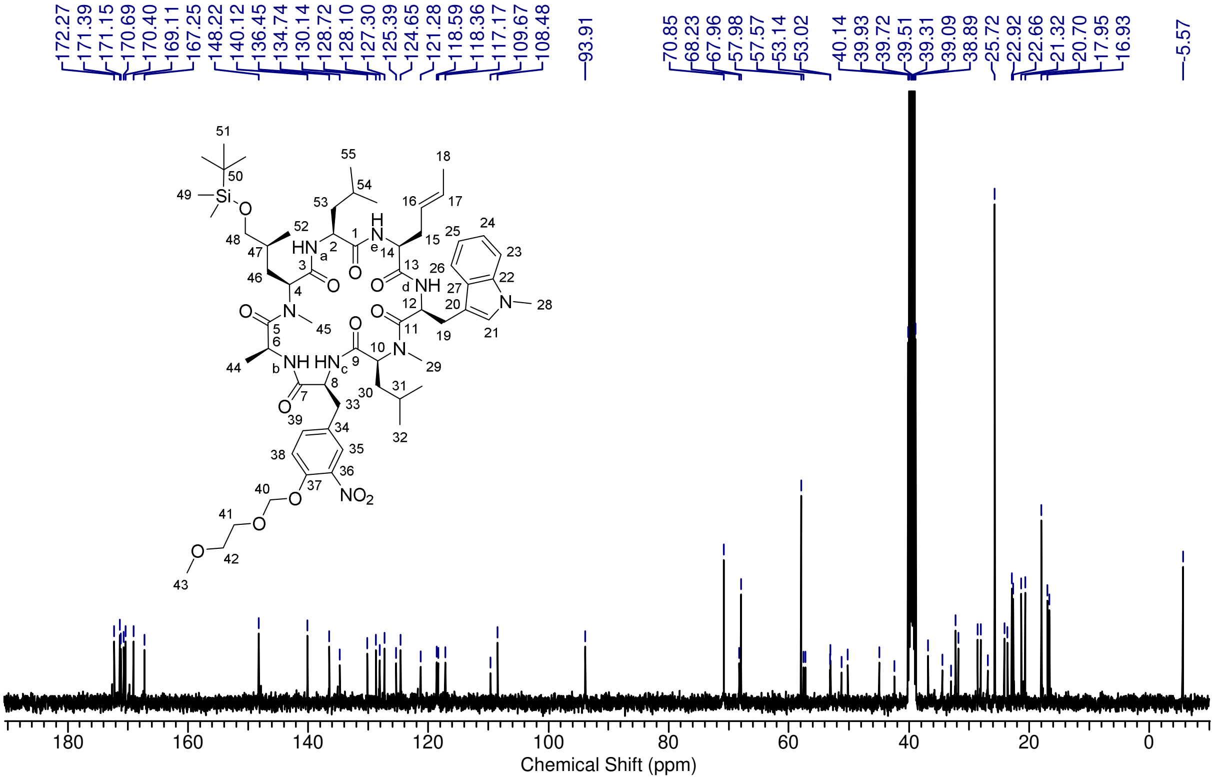

(3*S*,6*S*,9*S*,12*S*,15*S*,18*S*,21*S*)-15-[(*E*)-But-2-en-1-yl]-21-[(*S*)-3-hydroxy-2-methyl-propyl]-6-(4-hydroxy-3-nitrobenzyl)-9,18-diisobutyl-1,3,10-trimethyl-12-[(1-methyl-1*H*-indol-3-yl)methyl]-1,4,7,10,13,16,19-heptaazacyclohenicosane-2,5,8,11,14,17,20-heptaone (24)

[1]**H-NMR** (500 MHz, $(CD_3)_2SO$):

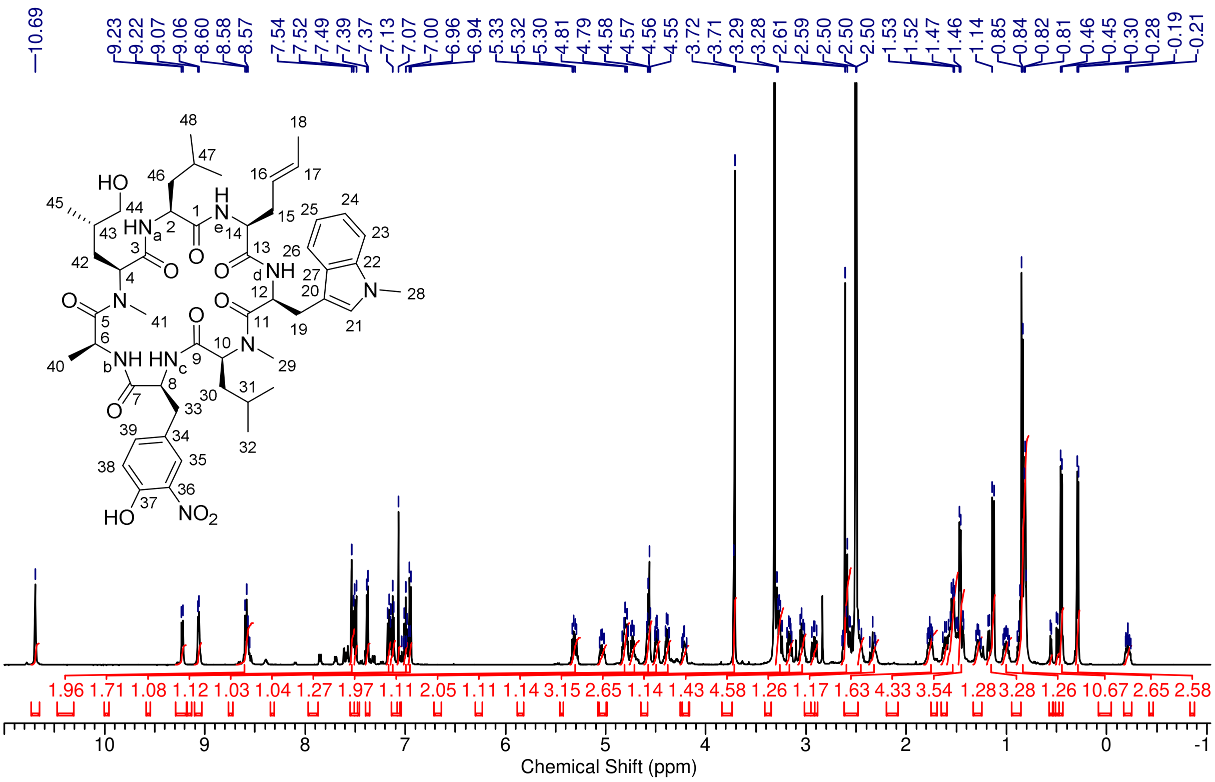

[13]**C-NMR** (125 MHz, $(CD_3)_2SO$):

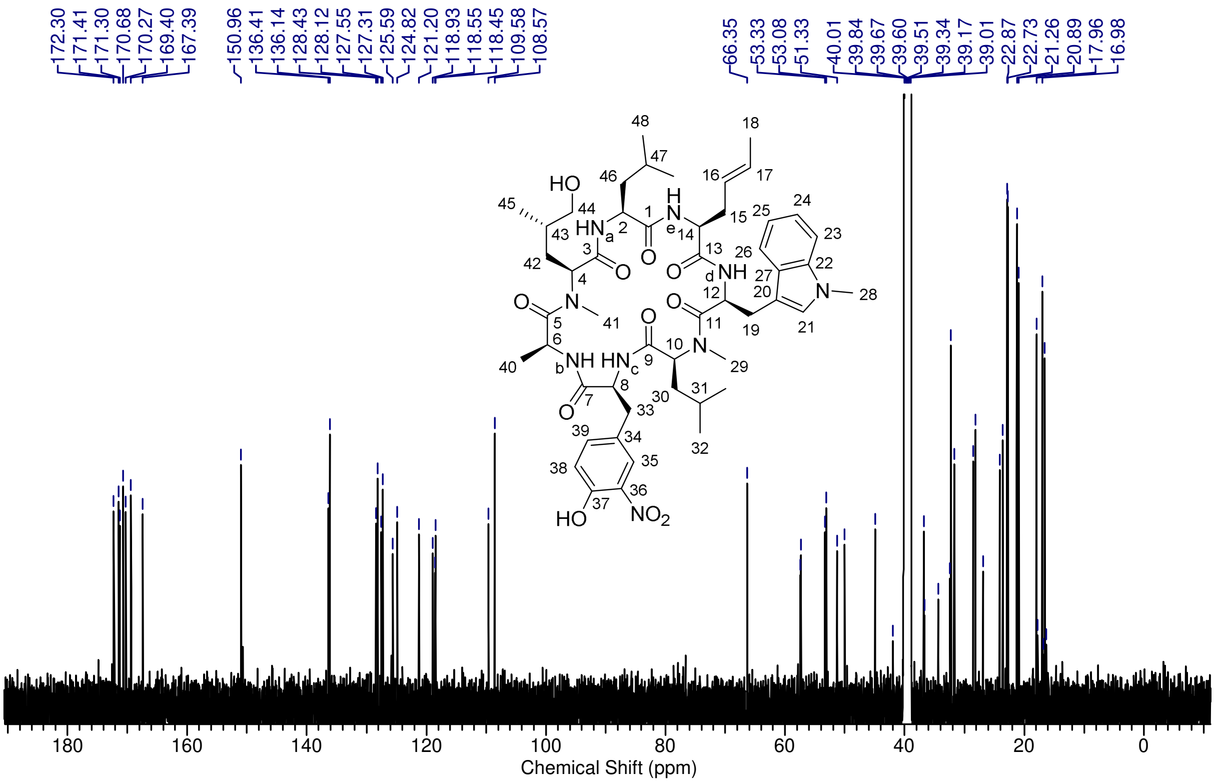

(2*S*,5*S*,8*S*,11*S*,14*S*,17*S*,20*S*,22*S*,23*R*)-5-[(*E*)-But-2-en-1-yl]-23-hydroxy-14-(4-hydroxy-3-nitrobenzyl)-2,11-diisobutyl-10,17,19,22-tetramethyl-8-[(1-methyl-1*H*-indol-3-yl)methyl]-1,4,7,10,13,16,19-heptaazabicyclo[18.3.1]tetracosane-3,6,9,12,15,18,24-heptaone (26)

^{1}H-NMR (500 MHz, CD_3OD):

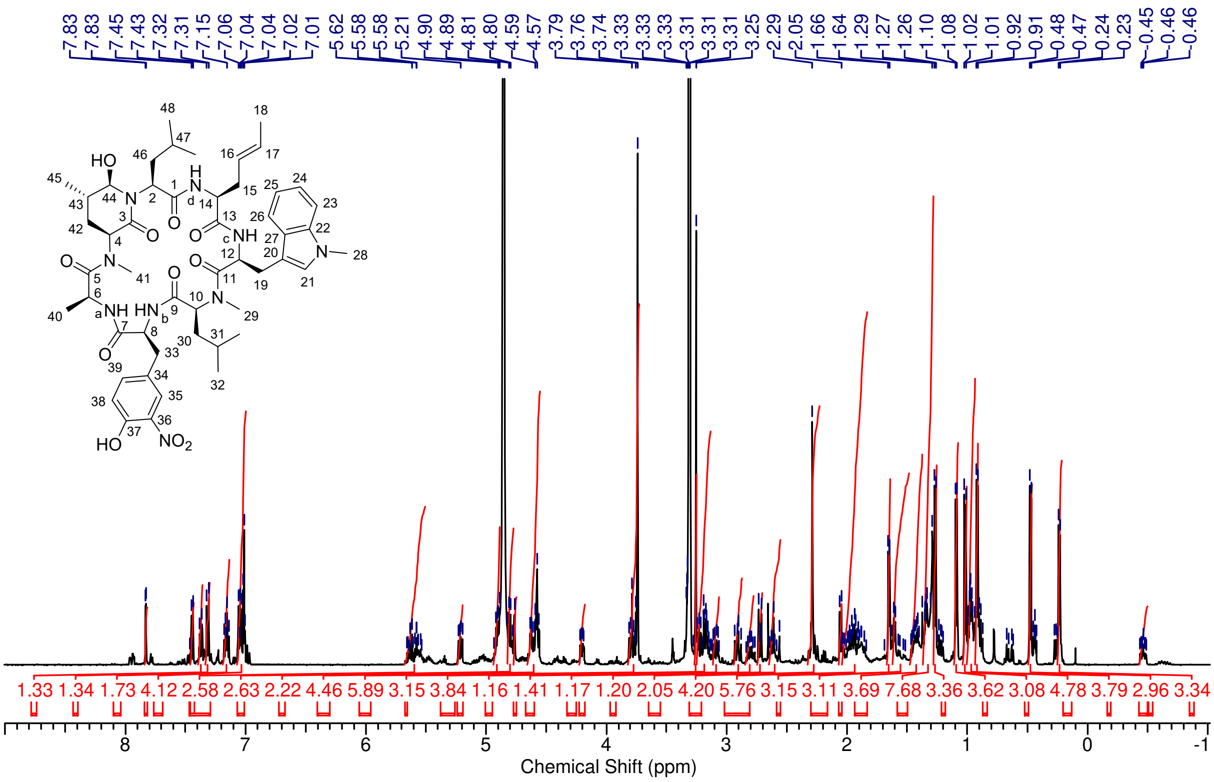

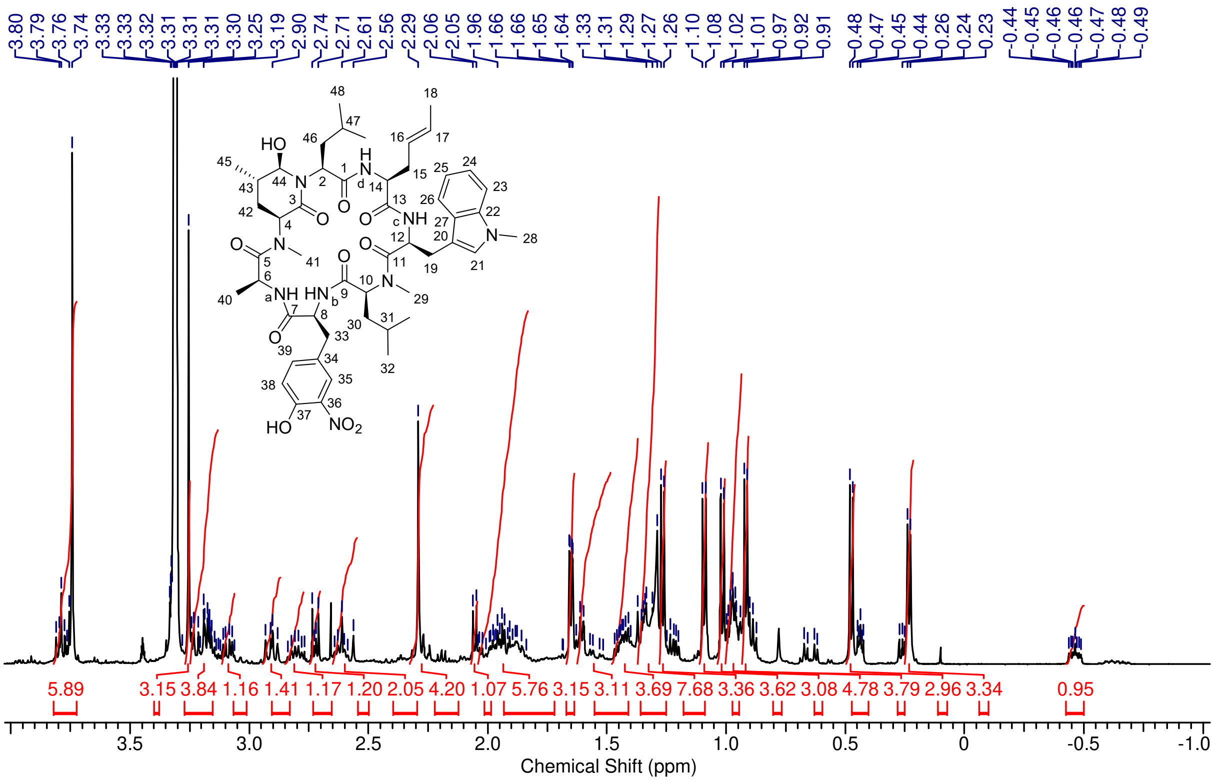

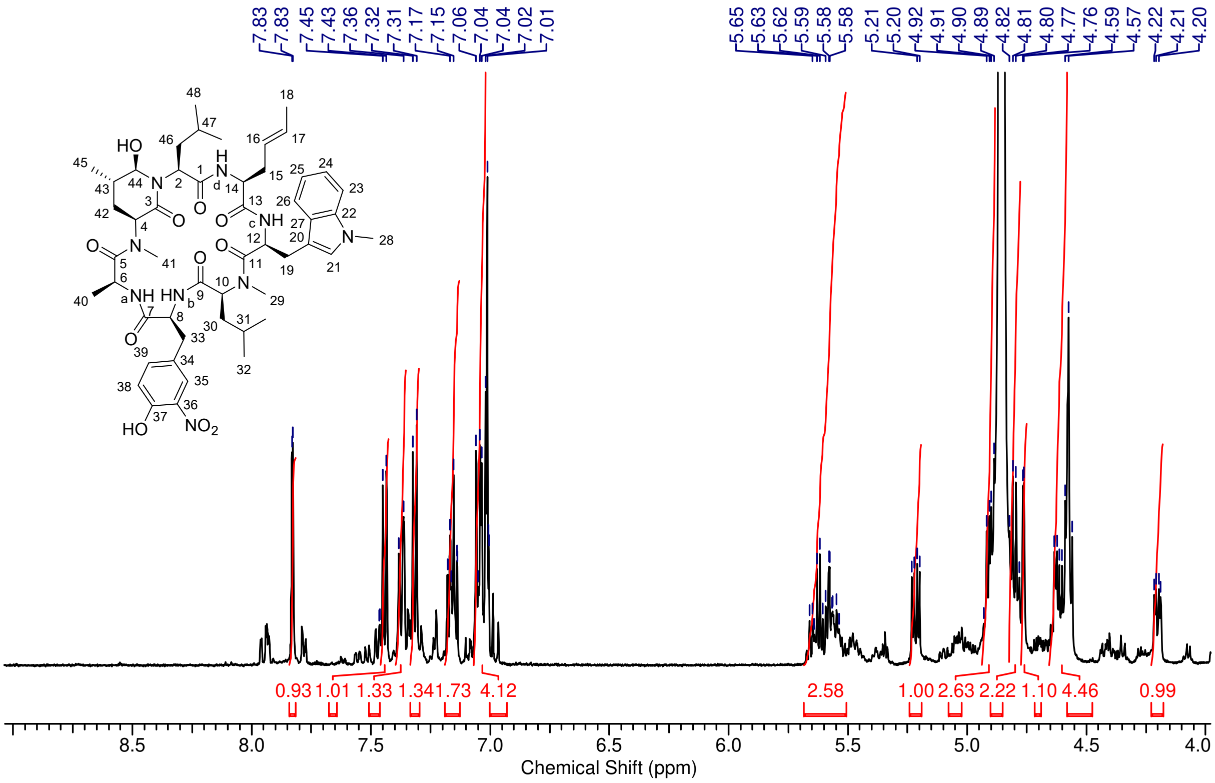

¹³C-NMR (125 MHz, CD_3OD):

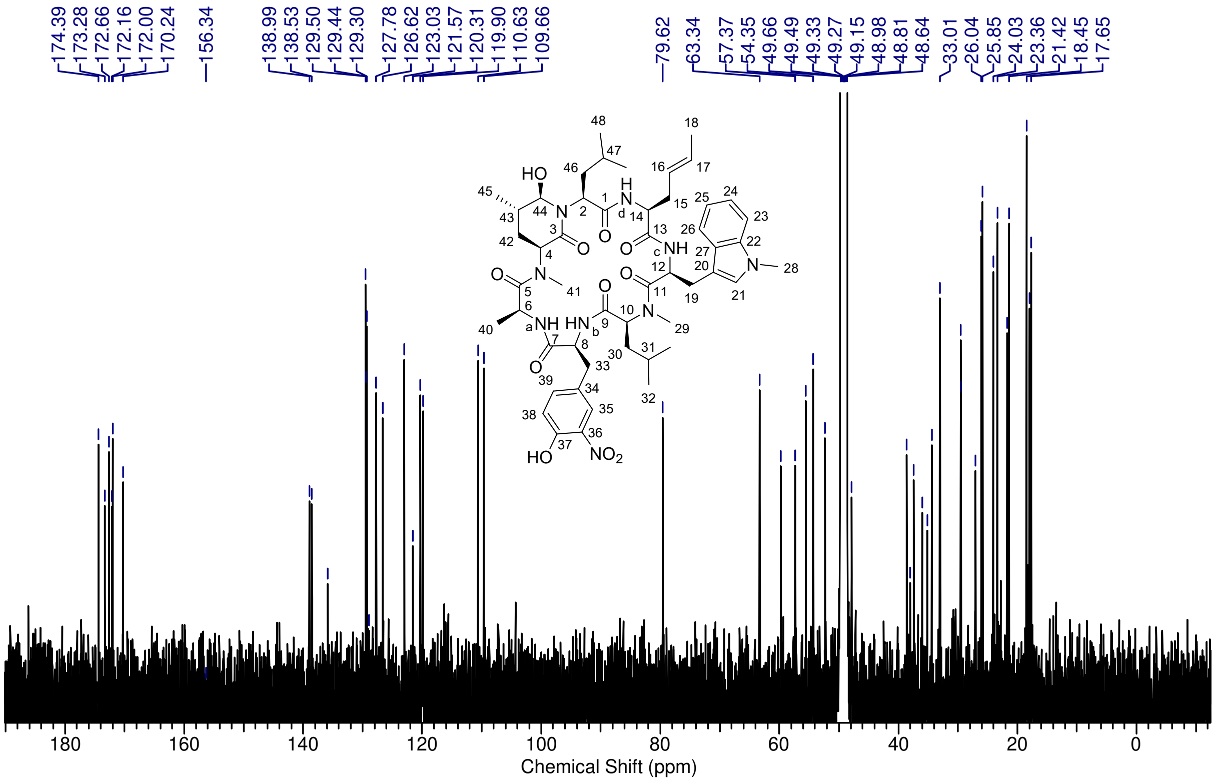

(*S*)-3-[(2*S*,5*S*,8*S*,11*S*,14*S*,17*S*,20*S*)-8-{(*E*)-But-2-en-1-yl}-17-[4-hydroxy-3-nitro-benzyl]-5,14-diisobutyl-1,13,20-trimethyl-11-{(1-methyl-1*H*-indol-3-yl)-methyl}-3,6,9,12,15,18,21-heptaoxo-1,4,7,10,13,16,19-heptaazacyclo-henicosan-2-yl]-2-methylpropanoic acid (27)

^{1}H-NMR (500 MHz, CD_3OD):

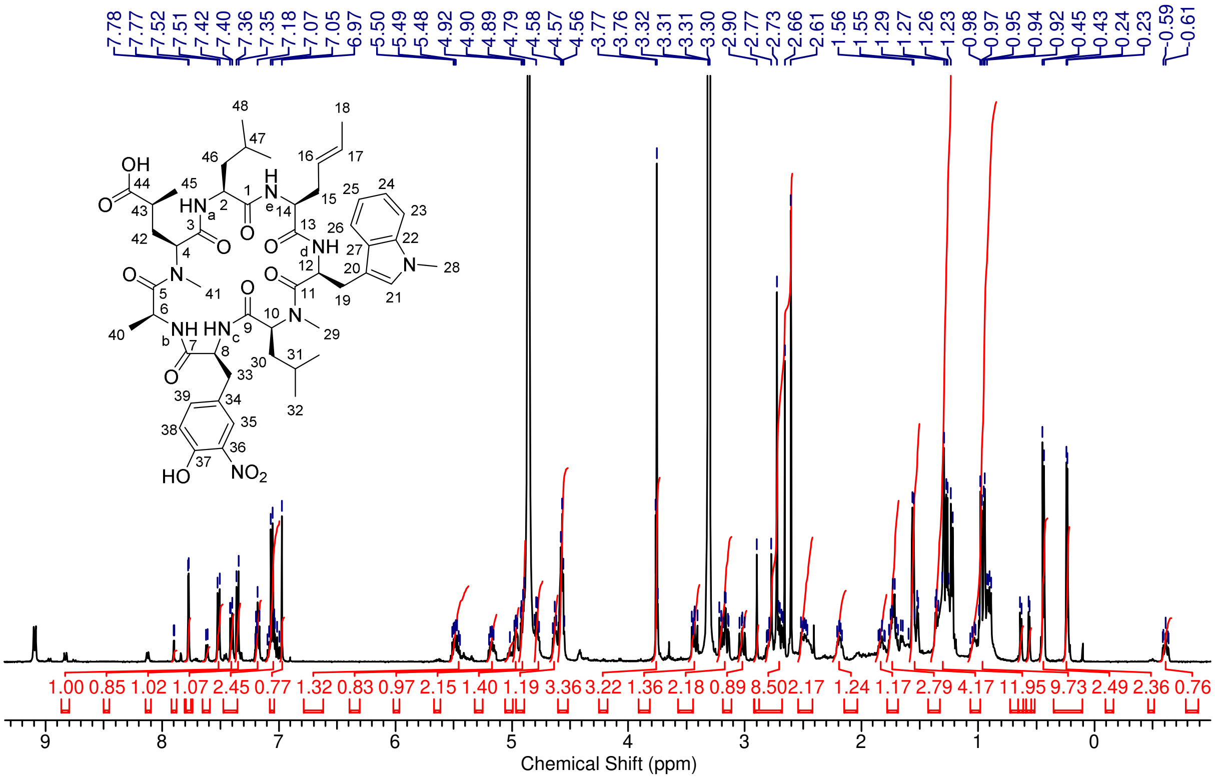

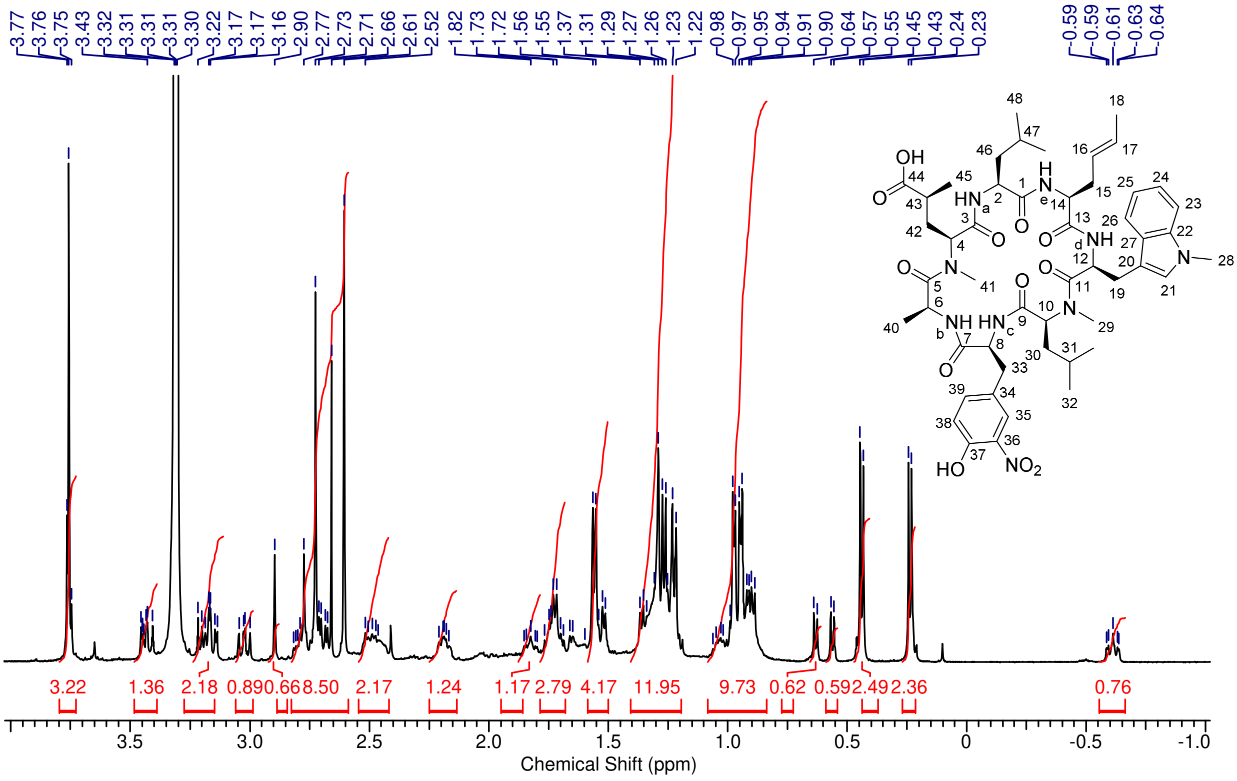

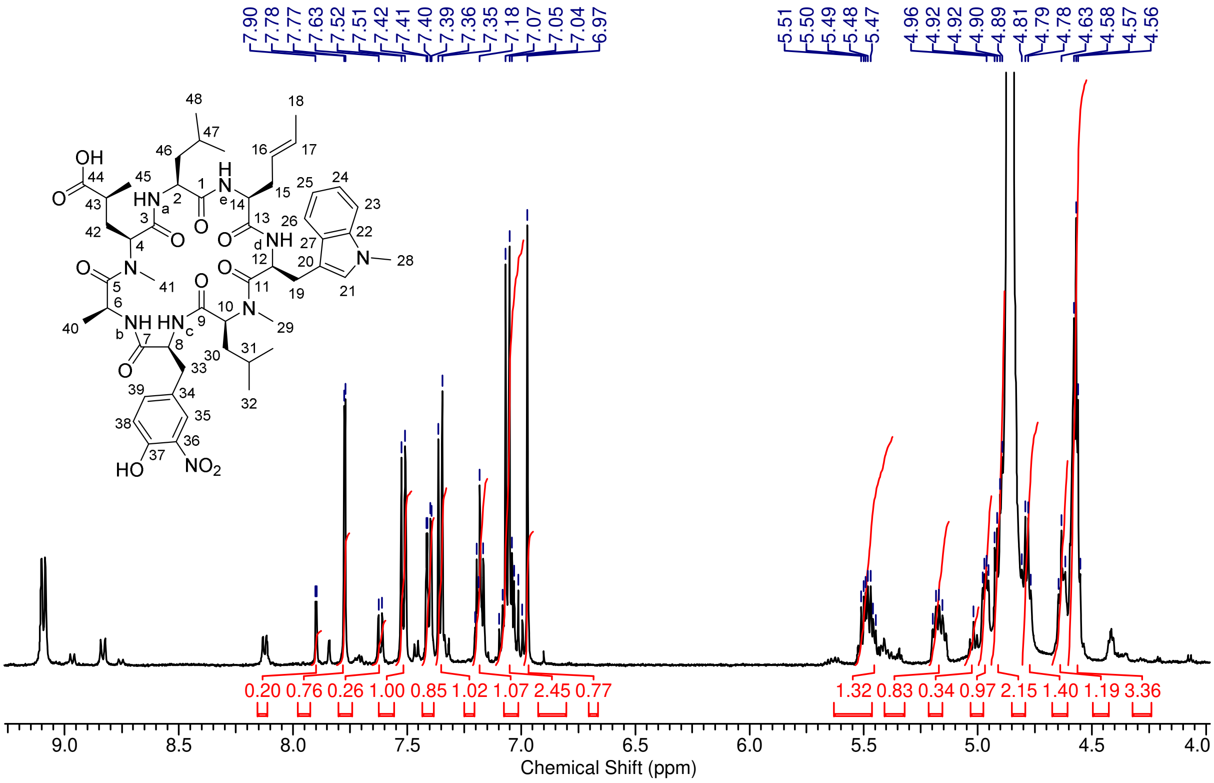

^{13}C-NMR (125 MHz, CD_3OD):

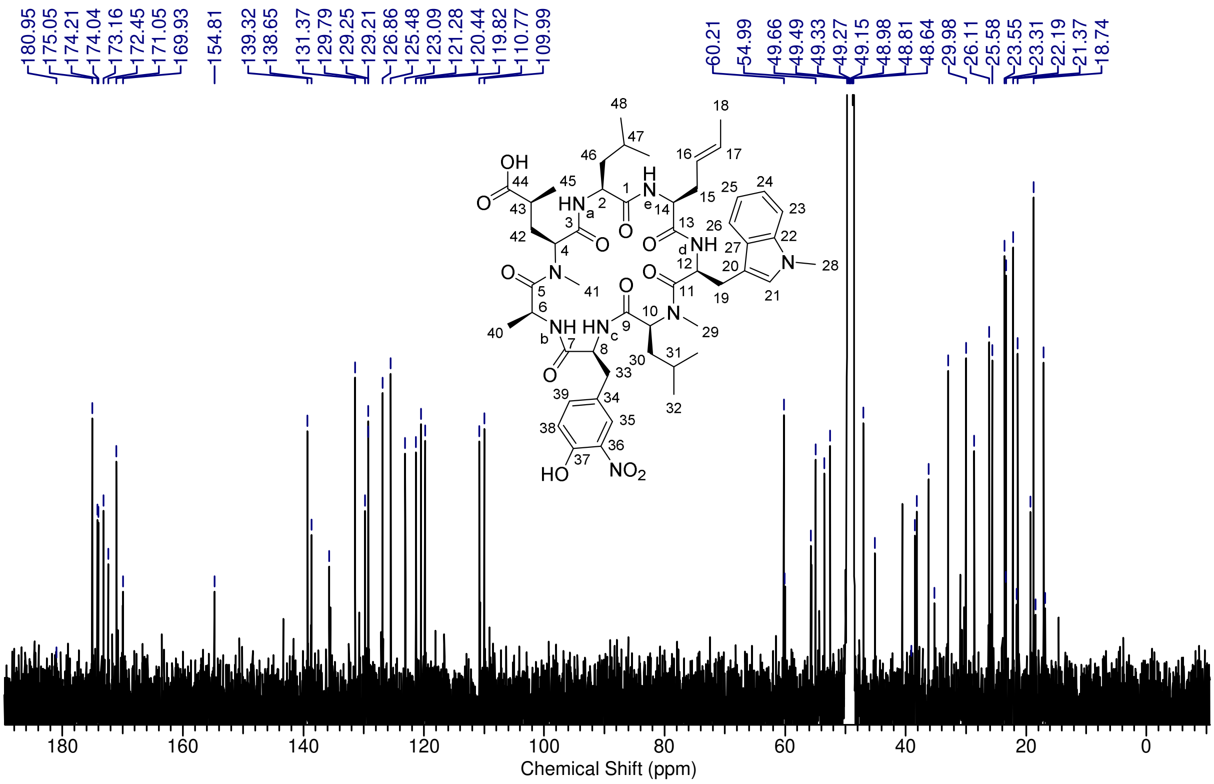

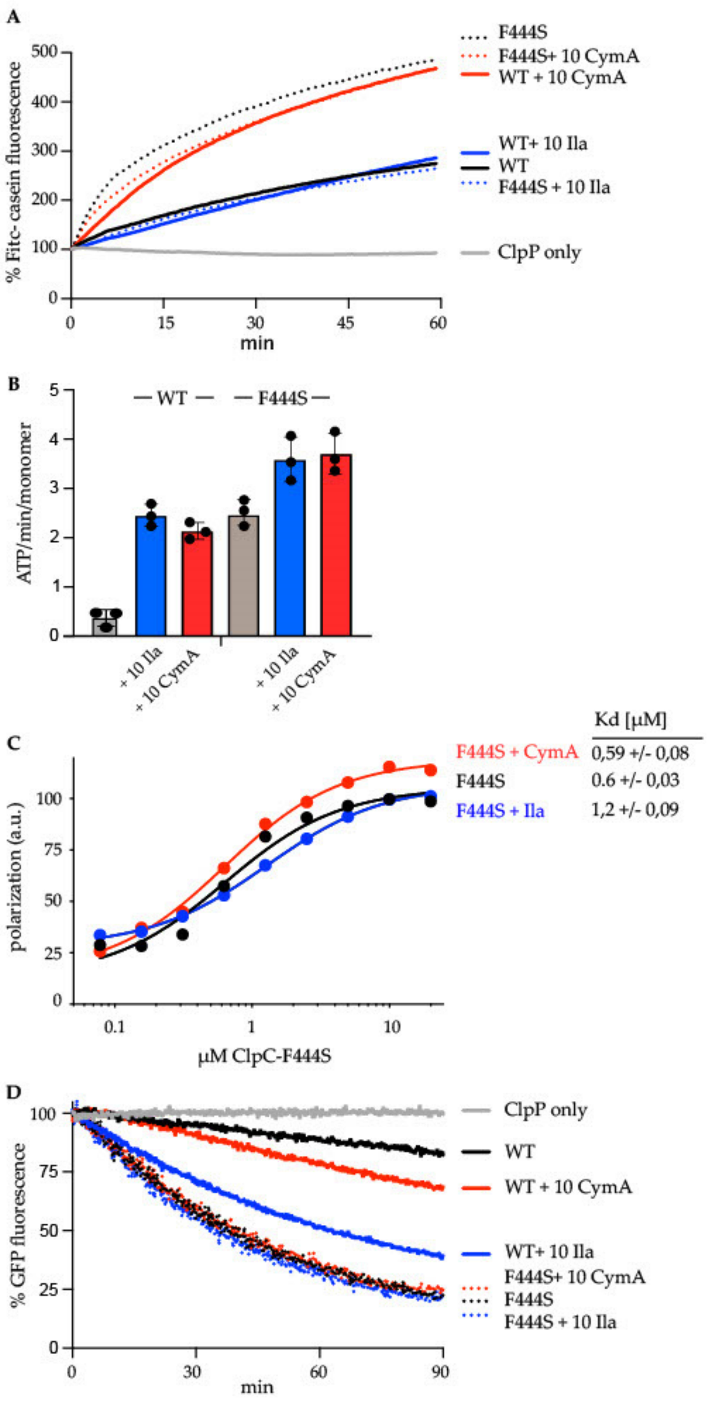

Supplementary Figure S1

Ilamycin (Ila) (**26**) deregulates ClpC1 activities. (A) FITC-casein degradation by ClpC1-WT and ClpC1-F444S was monitored in the presence of ClpP and indicated components (10 µM Ilamycin (Ila) or CyclomarinA (CymA)). Initial FITC-casein fluorescence was set to 100. FITC-casein degradation is linked to increase in fluorescence intensity, which is quenched in full-length casein. (B) ATPase activities of ClpC1-WT and ClpC1-F444S were determined in presence of ClpP and indicated components (as in (A)). (C) Binding of ClpC1-F444S to FITC-casein was monitored in presence of ATPγS and Ilamycin (Ila) or Cyclomarin A (CymA) by determining changes in FITC-casein anisotropy. Calculated binding affinities are provided. (D) GFP-SsrA degradation was monitored by determining the loss of GFP fluorescence in presence of ClpC1-WT or ClpC1-F444S and ClpP in presence of indicated components (as in (A)).
